# Advanced preparation of fragment libraries enabled by oligonucleotide-modified 2′,3′-dideoxynucleotides

**DOI:** 10.1038/s42004-022-00649-9

**Published:** 2022-03-16

**Authors:** Justina Medžiūnė, Žana Kapustina, Simona Žeimytė, Jevgenija Jakubovska, Rūta Sindikevičienė, Inga Čikotienė, Arvydas Lubys

**Affiliations:** 1 Department of Research and Development, Thermo Fisher Scientific Baltics, Vilnius, LT-02241 Lithuania; 2 Faculty of Chemistry and Geosciences, Vilnius University, Vilnius, LT-03225 Lithuania; 3 Institute of Biosciences, Life Sciences Center, Vilnius University, Vilnius, LT-10257 Lithuania

**Keywords:** Chemical modification, DNA

## Abstract

The ever-growing demand for inexpensive, rapid, and accurate exploration of genomes calls for refinement of existing sequencing techniques. The development of next-generation sequencing (NGS) was a revolutionary milestone in genome analysis. While modified nucleotides already were inherent tools in sequencing and imaging, further modification of nucleotides enabled the expansion into even more diverse applications. Herein we describe the design and synthesis of oligonucleotide-tethered 2′,3′-dideoxynucleotide (dd^ON^NTP) terminators bearing universal priming sites attached to the nucleobase, as well as their enzymatic incorporation and performance in read-through assays. In the context of NGS library preparation, the incorporation of dd^ON^NTP fulfills two requirements at once: the fragmentation step is integrated into the workflow and the obtained fragments are readily labeled by platform-specific adapters. DNA polymerases can incorporate dd^ON^NTP nucleotides, as shown by primer extension assays. More importantly, reading through the unnatural linkage during DNA synthesis was demonstrated, with 25-30% efficiency in single-cycle extension.

## Abstract (web summary)

Next-generation genome sequencing technologies have revolutionized the life sciences, however all sequencing platforms require nucleic acid pre-processing to generate suitable libraries for sequencing. Here, oligonucleotide-tethered 2′,3′-dideoxynucleotide terminators bearing universal priming sites are synthesised and incorporated by DNA polymerases, allowing integration of the fragmentation step into the library preparation workflow while also enabling the obtained fragments to be readily labeled by platform-specific adapters.

## Introduction

**Fig. 1:**
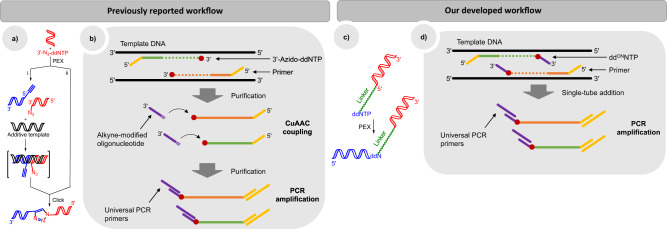
Comparison of library preparation strategies. Previously reported workflow: **a** schematic representation of chemical ribose-to-ribose ligation: i) templated click reaction; ii) non-templated click reaction, **b** workflow for NGS library preparation using ribose-to-ribose ligation. Our reported workflow: **c** schematic ligation, PEX/termination by **dd**^**ON**^**NTP** and **d** NGS library preparation workflow using **dd**^**ON**^**NTP**.

We are witnessing rapid development and fast diffusion of next-generation sequencing (NGS) technologies for almost two decades. The establishment of NGS techniques revolutionized research abilities in modern biology and biomedical sciences. All current sequencing platforms require nucleic acid pre-processing to generate library suitable for sequencing. Generally this includes DNA or RNA fragmentation to a platform-specific size range, followed by end polishing and specific adapter ligation to the 3′ and 5′ termini^1,2^. Enzymatic ligation of adapters is notorious for low efficiency^3^ leading to decreased complexity of original library and impoverishment of sequencing results. Consequently, novel library preparation techniques need to be developed to improve the conversion efficiency and simplify the workflow. Some alternative chemical ligation techniques based on the formation of ribose-to-ribose connection were reported (Fig. 1a)^4,5^. Applying 3′-azido-2′,3′-dideoxynucleotides as terminators for alternative fragment generation the azido group-bearing DNA fragments have been obtained. Followed by the addition of 5′-alkynyl oligonucleotides via Cu(I)-catalyzed Huisgen’s [3 + 2] azide-alkyne cycloaddition, known as click chemistry, chemical ligation was used to form triazole-linkage derivatives and introduce sequencing adapters into DNA fragments (Fig. 1a)^6,7^. However, this strategy exhibited several drawbacks: (1) after fragmentation, additional purification prior to chemical ligation was necessary to remove the residual free 3′-azido-ddNTP; (2) for efficient click reaction a complementary template to 3′-azido oligonucleotide was necessary, therefore it was impossible to implement for unknown sequences; (3) non-templated chemical ligation required high DNA amount which is far from practical for many NGS applications; (4) to avoid Cu-induced inhibition of polymerases, additional purification had to be performed after chemical ligation; (5) more than half of the obtained reads were shorter than expected, caused by Cu-mediated DNA degradation;^7^ and (6) the read-through efficiency was low, as single primer extension cycle generated undetectable amount of product (less than 4%) (Fig. 1a, b). Previous studies revealed the importance of DNA backbone structure for oligonucleotide recognition by polymerase, due to hydrogen bonding^8–10^. Triazole is considered a phosphate-mimicking group given its similar size and the ability to form hydrogen bonds^8^. These studies have shown the possibilities to design and successfully use nucleic acids with artificial backbones in molecular biology applications.

Base-modified nucleotides are widely used as carriers of tags and labels for diagnostics, chemical biology and bioprocess monitoring^11–13^. Apart from relatively small molecular labels, such as redox^14–17^, spin^18,19^ labels and reactive groups (for post-enzymatic modification)^20–23^, synthesis of biomolecule-modified (oligonucleotides^24–26^ and proteins^27^) nucleotides have been also reported. Crystallographic structure analysis reveals that certain modifications partially enter the polymerase active site^11^, thus suitable nucleobase and label-linker design plays a pivotal role. Usually, the main challenge is to avoid steric hindrance caused by the label during the enzymatic nucleotide incorporation. However, the linker is usually not adapted for bridging function in enzymatic read-through. We hypothesized that the ability to read through the linker would enable the use of oligonucleotide modification as a priming site for subsequent synthesis of a complementary strand. This, in turn, would make templates containing oligo-tethered terminators biocompatible, e.g. serve as a template in PCR. In this study, we present oligonucleotide-tethered 2′,3′-dideoxynucleotide (**dd**^**ON**^**NTP**) analogues for labeling of any DNA strand with a universal priming site (Fig. 1c, d).

## Results and discussion

### Chemical synthesis of dd^ON^NTPs

**Fig. 2:**
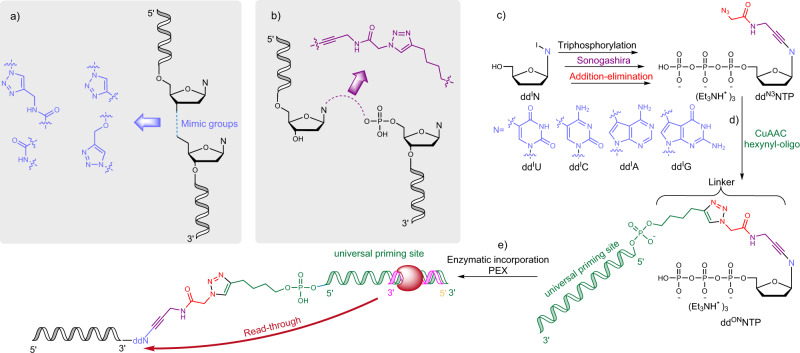
The general principles of chemical conjugation of nucleic acids. **a** Previously reported ribose-to-ribose conjugated analogues, **b** our proposed nucleobase-phosphate conjugation analogues, **c** synthesis of azido group-bearing ddNTPs, **d** oligo-tethered ddNTP derivatives and **e** **dd**^**ON**^**NTP** enzymatic incorporation and read-through.

Several reported articles describe chemical ligation approach for the oligonucleotide conjugation via ribose-to-ribose connection^7,8,10^. In the attempts to design chemical linkage which orthogonally resembles natural DNA several triazole-based phosphodiester mimic groups were applied (Fig. 2a). The use of triazole as a phosphodiester analogue has been well established in antisense oligonucleotides technology, since it stabilizes nucleic acid and increases the interaction with the complementary strand^28–30^. Even though the majority of these linkages were tolerated in the read-through assays, in many cases they caused insertion/deletion events, as well as poor read-through efficiency^6,7,10^. Besides indicating the importance of the proximity between 5′ and 3′ sugar rings, authors also point out the lack of linker’s flexibility. During enzymatic synthesis flexibility is critical, as the polymerase has to twist the template backbone by ~90°^31,32^ and the rigidness of the linker may cause deletions^10^. We propose a different approach to bridge oligonucleotides - via nucleobase-to-ribose connection (Fig. 2b) that might provide more flexibility during read-through and allows a seamless synthesis of the complementary strand.

Several important aspects of linker design were considered: whilst the linker had to be long enough to avoid label-induced steric hindrance during enzymatic incorporation, it also had to be length-compatible for polymerase to read through it. Considering all that, we designed functionalized azido-ddNTPs (**dd**^**N3**^**NTP**) with desired linker properties, as substrates for Cu (I)-catalyzed alkyne-azide cycloaddition (CuAAC) with alkyne-oligonucleotides. An efficient and straightforward synthesis strategy of the **dd**^**N3**^**NTP**s has been developed (Fig. 2c, for more details see Supplementary Fig. S1). Our scheme consists of triphosphorylation, aqueous phase Sonogashira coupling^33,34^, and acylation by the azidoacetic acid NHS ester cascade. This chemistry allowed to shorten the synthesis of modified ddNTPs to three steps, whereas the conventional path is at least five steps-long^12,34–36^. Propargylamine was chosen for linker formation for several reasons: it can be easily applied for C-C bond formation via the Pd-catalyzed coupling, more importantly, the reactive amine group may serve for further modification, while the triple bond next to the heterocyclic base unit is not reactive in the following click chemistry^37^. Finally, the desired **dd**^**ON**^**NTP**s were synthesized via the CuAAC reaction between **dd**^**N3**^**NTP** and 5′-hexynyl-oligonucleotide (Fig. 2d, for exact structure of 5′-hexynyl-oligonucleotide see Supplementary Fig. S2). This selective and potent method has quite a few advantages: the precursors of the azide and alkyne moieties can be either synthesized or purchased; CuAAC is compatible with aqueous media and results in the formation of highly stable triazole-linkage entity^38^. Notably, the most critical part of the **dd**^**ON**^**NTP** preparation was the purification steps. Due to highly sensitive enzymatic reactions, even the trace amount of additives used in chemical synthesis, such as Cu, would lead to inhibition^39,40^. Moreover, we observed that **dd**^**N3**^**NTP**s that bear sterically smaller group are significantly better substrates for DNA polymerases (Klenow exo-, Thermo sequenase, Phusion exo-, TdT, Superscript IV) than the **dd**^**ON**^**NTP**s with their bulky ON-substituents. During enzymatic termination the **dd**^**N3**^**NTP**s would be preferred over the **dd**^**ON**^**NTP**s reducing the efficiency of the labeling by oligonucleotide. Therefore, efficient purification was required to avoid contamination of polymerase reaction by the precursors (**dd**^**N3**^**NTP**s) used in click chemistry. Reverse-phase chromatography was applied and HPLC methods were developed to purify purine and pyrimidine derivatives (for more synthesis and purification details see Supplementary Methods).

### Enzymatic incorporation of dd^ON^NTPs

**Fig. 3:**
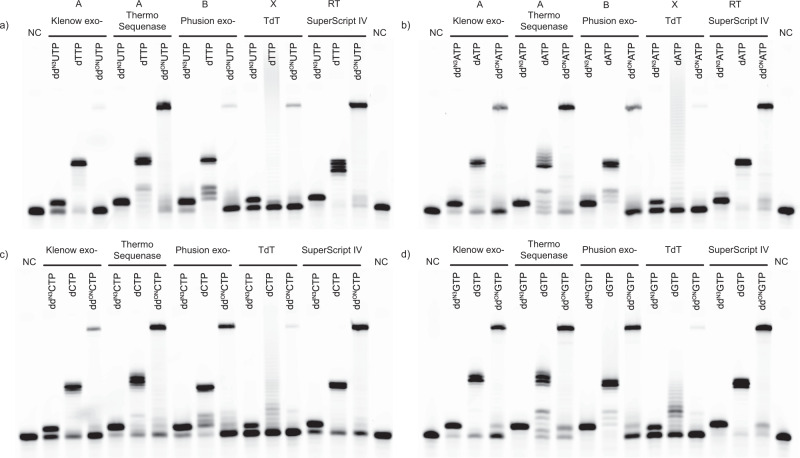
**dd**^**ON**^**NTP** incorporation by DNA polymerases. Electropherograms showing PEX results obtained using various DNA polymerases with either **dd**^**N3**^**NTPs**, dNTPs, or **dd**^**ON**^**NTPs** in the presence of the following templates: **a** Dup^A^, **b** Dup^T^, **c** Dup^G^, **d** Dup^C^. NC - negative control.

After **dd**^**N3**^**NTP**s and **dd**^**ON**^**NTP**s were synthesized and purified primer extension experiments were run (Fig. 2e) using polymerases of families A, B, X and RT (Fig. 3). In general, DNA polymerases are classified into seven families (A, B, C, D, X, Y and RT) according to their crystallographic structure and sequence homology^41^, which could lead to their different performance with our substrates. Oligonucleotide duplex which served as a template contained 10 nt overhang. Hence, during primer extension reaction (PEX) in the presence of dNTPs up to 10 nucleotides could be added, while the incorporation of **dd**^**ON**^**NTP** would allow to elongate the primer by 23 nt (see Methods section). All tested polymerases were 3′-5′ exonuclease-deficient and accepted **dd**^**N3**^**NTP**s and **dd**^**ON**^**NTP**s as substrates. The efficiency of **dd**^**ON**^**NTP** incorporation varied between polymerases. In addition, a slight preference for terminator’s nucleobase was observed. Klenow exo- exhibited better incorporation efficiency with oligo-modified 2′,3′-dideoxypurines rather than pyrimidines, and similar trend was observed with **dd**^**N3**^**NTP** derivatives. Thermo Sequenase, another DNA polymerase from family A, was able to incorporate all types of **dd**^**N3**^**NTP**s and **dd**^**ON**^**NTP**s with superior efficiency compared to other tested enzymes, since it does not discriminate between dNTPs and ddNTPs^42^.

Phusion™ exo- DNA polymerase from family B catalyzed the addition of **dd**^**ON**^**NTP**s with lower efficiency compared to Thermo Sequenase, especially in the case of **dd**^**ON**^**UTP**. Terminal deoxynucleotidyl transferase (TdT) performed with the lowest efficiency, although TdT is known to tolerate bulky base modifications^43^. This result could be attributed to suboptimal reaction conditions, such as the **dd**^**ON**^**NTP**-to-template ratio. The **dd**^**ON**^**NTP** addition by SuperScript™ IV reverse transcriptase (RT) had similar efficiency as the Thermo Sequenase (Fig. 3). In practice an **dd**^**ON**^**NTP**-compatible RT enzyme could enable cDNA labeling, while a thermostable DNA polymerase could be used in nucleic acid analysis workflows with ssDNA or dsDNA templates.

### Semi-targeted sequencing

In most applications **dd**^**ON**^**NTP**-labeled DNA fragments would be subjected to PCR amplification, thus we looked for thermostable DNA polymerase that would synthesize through an unnatural linkage. We’ve tested Phusion exo- DNA polymerase and found that it was able to build complementary DNA strand using oligonucleotide modification as a priming site, with a single-cycle read-through efficiency of 25–30% (Supplementary Fig. S3). The observed polymerase stalling near the linker position in a single-cycle reaction and somewhat better read-through efficiency with a prolonged extension time suggested that the read-through phase might be a rate-limiting step. When 15 linear cycles of the extension were performed allowing the polymerase multiple attempts to bypass the linker, the amount of full-length product increased substantially.

**Fig. 4:**
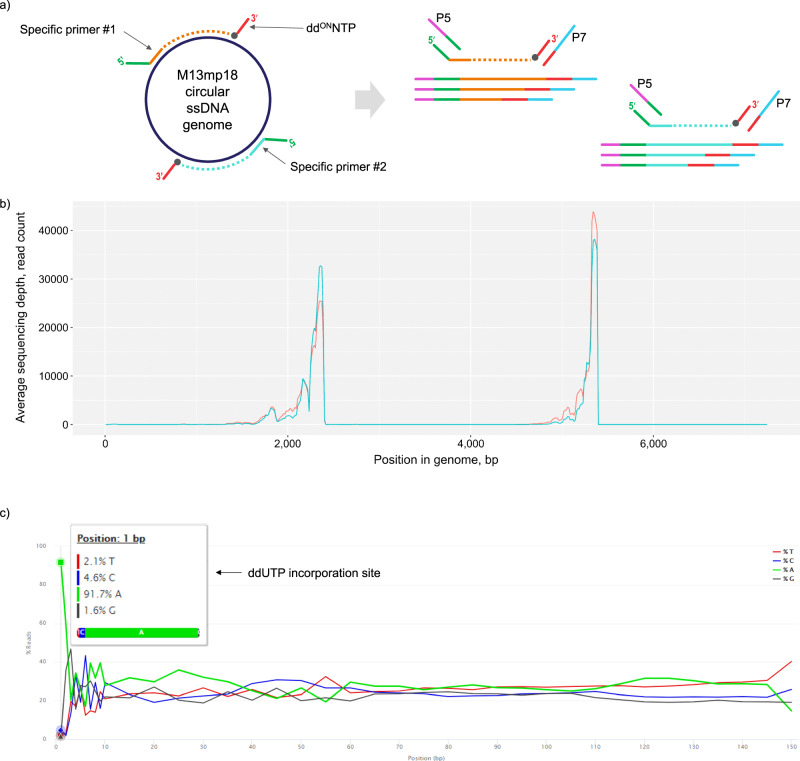
Semi-targeted sequencing of the M13mp18 viral genome. **a** A schematic overview of library preparation with **dd**^**ON**^**NTP**s. **b** M13mp18 genome coverage. The reads concentrated at two loci with one terminus of sequenced inserts fixed at the specific priming sites. Another terminus corresponds to the stochastic positions of **dd**^**ON**^**UTP** incorporation. The orange and blue lines represent technical replicates. **c** Base composition of sequenced reverse reads. The dominance of A base at the first position indicates a seamless copying of the template around the linker position.

The approach proposed herein offers a significantly simplified NGS library preparation protocol with nucleic acid fragmentation and labeling integrated into a single enzymatic step (Fig. 4a). To demonstrate the applicability of our technology, we applied **dd**^**ON**^**UTP**s and identified incorporating and replicating polymerases for the generation of fragment libraries compatible with Illumina next-generation sequencing. We designed primers specific to two loci within M13mp18 genome and performed a linear primer extension in the presence of Thermo Sequenase enzyme and a mixture of dNTPs and **dd**^**ON**^**UTP** (see Methods section). Upon incorporation, **dd**^**ON**^**UTP** terminated DNA synthesis and simultaneously labeled the nascent DNA strand with a partial Illumina adapter sequence attached to its nucleobase. This step generated fragments of suitable length for short read sequencing flanked by both adapters. The obtained sequencing data indeed indicated capture of two genomic loci with a characteristic insert structure: fixed terminus corresponded to the specific priming site, while randomly distributed terminus illustrated the stochastic nature of **dd**^**ON**^**UTP** incorporation (Fig. 4b). The inspection of sequencing reads has not revealed any systematic errors or artefacts which could be associated with the presence of unnatural backbone within the fragment library. The analysis of base distribution per sequenced position showed clear dominance (>90%) of A base at the first position of reverse reads (Fig. 4c), which corresponds to ddUTP incorporation site. This indicates that the vast majority of sequenced molecules derived from a seamless read-through, i.e. the copying polymerase incorporated a complementary nucleotide immediately downstream of the **dd**^**ON**^**UTP** linker. To compare, 69% of the reads indicated correct read-through in library preparation method based on chemical ligation. The authors reasoned that the presence of an incorrect base at the position downstream of the chemical linkage was a result of substitution events^7^. As the termini of reverse reads in our sequencing experiment aligned to the reference genome even in the case when the first base is not A, imperfect read-through likely originated from base skipping leading to deletions. Importantly, this rare effect does not impair the analysis of sequencing data and does not require additional trimming of sequencing reads.

We termed the developed library preparation technique semi-targeted sequencing emphasizing that only a single primer is needed to capture a desired locus, while **dd**^**ON**^**NTP**s can label primer extension products irrespective of the sequence context of the template downstream of the priming site. Somewhat similar targeted library preparation approaches involving single primer extension and open-ended amplification were previously reported in the field, including multiplexed primer extension sequencing^44^ and anchored multiplex PCR^45^. Both methods proved to be feasible, however lengthy multistep workflows with intermediate purification steps that inevitably lead to the loss of material are not attractive for adoption in settings where sample preparation speed is important. The addition of sequencing adapters to DNA strands of unknown sequence is achieved via separate fragmentation, end repair and enzymatic ligation of synthetic oligonucleotides. **dd**^**ON**^**NTP**s open the doors to obtain equivalent results with a simple and rapid workflow that integrates fragment generation and labeling. In addition, efficient replication through the **dd**^**ON**^**NTP** linker preserves library complexity and implies the compatibility of our approach with the analysis of complex genomes or transcriptomes.

## Conclusions

In conclusion, we have designed and synthesized oligonucleotide-modified 2′,3′-dideoxynucleotide terminators, with properties that go beyond commonly labeled nucleotides. Represented efficient **dd**^**ON**^**NTP**s synthesis and purification strategy enables to obtain these complex molecules with necessary qualities for enzymatic processes. We showed that, even though, their structure possesses bulky oligonucleotide label, the linker design empowers both the enzymatic incorporation and read-through. We demonstrated that modified template replication not necessarily requires mimicking of the phosphodiester group of oligonucleotides. Nucleobase-to-ribose conjugation performed with accuracy and satisfying efficiency opened new possibilities for further linker development. This enabled us to integrate the fragmentation and adapter addition into a single enzymatic step, thus substantially simplifying sample preparation workflow for high-throughput sequencing. The biocompatibility of 2-(4-butyl-1*H*-1,2,3-triazol-1-yl)-N-(prop-2-ynyl)acetamide artificial backbone paves the way for numerous applications, such as whole-genome and whole-transcriptome fragment library preparation for NGS analysis, semi-targeted library preparation with great potential to investigate gene fusion events or characterize microbial communities^46^, nucleic acid labeling for subsequent detection employing universal priming site, and others that benefit from the addition of a known artificial sequence to DNA or cDNA. Notably, oligonucleotide modification can contain further modifications, such as biotin or phosphate to enable specific capture of **dd**^**ON**^**NTP**-containing molecules or to block 3′ terminus, respectively.

## Methods

### General

All chemicals and solvents were obtained commercially and used without further purification. Iodinated nucleosides dd^I^U, dd^I^C, dd^I^A, dd^I^G were obtained from AEchem Scientific. Moisture sensitive reactions were performed under dry argon atmosphere using oven-dried glassware. Reactions, fractions and final purities were monitored by Vanquish^TM^ UHPLC system using TSKgel^TM^ ODS- 100V 3 µm column (mobile phases: A – 100 mM TEAAc, B – 40% 100 mM TEAAc + 60% ACN). Modified nucleotides were purified by Biotage^TM^ Isolera^TM^ One or Biotage™ Isolera^TM^ Prime liquid chromatography systems with UV detectors using Q Sepharose™ Fast Flow, Biotage SNAP ULTRA C18 columns. Purification of oligonucleotide-tethered 2′,3′-dideoxynucleotides was held with Waters 2555 Quaternary gradient system using YMC-Actus Triart C18 100 × 20 mm S–5 µm column, while product for read-through efficiency measurements was purified by Vanquish UHPLC system using YMC-Triart C18 150 × 4.6 mm S–3 µm column. Conductivity was measured with Mettler-Toledo™ FiveGo^TM^ F3 conductometer (prior to ion exchange chromatography reaction mixtures were diluted to 3.5–4 mS/m2 conductivity). Absorbance of products were measured by Thermo Fisher Evolution^TM^ 201 UV-Visible spectrophotometer. Oligonucleotide-tethered nucleotides amount was determined using NanoDrop^TM^ 2000 spectrophotometer. Monoisotopic masses measurements were performed using triple quadrupole mass spectrometer TSQ Endura^TM^ with ESI ion source, oligonucleotides molecular masses were determined using hybrid Quadrupole-Orbitrap^TM^ mass spectrometer Q Exactive plus^TM^ (Thermo Fisher Scientific). NMR spectra were recorded using Bruker Avance III 400 MHz (9.0 T). Chemical shifts are given in parts per million relative to tetramethylsilane using residual solvents signals as internal standards. Signal patterns are indicated as br, broad; s, singlet; d, doublet; t, triplet; q, quartet; m, multiplet. The complete assignment of 1H and 13C signals was performed by an analysis of the correlated homonuclear H,H-COSY, and heteronuclear H,C-HSQC and H,C-HMBC spectra.

All enzymes and buffers were manufactured by Thermo Fisher Scientific unless specified otherwise. Oligonucleotides were synthesized by Metabion GmbH requesting HPLC purification.

### Chemical synthesis

#### General procedure for CuAAc click reaction

All reaction components were applied to the reaction mixture as solutions in water unless specified differently. Corresponding **dd**^**N3**^**NTP** (3 eq., 2–4 mM) solution was added to alxyl-oligonucleotide (5′-hexynyl-AGATCGGAAGAGCACACGTCTG-3′-biotin) (100–210 nmol) solution in sodium phosphate buffer (1 ml, 100 mM, pH 7). In a separate vial CuSO_4_ (100 mM, 12 eq.) and THPTA (250 mM, 5 eq. to CuSO_4_) were premixed and added to the reaction mixture, followed by addition of sodium ascorbate (1 M, 50 eq. to CuSO_4_). Reaction mixture was stirred for 20 min at 42 °C, quenched with 0.5 M EDTA-Na_2_ solution (0.5 ml, pH 8). The products were purified by semi-preparative reverse phase HPLC using a linear gradient of 100 mM TEAAc/ACN (11–18% ACN for **dd**^**ON**^**CTP** and **dd**^**ON**^**UTP**, 10–20% ACN for **dd**^**ON**^**ATP** and **dd**^**ON**^**GTP**). **dd**^**ON**^**NTP**s were desalted using water/ACN (5–100%) gradient. The characteristics of **dd**^**ON**^**NTP** are given in Table 1 (for MS and HPLC chromatograms see Supplementary Figs. S4–13).

**Table 1:** Characteristics of dd^ON^NTP after CuAAC click reactions.

dd^ON^NTP	Yield	Purity	M (calcd), Da	M (found) [M], Da
dd^ON^CTP	39%	98%	7916.345	7916.342
dd^ON^UTP	34%	98%	7917.329	7917.320
dd^ON^ATP	29%	94%	7939.352	7939.371
dd^ON^GTP	43%	96%	7955.352	7955.349

### dd^ON^NTP incorporation assays

A selection of various DNA polymerases, including representatives from family A, family B, family X, and reverse transcriptase (RT) family were tested for capability of incorporating **dd**^**ON**^**NTP**. The experimental system was based on the extension of 3′-recessed ends of the oligonucleotide duplexes.

#### Templates for incorporation testing

To prepare oligonucleotide duplexes with different protruding ends, oligonucleotides listed in Table 2 were annealed in 1× annealing buffer (10 mM Tris-HCl pH 8.1 mM EDTA, 50 mM NaCl) to obtain 2 µM final solution. Primers were labeled with a fluorescent dye for subsequent detection of PEX products on a gel.

**Table 2:** Oligonucleotide duplexes used for dd^ON^NTP incorporation testing.

Name	Oligonucleotide duplex
Dup^A^	5′-AAAAAAAAAATACGCCAAGGATGCCTACCCATGTCTGCA-3′
	3′-ATGCGGTTCCTACGGATGGGTACAGACGT-Cy5-5′
Dup^T^	5′-TTTTTTTTTTTACGCCAAGGATGCCTACCCATGTCTGCA-3′
	3′-ATGCGGTTCCTACGGATGGGTACAGACGT-Cy5-5′
Dup^G^	5′-GGGGGGGGGGTACGCCAAGGATGCCTACCCATGTCTGCA-3′
	3′-ATGCGGTTCCTACGGATGGGTACAGACGT-Cy5-5′
Dup^C^	5′-CCCCCCCCCCTACGCCAAGGATGCCTACCCATGTCTGCA-3′
	3′-ATGCGGTTCCTACGGATGGGTACAGACGT-Cy5-5′

#### PEX reaction conditions for incorporation testing

Primer extension reactions were performed in optimal buffers and optimal or near-optimal temperatures for each tested polymerase. Control reactions with native dNTPs and **dd**^**N3**^**NTPs** were conducted to ensure that polymerase of interest is (i) capable to perform conventional primer extension at given conditions and (ii) is able to incorporate ddNTPs with small base modification. Tested polymerases and specific reaction conditions are listed in Table 3. In all cases, 2 pmol of oligonucleotide duplex (or single-stranded primer for TdT) and 20 pmol of **dd**^**ON**^**NTP** or corresponding native dNTP, or **dd**^**N3**^**NTP** were used per reaction.

**Table 3:** Polymerases tested for dd^ON^NTP incorporation.

Family	Polymerase	Amount per reaction	Conditions
A	Thermo Sequenase	40 U	95 °C 1 min → 60 °C 30 min
A	Klenow fragment (exo-)	5 U	37 °C 30 min
B	Phusion (exo-)	20 U	95 °C 1 min → 60 °C 30 min
X	TdT	20 U	37 °C 30 min
RT	SuperScript™ IV	200 U	50 °C 30 min

Reaction products were resolved on 15% TBE-Urea PAGE. Prior to loading on a gel, samples were mixed in a 1:1 ratio with 2× DNA loading buffer (98% formamide, 10 mg/mL blue dextran, 10 mM EDTA), heated at 95 °C for 5 min and then immediately cooled on ice. Electrophoresis was carried out in 1× TBE buffer at 400 V for 1 h at 55 °C. Gels were imaged with Typhoon™ FLA 9500 system (GE Healthcare).

### Read-through assay

For successful application of **dd**^**ON**^**NTP** for nucleic acid detection and analysis, it is highly desirable to identify a thermostable DNA polymerase able to read through the linker – this would make **dd**^**ON**^**NTP**-containing nucleic acids compatible with PCR amplification.

To prepare **dd**^**ON**^**UTP**-containing template (**ON-dd**^**ON**^**U**) for read-through assay, **dd**^**ON**^**UTP** was PEX-incorporated by SuperScript IV RT into the 3′ end of oligonucleotide 5′-TGCAGACATGGGTAGGCATCCTTGGCGTA-3′ annealed to the RNA oligonucleotide 5′-auacgccaaggaugccuacccaugucugca-3′. RNA strand was then digested by RNase H treatment, DNA was purified and desired single-stranded **ON-dd**^**ON**^**U** was purified by HPLC.

Primer of sequence 5′-CAGACGTGTGCTCTTCC-3′ was 5′-radio-labeled by T4 polynucleotide kinase in the presence of [γ-^33^P]-ATP. The 5′-labeled primer was desalted using Zeba™ Spin desalting columns (7K MWCO). In all, 50 fmol of purified **ON-dd**^**ON**^**U** annealed with primer (1.1:1 molar ratio respectively) were mixed with 0.2 mM dNTP mix and 20 U Phusion exo- in 1× of the following buffers or master mixes: (i) Phusion™ HF buffer, (ii) Phusion™ GC buffer, (iii) Phire™ reaction buffer, (iv) SuperFi™ buffer, (v) Invitrogen™ Collibri™ Library Amplification Master Mix, (vi) Invitrogen™ Collibri™ Library Amplification Master Mix treated with thermolabile proteinase K (NEB) to remove SuperFi polymerase. Read-through PEX reactions were executed as follows: denaturation at 95 °C for 1 min, annealing at 60 °C for 1 min and extension at 72 °C for either 1 min or 15 min. In addition, 15 cycles of linear amplification were tested with 1 min extension. Reaction products were resolved on 15% TBE-Urea PAGE. Visualization was carried out by phosphorimaging using the Typhoon™ FLA 9500 imaging system (GE Healthcare).

### Semi-targeted NGS library preparation and sequencing

For proof-of-principle demonstration, M13mp18 bacteriophage single-stranded DNA (Thermo Fisher Scientific) was used as a sample input. Target-specific primers were designed to contain partial Illumina i5 adapter sequence (underlined) at their 5′ termini:

M13-1: 5′-TACACGACGCTCTTCCGATCTAACGGTACGCCAGAATCTTG-3′

M13-2: 5′-TACACGACGCTCTTCCGATCTAGAGCCACCACCGGAAC-3′

0.125 pmol of each primer were mixed with 200 ng of M13mp18 DNA in a 20 µl reaction mixture containing 2 pmol of **dd**^**ON**^**UTP**, 20 pmol of dNTP mix and 40 U of Thermo Sequenase with thermostable pyrophosphatase in 1× Thermo Sequenase reaction buffer. Primer extension was executed as follows: denaturation at 95 °C for 30 s, followed by 15 cycles of denaturation at 95 °C for 30 s, annealing/extension at 60 °C for 2 min and final extension at 60 °C for 30 min

Half of the primer extension reaction was used directly for indexing PCR. 10 µl of primer extension reaction mixture was combined with 25 µl of Invitrogen Collibri Library Amplification Master Mix (Thermo Fisher Scientific), 20 U of Phusion exo- (1 µl), 5 µl of indexing primers i5 and i7 (50 pmol each) of the sequences given below and 9 µl of nuclease-free water.

i5 primer: 5′-AATGATACGGCGACCACCGAGATCTACACTCTTTCCCTACACGACGCTCTTCCGATCT-3′

i7 primer: 5′-CAAGCAGAAGACGGCATACGAGAT[8nt index]GTGACTGGAGTTCAGACGTGTGCTCTTCCGATCT-3′

Cycling was performed as follows: denaturation at 98 °C for 30 s, followed by 20 cycles of denaturation at 98 °C for 10 s, annealing at 60 °C for 30 s, extension at 72 °C for 1 min, and final extension at 72 °C for 1 min. PCR products were then purified using Dynabeads™ Cleanup Beads (Thermo Fisher Scientific). DNA binding to the beads was performed by mixing 45 µl of bead suspension with 50 µl of sample and subsequent incubation at room temperature for 5 min. Sample was then placed on magnet, supernatant was removed and beads were resuspended in 50 µl of elution buffer containing 10 mM Tris-HCl (pH 8). 50 µl of fresh beads were added again to the sample and binding was repeated. After room temperature incubation, sample was placed on magnet, supernatant was removed, and beads were washed twice with 85% ethanol. To elute libraries, beads were resuspended in 22 µl of elution buffer and incubated for 1 min at room temperature. Fragment size distribution was then assessed by Agilent™ 2100 Bioanalyzer™ system (Agilent Technologies) with High Sensitivity DNA Kit. Quantification of sequenceable molecules was performed with Invitrogen Collibri Library Quantification Kit (Thermo Fisher Scientific).

Libraries were sequenced on the Illumina™ MiSeq™ instrument using the MiSeq Reagent Kit v2 (300-cycle) at 2 × 150 bp paired-end mode.

### Reporting summary

Further information on research design is available in the Nature Research Reporting Summary linked to this article.

## Supplementary information

Supplementary information

Reporting Summary

## Data Availability

All data that support the findings of this study have been provided in the manuscript and Supplementary Information file or could be obtained from the corresponding author on reasonable request. Supplementary Information provides general experimental procedure for synthesis of **dd**^**I**^**NTP**s, **dd**^**PA**^**NTP**s, **dd**^**N3**^**NTP**s, read-through product – **ON-dd**^**ON**^**U** (for MS and HPLC chromatograms see Supplementary Figs. S14–15), NMR (Supplementary Figs. S16–98) and HRMS data for selected compounds.
