# Supplementary material for: Advanced preparation of fragment libraries enabled by oligonucleotide-modified 2′,3′-dideoxynucleotides
Source: Commun Chem. 2022 Mar 16;5:34. doi: 10.1038/s42004-022-00649-9 (PMC9814608; doi:10.1038/s42004-022-00649-9)
Supplement: Supplementary file 1 — Supplementary information [file 42004_2022_649_MOESM1_ESM.pdf]

Supplementary information

Advanced preparation of fragment libraries enabled by oligonucleotide-modified 2',3'-dideoxynucleotides

Justina Medžiūnė, [ab*+] Žana Kapustina, [ac+] Simona Žeimytė,[a] Jevgenija Jakubovska,[a] Rūta Sindikevičienė, [a] Inga Čikotienė [ab] and Arvydas Lubys [a]

[a] Department of Research and Development, Thermo Fisher Scientific Baltics, Vilnius, LT-02241, Lithuania

[b] Faculty of Chemistry and Geosciences, Vilnius University, Vilnius, LT-03225, Lithuania

[c] Institute of Biosciences, Life Sciences Center, Vilnius University, Vilnius, LT-10257, Lithuania

[+] These authors Justina Medžiūnė and Žana Kapustina contributed equally.

Table of Contents

SUPPLEMENTARY METHODS **3**

1. Synthesis scheme for dd^{ON}NTPs 3
2. Polymerases read-through dd^{ON}NTP linker efficiency 4
3. Experimental Procedures 4
   - 3.1. Preparation of dd^{ON}UTP for read-through assay 11
   - 3.2. Purification of ON-dd^{ON}U – read-through product 11
   - 3.3. HPLC and mass spectra of dd^{ON}NTPs and ON-dd^{ON}U 11

SUPPLEMENTARY NOTE **18**

1. NMR spectra 18

Supplementary Methods

1. Synthesis scheme for dd^{ON}NTPs

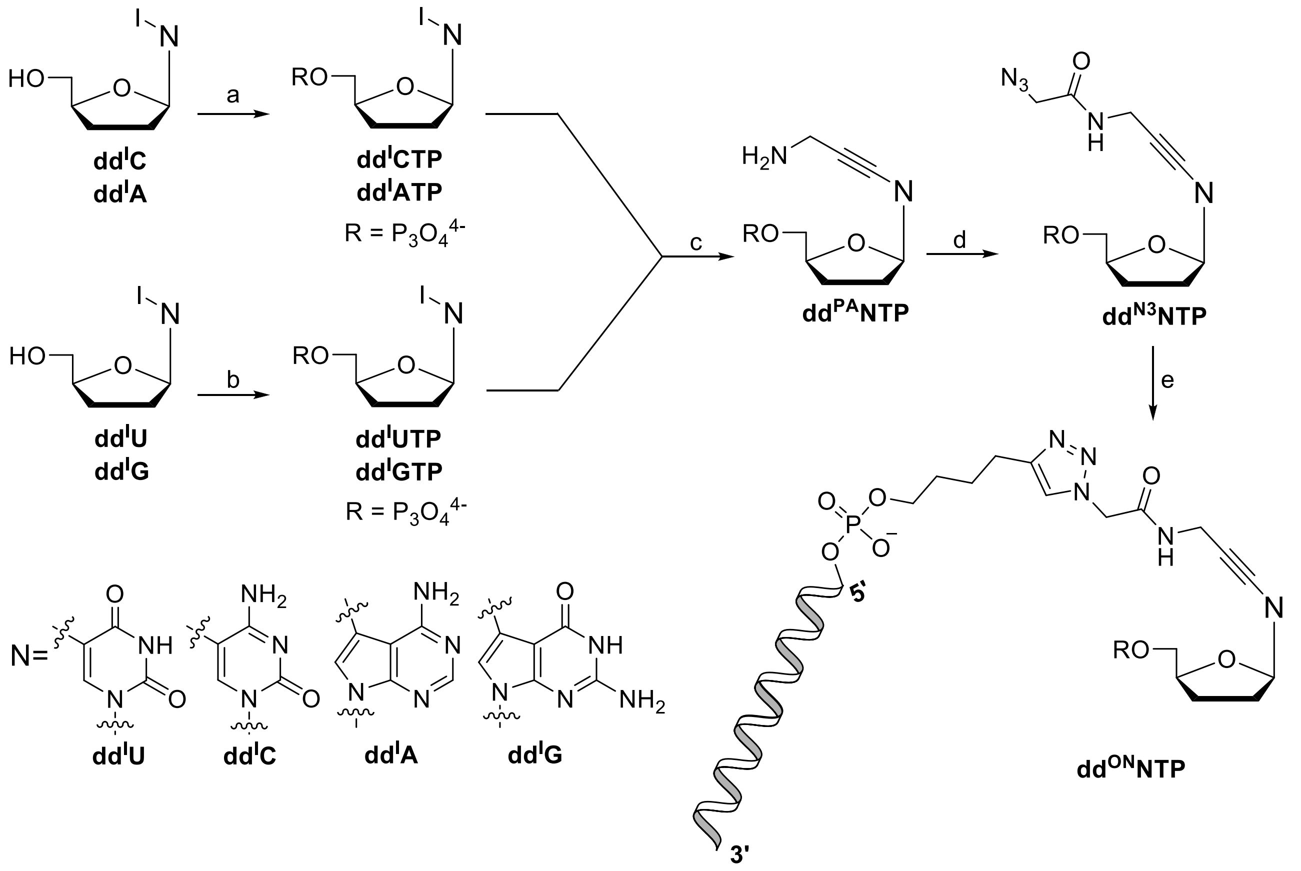

Supplementary Figure S1. Synthesis of modified dd^{ON}NTPs. Reagents and conditions: a) 1. $P_2O_3Cl_4$, ACN, 30 min (in case of **dd^{I}C** – (-15) – (-10) ^{o}C; while **dd^{I}A** – 0 ^{o}C); 2. $(n\text{-}Bu_3NH)_2H_2P_2O_7$, NBu_3, ACN, 30 min, rt; 3. 1 M TEAB, rt; b) 1. NBu_3, $POCl_3$, $PO(OMe)_3$, 2 h, 0 ^{o}C; 2. $(n\text{-}Bu_3NH)_2H_2P_2O_7$, NBu_3, ACN, 30 min, rt; 3. 1 M TEAB, rt; c) propargylamine, CuI, $Pd(OAc)_2$, TPPTS, H_2O/ACN, 1 – 2 h, 40 ^{o}C; d) azidoacetic acid NHS ester, $Na_2CO_3/NaHCO_3$ (pH 9)/DMF, 30 min, rt; e) 5'-hexynyl-oligonucleotide, $CuSO_4$, THPTA, sodium ascorbate, sodium phosphate buffer (pH 7), 42 ^{o}C, 20 min.

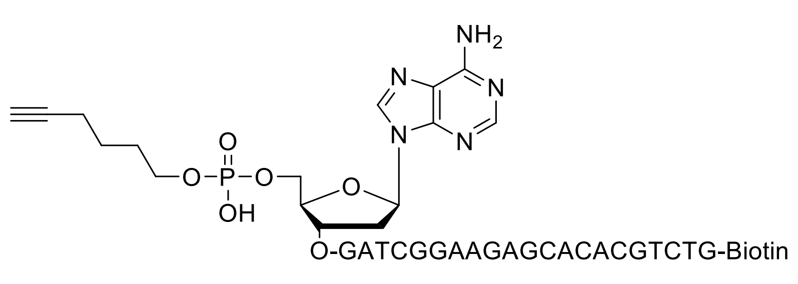

Hexynyl-oligonucleotide

Supplementary Figure S2. Structure and sequence of hexynyl-oligonucleotide applied for CuAAC click reactions

2. Polymerases read-through dd^{ON}NTP linker efficiency

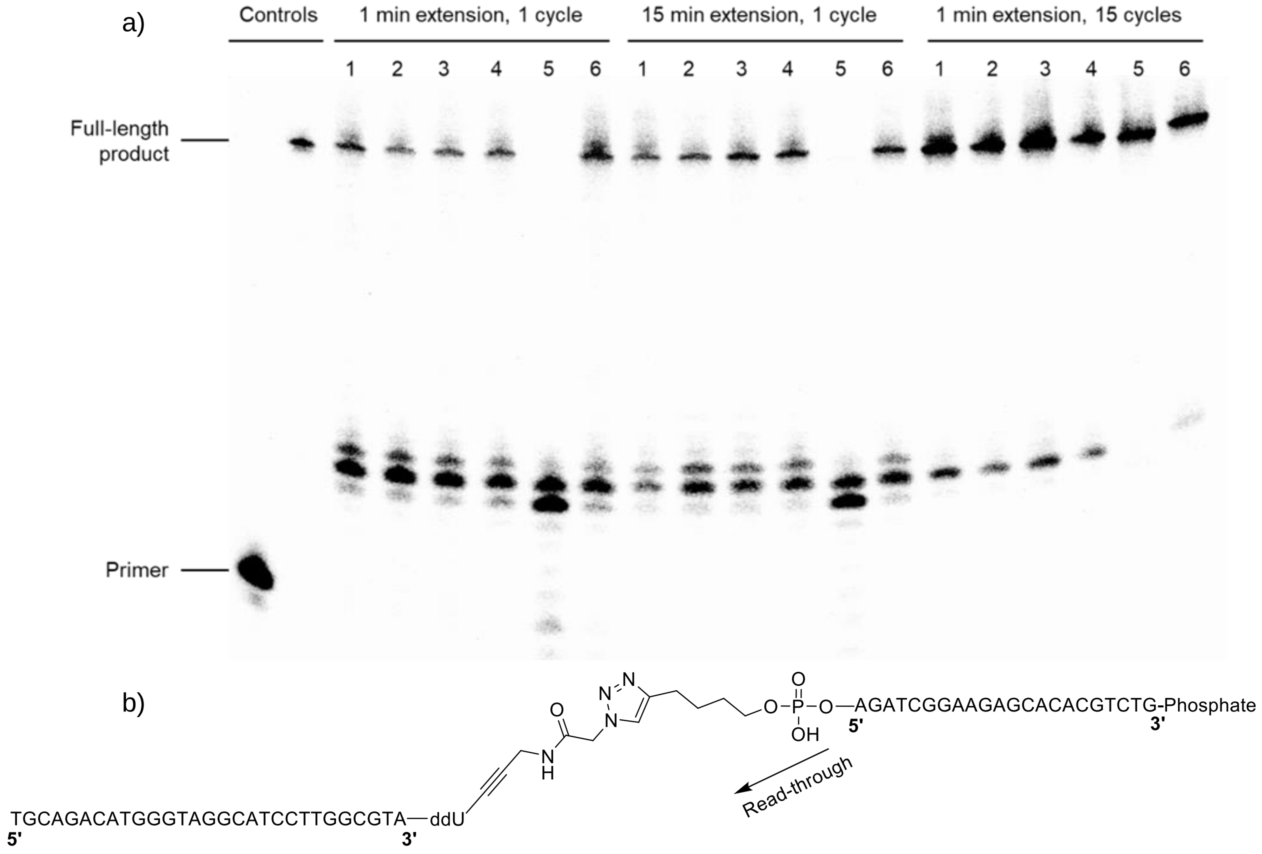

ON-dd^{ON}U

Supplementary Figure S3. a) Reading through **dd^{ON}NTP** linker. Autoradiogram showing PEX using ddU^{ON}TP-containing template and 3' – 5' exonuclease-deficient Phusion DNA polymerase (Phusion exo-) in various buffers. 1 – Phusion HF buffer, 2 – Phusion GC buffer, 3 – Phire reaction buffer, 4 – SuperFi buffer, 5 – Invitrogen Collibri Library Amplification Master Mix (with active Platinum SuperFi DNA polymerase), 6 – Invitrogen Collibri Library Amplification Master Mix treated with thermolabile proteinase K before the addition of Phusion exo-; b) Structure and sequence of read-through product **ON-dd^{ON}U**.

3. Experimental Procedures

5-Iodo-2',3'-dideoxycytidine 5'-triphosphate (dd^{I}CTP)

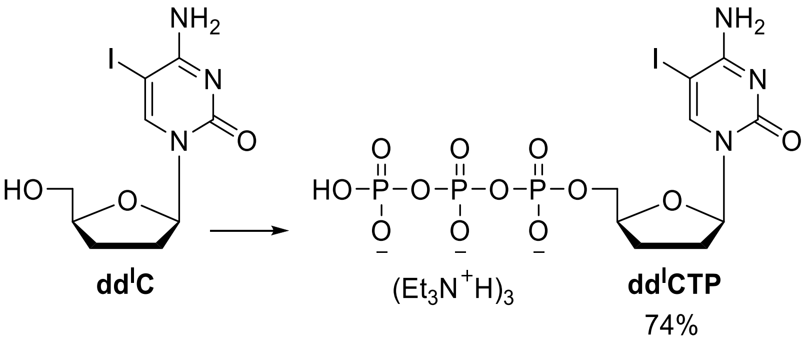

5-Iodo-2',3'-dideoxycytidine (**dd^{I}C**) (1 g, 2.97 mmol) was suspended in ACN (29.7 ml) under argon atmosphere, Bu_3N (0.734 ml, 1.04 eq.) was added. The suspension was stirred for 30 min at room temperature, then cooled to -15 – -10 ^{o}C, followed by the dropwise addition of diphosphoryl chloride (1.21 ml, 3 eq). After 20 min tributylammonium pyrophosphate (TBAPP) cocktail consisting of: 0.5 M TBAPP (23.8 ml, 4 eq.) solution in ACN, NBu_3 (4.94 ml, 7 eq.) and ACN (10 ml) was added to the reaction mixture and stirred for 30 min at room temperature. The reaction mixture was quenched with cooled 1 M triethylammonium bicarbonate (TEAB) buffer (100 ml, pH 7). The product was purified by ion exchange

chromatography on Q Sepharose FF resin applying water/1 M TEAB (5-42 %) gradient. The desired triphosphate was obtained in 74 % (2.2 mmol) yield. UV: λmax = 294 nm, ε = 5700 l·mol-1·cm-1. ^{1}H NMR (400 MHz, D_2O) δ 7.99 (s, 1H, H-6), 5.81 (dd, *J* = 6.7, 3.5 Hz, 1H, H-1'), 4.22 – 4.13 (m, 1H, H-4'), 4.08 (ddd, *J* = 11.4, 5.9, 2.7 Hz, 1H, H-5'a), 3.93 (dt, *J* = 11.8, 6.0 Hz, 1H, H-5'b), 2.34 – 2.15 (m, 1H, H-2'a), 1.97 – 1.81 (m, 2H, H-2'b and H-3'a), 1.81 – 1.71 (m, 1H, H-3'b). ^{13}C NMR (101 MHz, D_2O) δ 164.37 (C-4), 156.33 (C=O-2), 147.56 (C-6), 87.18 (CH-1'), 80.60 (d, *J* = 8.5 Hz, CH-4'), 66.92 (d, J = 5.6 Hz, CH-5'), 57.37 (C-5), 31.84 (CH_2-2'), 24.76 (CH_2-3'). ^{31}P NMR (162 MHz, D_2O) δ -10.21 (d, *J* = 20.3 Hz, P_γ), -11.60 (d, *J* = 20.0 Hz, P_α), -23.47 (t, *J* = 20.2 Hz, P_β). HRMS (ESI$^-$) m/z: [M-H]$^-$ calcd for $C_9H_{14}IN_3O_{12}P_3$ 575.8835; found 575.8828.

5-(3-aminoprop-1-ynyl)-2',3'-dideoxycytidine 5'-triphosphate (dd^{PA}CTP)

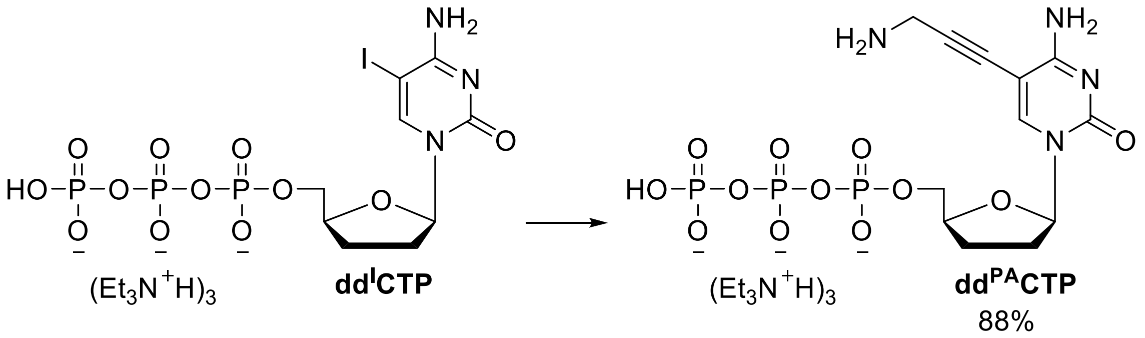

Water-acetonitrile mixture 2:1 (6.6 ml) was added through septum to an argon flushed flask containing 5-iodo-2',3'-dideoxycytidine 5'-triphosphate (**dd^{I}CTP**) (1.64 mmol), followed by the addition of propargylamine (209 ul, 2 eq.) and CuI (0.032 g, 0.1 mmol, 10 mol%). In a separate flask under argon atmosphere Pd(OAc)$_2$ (18.7 mg, 0.05 mmol, 5 mol%) and TPPTS (0.234 g, 0.25 mmol, 5 eq. to Pd) were combined, water-acetonitrile mixture 2:1 (5 ml) was then added and stirred till full dissolution of solids. Prepared catalyst solution was applied to the reaction mixture and stirred for 1 h at 40 ^{o}C. The product was purified by ion exchange chromatography on Q Sepharose FF resin applying water/1M TEAB gradient (5-52 %). Desalting was carried out on C18 column with water/acetonitrile gradient (0-100 %). The product was obtained in 88 % (1.45 mmol) yield. UV: λmax = 294 nm, ε = 9300 l·mol^{-1}·cm^{-1}. ^{1}H NMR (400 MHz, D_2O) δ 8.31 (s, 1H, H-6), 5.88 (d, *J* = 5.9 Hz, 1H, H-1'), 4.32 – 4.21 (m, 2H, H-4' and H-5'a), 4.11 – 4.01 (m, 1H, H-5'b), 3.96 (s, 2H, H-9), 2.44 – 2.29 (m, 1H, H-2'a), 2.05 – 1.83 (m, 3H, H-2'b and H-3'a,b). ^{13}C NMR (101 MHz, D_2O) δ 164.17 (C-4), 155.25 (C=O-2), 146.27 (CH-6), 87.49 (C-8), 87.02 (CH-1'), 81.36 (d, *J* = 7.2 Hz, CH-4'), 77.57 (C-7), 65.95 (CH_2-5'), 32.72 (CH_2-2'), 29.82 (CH_2-9), 23.52 (CH_2-3'). ^{31}P NMR (162 MHz, D_2O) δ -10.59 (br s, P_γ), -11.52 (br s, P_α), -23.17 (br s, P_β). HRMS (ESI$^-$) m/z: [M-H]$^-$ calcd for $C_{12}H_{18}N_4O_{12}P_3$ 503.0134; found 503.0233.

5-(3-(2-azidoacetamido)prop-1-ynyl)-2',3'-dideoxyuridine 5'-triphosphate (dd^{N3}CTP)

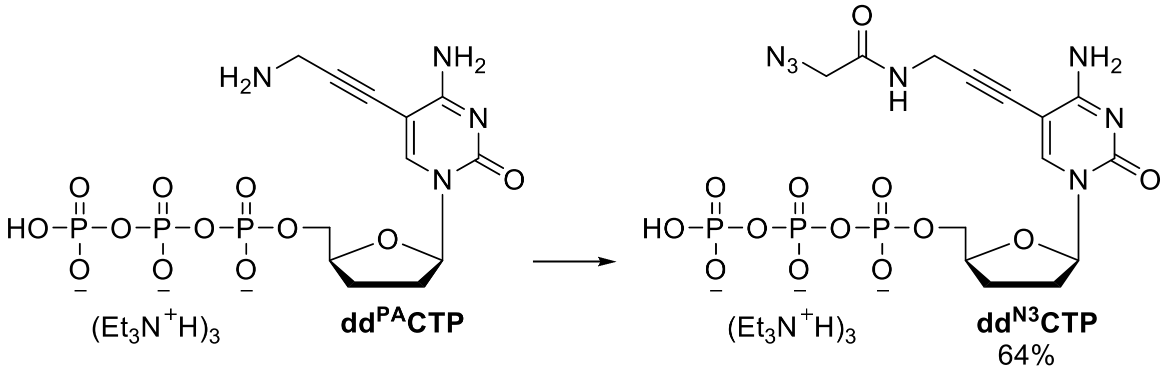

5-PA-2',3'-dideoxycytidine 5'-triphosphate (**dd^{PA}CTP)** (0.031 mmol) was dissolved in Na_2CO_3/$NaHCO_3$ 1:9 buffer (1 ml, 0.1 M, pH 9) and DMF (0.2 ml). Solution of azidoacetic acid NHS ester (0.018 g, 0.092 mmol, 3 eq.) in DMF (0.4 ml) was then added. After stirring for 30 min at room temperature, reaction mixture was purified by ion exchange chromatography on Q Sepharose FF resin applying water/1 M TEAB gradient (5-52 %). Desalting was carried out on C18 column with water/acetonitrile gradient (0-100 %). Product was obtained 64 % (0.020 mmol) yield. UV: λmax = 294 nm, ε = 9300 ml·mol^{-1}·cm^{-1}. ^{1}H NMR (400 MHz, D_2O) δ 8.11 (s, 1H, H-6), 5.93 (dd, *J* = 6.3, 2.2 Hz, 1H, H-1'), 4.33 – 4.26 (m, 1H, H-4'), 4.25 –

4.19 (m, 1H, H-5'a), 4.17 (s, 2H, H-9), 4.12 – 4.02 (m, 1H, H-5'b), 3.99 (s, 2H, H-12), 2.45 – 2.31 (m, 1H, H-2'a), 2.06 – 1.93 (m, 2H, H-2'b and H-3'a), 1.88 – 1.75 (m, 1H, H-3'b). ^{13}C NMR (101 MHz, D_2O) δ 170.14 (C=O-11), 164.02 (C-4), 154.53 (C=O-2), 145.24 (CH-6), 91.64 (C-5 and C8), 87.42 (CH-1'), 80.98 (d, *J* = 7.6 Hz, CH-4'), 73.03 (C-7), 66.66 (d, *J* = 3.5 Hz, CH_2-5'), 51.68 (CH_2-12), 32.10 (CH_2-2'), 29.72 (CH_2-9), 24.46 (CH_2-3'). ^{31}P NMR (162 MHz, D_2O) δ -11.12 (d, *J* = 15.7 Hz, P_γ), -11.50 (d, *J* = 18.9 Hz, P_α), -23.57 (br s, P_β). HRMS (ESI$^-$) m/z: [M-H]$^-$ calcd for $C_{14}H_{19}N_7O_{13}P_3$ 586.0254; found 586.0251.

5-Iodo-2',3'-dideoxyuridine 5'-triphosphate (dd^{I}UTP)

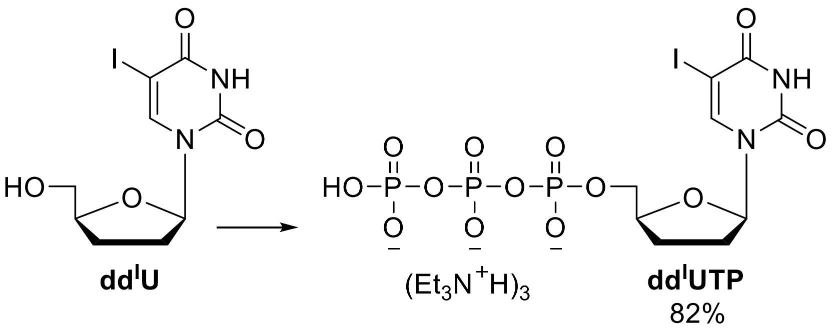

NBu_3 (1.0 ml, 1.5 eq.) was added to the solution of 5-iodo-2',3'-dideoxyuridine (**dd^{I}U**) (1 g, 2.96 mmol) in trimethyl phosphate (TMP) (14 ml) under argon atmosphere. The suspension was stirred for 30 min at room temperature, then cooled in an ice bath (0 – 5 ºC), followed by the dropwise addition of phosphorus oxychloride (0.79 ml, 3 eq.). After 2 h TBAPP cocktail consisting of: 0.5 M TBAPP (22.52 ml, 4 eq.) solution in ACN, Bu_3N (4.67 ml, 7 eq) and ACN (10 ml) was added to the reaction mixture and stirred for 30 min at room temperature. The reaction mixture was then quenched with cooled 0.5 M TEAB buffer (100 ml, pH 7). The product was purified by ion exchange chromatography on Q Sepharose FF resin applying water/1 M TEAB gradient (5-56 %). The desired triphosphate was obtained in 82 % (2.44 mmol) yield. UV: λmax = 287 nm, ε = 7700 l·mol^{-1}·cm^{-1}. ^{1}H NMR (400 MHz, D_2O) δ 8.12 (s, 1H, H-6), 5.94 (dd, *J* = 6.8, 3.7 Hz, 1H, H-1'), 4.31 – 4.23 (m, 1H, H-4'), 4.15 (ddd, *J* = 11.1, 5.8, 2.6 Hz, 1H, H-5'a), 4.01 (dt, *J* = 11.8, 6.0 Hz, 1H, H-5'b), 2.42 – 2.27 (m, 1H, H-2'a), 2.12 – 1.97 (m, 2H, H-2'b and H-3'a), 1.95 – 1.87 (m, 1H, H-3'b). ^{13}C NMR (101 MHz, D_2O) δ 162.82 (C=O-4), 151.19 (C=O-2), 146.24 (CH-6), 86.75 (CH-1'), 80.43 (d, *J* = 8.5 Hz, CH-4'), 67.83 (C-5), 67.07 (d, *J* = 5.6 Hz, CH_2-5'), 31.12 (CH_2-2'), 25.01 (CH_2-3'). ^{31}P NMR (162 MHz, D_2O) δ -11.18 (d, *J* = 20.1 Hz, P_γ), -11.64 (d, *J* = 20.3 Hz, P_α), -23.64 (t, *J* = 20.1 Hz, P_β). HRMS (ESI$^-$) m/z: [M-H]$^-$ calcd for $C_9H_{13}IN_2O_{13}P_3$ 576.8675; found 576.8684.

5-(3-aminoprop-1-ynyl)-2',3'-dideoxyuridine 5'-triphosphate (dd^{PA}UTP)

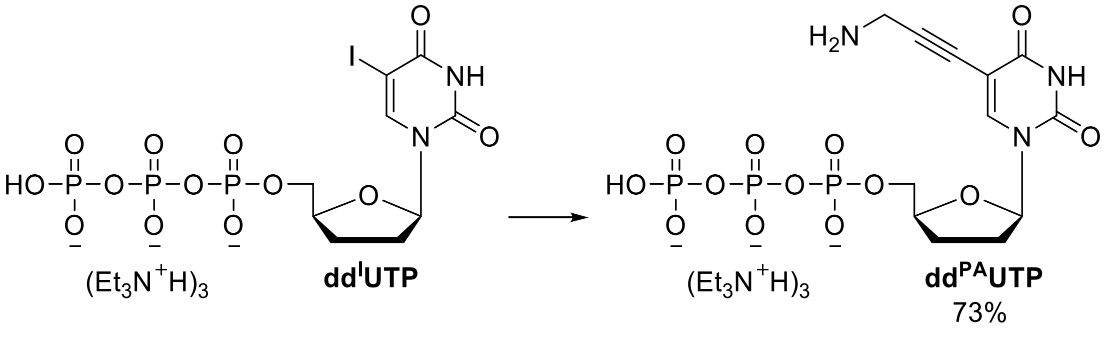

5-PA-2',3'-dideoxyuridine 5'-triphosphate (**dd^{PA}UTP**) was synthesised from 5-iodo-2',3'-dideoxyuridine 5'-triphosphate (**dd^{I}UTP**) (2.2 mmol) according to the procedure applied for preparation of **dd^{PA}CTP.** The product was purified by ion exchange chromatography on Q Sepharose FF resin applying water/1M TEAB gradient (5-46 %). Desalting was carried out on C18 column with water/acetonitrile gradient (0-100 %). The product was obtained in 73 % (1.6 mmol) yield. UV: λmax = 290 nm, ε = 13000 l·mol^{-1}·cm^{-1}. ^{1}H NMR (400 MHz, D_2O) δ 8.28 (s, 1H, H-6), 5.82 (d, *J* = 5.6 Hz, 1H, H-1'), 4.19 (br s, 2H, H-4' and H-5'a), 3.97 (br s, 1H, H-5'b), 3.83 (br s, 2H, H-9), 2.37 – 2.18 (m, 1H, H-2'a), 2.07 – 1.93 (m, 1H, H-2'b), 1.93 – 1.75 (m, 2H, H-3'). ^{13}C NMR (101 MHz, D_2O) δ 164.02 (C-4), 150.22 (C=O-2), 146.33 (CH-6), 97.15 (C-5), 87.14 (CH-1'), 84.99 (C-8), 81.49 (d, *J* = 2.2 Hz, CH-4'), 78.13 (C-7), 65.81 (CH_2-5'), 32.52 (CH_2-2'), 29.77 (CH_2-9), 23.50

(CH_2-3’). ^{31}P NMR (162 MHz, D_2O) δ -12.18 (br s, $P_γ$), -12.73 (d, *J* = 15.1 Hz, $P_α$), -24.84 (br s, $P_β$). HRMS (ESI-) m/z: [M-H]- calcd for $C_{12}H_{17}N_3O_{13}P_3$ 503.9974; found 503.9983.

5-(3-(2-azidoacetamido)prop-1-ynyl)-2’,3’-dideoxyuridine 5’-triphosphate (dd^{N3}UTP)

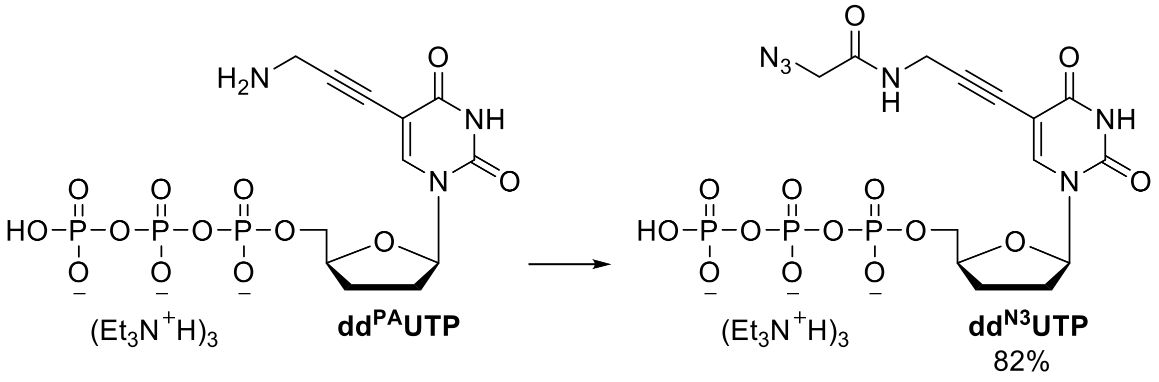

5-N3-2’,3’-dideoxyuridine 5’-triphosphate (**dd^{N3}UTP**) was synthesised from 5-PA-2’,3’-dideoxyuridine 5’-triphosphate (**dd^{PA}UTP**) (1.37 mmol) according to the procedure applied for preparation of **dd^{N3}CTP.** The product was purified by ion exchange chromatography on Q Sepharose FF resin applying water/1M TEAB gradient (5-55 %). Desalting was carried out on C18 column with water/acetonitrile gradient (0-100 %). The product was obtained in 82 % (1.13 mmol) yield. UV: λmax = 290 nm, ε = 13000 l·mol^{-1}·cm^{-1}. ^{1}H NMR (400 MHz, D_2O) δ 8.10 (s, 1H, H-6), 5.97 (dd, *J* = 6.8, 3.2 Hz, 1H, H-1’), 4.30 (br s, 1H, H-4’), 4.25 – 4.18 (m, 1H, H-5’a), 4.15 (s, 2H, H-9), 4.11 – 4.02 (m, 1H, H-5’b), 3.99 (s, 2H, H-12), 2.47 – 2.30 (m, 1H, H-2’a), 2.13 – 1.97 (m, 2H, H-2’b and H-3’a), 1.97 – 1.81 (m, 1H, H-3’b). ^{13}C NMR (101 MHz, D_2O) δ 170.01 (C=O-11), 164.25 (C=O-4), 150.31 (C=O-2), 145.03 (CH-6), 98.33 (C-5), 89.29 (C-8), 86.89 (CH-1’), 80.83 (d, *J* = 7.9 Hz, CH-4’), 73.83 (C-7), 66.79 (d, *J* = 3.2 Hz, CH_2-5’), 51.70 (CH_2-12), 31.45 (CH_2-2’), 29.64 (CH_2-9), 24.72 (CH_2-3’). ^{31}P NMR (162 MHz, D_2O) δ -11.10 (d, *J* = 10.2 Hz, $P_γ$), -11.47 (d, J = 15.5 Hz, $P_α$), -23.53 (br s, $P_β$). HRMS (ESI-) m/z: [M-H]- calcd for $C_{14}H_{18}N_6O_{14}P_3$ 587.0094; found 587.0104.

7-Deaza-7-iodo-2’,3’-dideoxyadenosine 5’-triphosphate (dd^{I}ATP)

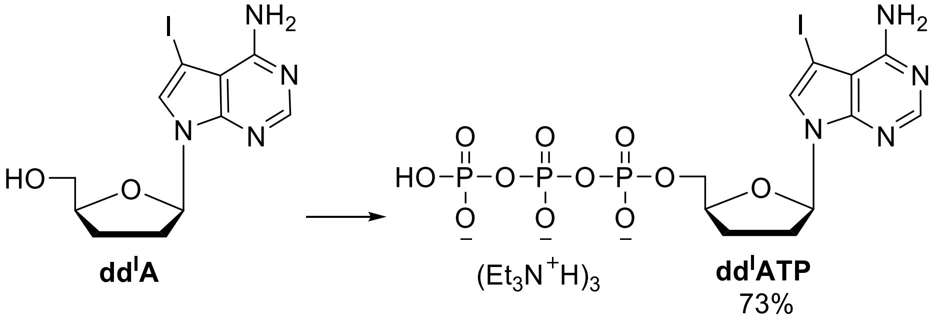

7-Deaza-7-iodo-2’,3’-dideoxyadenosine (**dd^{I}A**) (1 g, 2.78 mmol) was suspended in ACN (27.8 ml) under argon atmosphere. The suspension was stirred for 30 min at room temperature, then cooled in an ice bath. Immediately before dropwise addition of diphosphoryl chloride (1.14 ml, 3 eq.) the flask was lifted out of the ice bath. When the solid was completely dissolved (~7 min) the flask was immersed in ice bath and stirred for 10 min. TBAPP cocktail consisting of: 0.5 M TBAPP solution in ACN (22.2 ml, 4 eq.), Bu_3N (4.62 ml, 7 ekv.) and ACN (10 ml) was then added to the reaction mixture and stirred for 30 min at room temperature. The reaction mixture was quenched with cooled 1 M TEAB buffer (100 ml, pH 7). The product was purified by ion exchange chromatography on Q Sepharose FF resin applying water/1 M TEAB gradient (5-52 %). The desired triphosphate was obtained in 73 % (2.03 mmol) yield. UV: λmax = 283 nm, ε = 8500 l·mol^{-1}·cm^{-1}. ^{1}H NMR (400 MHz, D_2O) δ 7.98 (s, 1H, H-2), 7.54 – 7.39 (m, 1H, H-8), 6.23 – 6.13 (m, 1H, H-1’), 4.32 (br d, *J* = 2.7 Hz, 1H, H-4’), 4.23 – 4.09 (m, 1H, H-5’a), 4.06 – 3.90 (m, 1H, H-5’b), 2.56 – 2.39 (m, 1H, H-2’a), 2.22 – 2.05 (m, 2H, H-2’b and H-3’a), 2.04 – 1.90 (m, 1H, H-3’b). ^{13}C NMR (101 MHz, D_2O) δ 153.65 (C-6), 147.53 (C-4), 147.24 (CH-2), 128.01 (CH-8), 102.59 (C-5), 84.15 (CH-1’), 80.00 (d, *J* = 8.2 Hz, CH-4’), 67.45 (d, *J* = 5.7 Hz, CH_2-5’), 51.73 (C-7), 31.65 (CH_2-2’), 25.65 (CH_2-3’). ^{31}P NMR (162 MHz, D_2O) δ -10.78 (d, *J* = 19.6 Hz, $P_γ$), -11.34 (d, *J* = 19.9 Hz, $P_α$), -23.35 (t, *J* = 19.7 Hz, $P_β$). HRMS (ESI-) m/z: [M-H]- calcd for $C_{11}H_{15}IN_4O_{11}P_3$ 598.8995; found 598.9009.

7-Deaza-7-(3-aminoprop-1-ynyl)-2',3'-dideoxyadenosine 5'-triphosphate (dd^{PA}ATP)

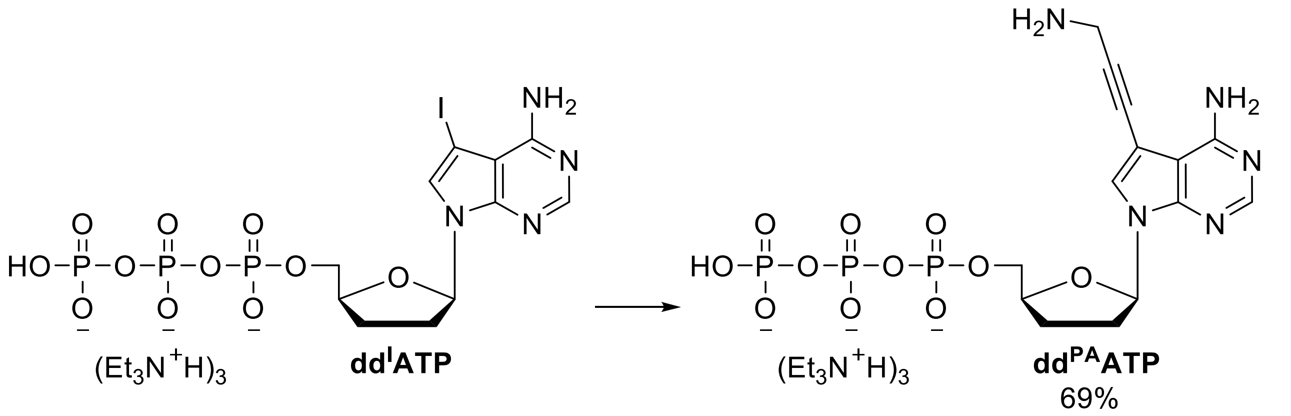

7-Deaza-7-PA-2',3'-dideoxyadenosine (**dd^{PA}ATP**) was synthesised from 7-deaza-7-iodo-2',3'-dideoxyadenosine (**dd^{I}ATP**) (2.03 mmol) according to the procedure applied for preparation of **dd^{PA}CTP.** In this reaction addition of Et_3N (0.565 ml, 4.06 mmol, 2 eq.) prior to Pd/TPPTS catalyst system was necessary. The product was purified by ion exchange chromatography on Q Sepharose FF resin applying water/1 M NaCl gradient (5-24 %). Desalting was carried out on C18 column using water/acetonitrile gradient (0-100 %). The product was obtained in 69 % (1.4 mmol) yield. UV: λmax = 280 nm, ε = 12700 l·mol^{-1}·cm^{-1}. ^{1}H NMR (400 MHz, D_2O) δ 7.43 (s, 1H, H-2), 7.18 (s, 1H, H-8), 5.83 (br s, 1H, H-1'), 4.20 (br s, 1H, H'4'), 4.06 (br s, 1H, H-5'a), 4.00 – 3.74 (m, 3H, H-12 and H-5'b), 2.23 (br d, *J* = 6.6 Hz, 1H, H-2'a), 1.97 (br s, 1H, H-3'a), 1.76 (br s, 1H, H-2'b), 1.64 (br s, 1H, H-3'b). ^{13}C NMR (101 MHz, D_2O) δ 155.01 (C-6), 150.10 (CH-2), 146.16 (C-4), 126.97 (CH-8), 101.48 (C-5), 93.97 (C-7), 84.28 (CH-1'), 83.35 (C-10), 79.96 (d, *J* = 7.4 Hz, CH-4'), 78.53 (C-9), 67.37 (d, *J* = 4.1 Hz, CH_2-5'), 31.89 (CH_2-2'), 29.81 (CH_2-11), 25.32 (CH_2-3'). ^{31}P NMR (162 MHz, D_2O) δ -6.43 (d, *J* = 18.6 Hz, P_γ), -10.57 (d, *J* = 18.0 Hz, P_α), -20.93 (t, *J* = 18.2 Hz, P_β).HRMS (ESI$^-$) m/z: [M-H]$^-$ calcd for $C_{14}H_{19}N_5O_{11}P_3$ 526.0294; found 526.0304.

7-Deaza-7-(3-(2-azidoacetamido)prop-1-ynyl)-2',3'-dideoxyadenosine 5'-triphosphate (dd^{N3}ATP)

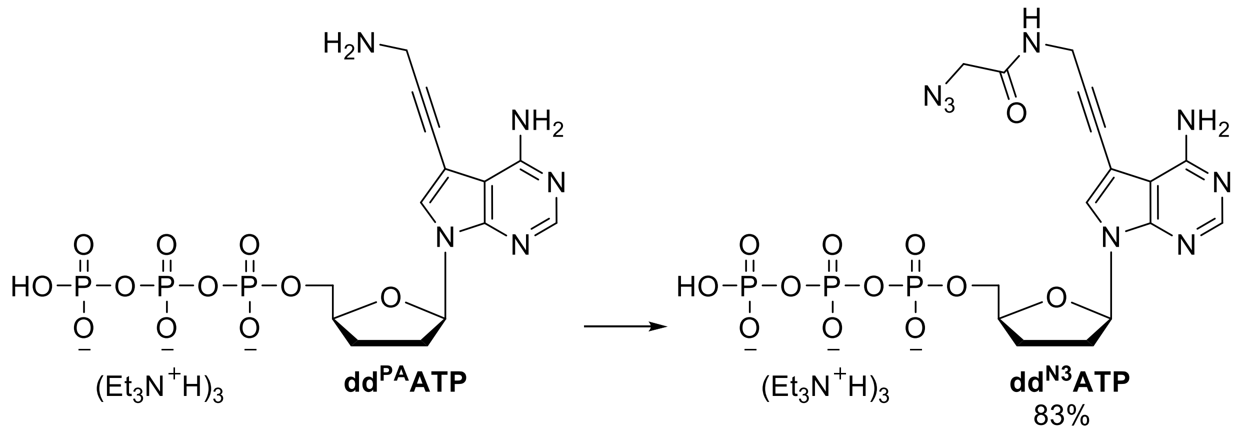

7-Deaza-7-N_3-2',3'-dideoxyadenosine (**dd^{N3}ATP**) was synthesised from 7-deaza-7-PA-2',3'-dideoxyadenosine (**dd^{PA}ATP**) (0.73 mmol) according to the procedure applied for preparation of **dd^{N3}CTP.** The product was purified by ion exchange chromatography on Q Sepharose FF resin applying water/1 M TEAB gradient (5-60 %). Desalting was carried out on C18 column using water/acetonitrile gradient (0-100 %). The product was obtained in 83 % (0.61 mmol) yield. UV: λmax = 280 nm, ε = 12700 l·mol^{-1}·cm^{-1}. ^{1}H NMR (400 MHz, D_2O) δ 7.99 (s, 1H, H-2), 7.52 (s, 1H, H-8), 6.31 (dd, *J* = 6.7, 3.9 Hz, 1H, H-1'), 4.46 – 4.36 (m, 1H, H-4'), 4.27 (s, 2H, H-12), 4.20 – 4.13 (m, 1H, H-5'a), 4.10 (s, 2H, H-15), 4.06 – 3.95 (m, 1H, H-5'b), 2.61 – 2.48 (m, 1H, H-2'a), 2.37 – 2.20 (m, 2H, H-2'b and H-3'a), 2.11 – 1.98 (m, 1H, H-3'b). ^{13}C NMR (101 MHz, D_2O) δ 170.50 (C=O-14), 156.97 (C-6), 151.87 (CH-2), 147.92 (C-4), 126.21 (CH-8), 102.77 (C-5), 95.32 (C-7), 87.60 (C-11), 84.04 (CH-1'), 79.95 (d, *J* = 8.2 Hz, CH-4'), 75.34 (C-10), 67.60 (d, *J* = 5.9 Hz, CH_2-5'), 51.90 (CH_2-15), 31.15 (CH_2-

2’), 29.89 (CH_2-12), 26.01 (CH_2-3’). ^{31}P NMR (162 MHz, D_2O) δ -6.81 (d, J = 19.7 Hz, P_γ), -10.73 (d, J = 18.9 Hz, P_α), -21.74 (t, J = 19.2 Hz, P_β). HRMS (ESI$^-$) m/z: [M-H]$^-$ calcd for $C_{14}H_{19}N_7O_{13}P_3$ 609.0414; found 609.0425.

7-Deaza-7-iodo-2’,3’-dideoxyadenosine 5’-triphosphate (dd^{I}GTP)

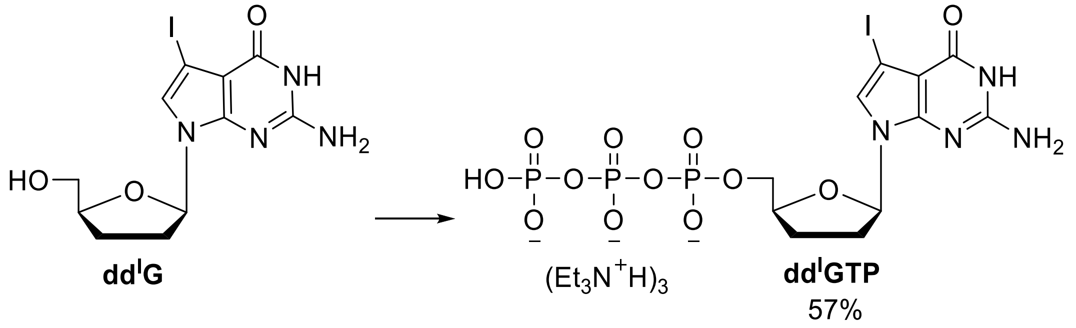

7-Deaza-7-iodo-2’,3’-dideoxyguanosine Nr. (1.13 g, 3.0 mmol) was suspended in TMP (16.8 ml) under argon atmosphere, NBu_3 (1.8 ml, 7.5 mmol, 2.5 eq.) was added. The suspension was stirred for 30 min at room temperature, then cooled in an ice bath followed by the dropwise addition of phosphorus oxychloride (0.84 ml, 3 eq.). After 1 h TBAPP cocktail consisting of: 0.5 M TBAPP solution in ACN (24 ml, 4 eq.), Bu3N (4.67 ml, 7 ekv.) and ACN (10 ml) was added to the reaction mixture and stirred for 30 min at room temperature. The reaction mixture was quenched with cooled 0.5 M TEAB buffer (100 ml, pH 7). The product was purified by ion exchange chromatography on Q Sepharose FF resin using water/1M TEAB gradient (5-75 %). The desired triphosphate was obtained in 57 % (1.71 mmol) yield. UV: λmax = 267 nm, ε = 11000 l·mol^{-1}·cm^{-1}. ^{1}H NMR (400 MHz, D_2O) δ 6.97 (s, 1H, H-8), 5.96 (dd, J = 6.7, 3.9 Hz, 1H, H-1’), 4.27 – 4.17 (m, 1H, H-4’), 4.09 – 3.98 (m, 1H, H-5’a), 3.90 – 3.81 (m, 1H, H-5’b), 2.43 – 2.26 (m, 1H, H-2’a), 2.19 – 2.04 (m, 2H, H-2’b and H-3’a), 1.98 – 1.84 (m, 1H, H-3’b). ^{13}C NMR (101 MHz, D_2O) δ 159.98 (C-6), 152.40 (C-2), 150.35 (C-4), 123.27 (CH-8), 100.40 (C-5), 83.62 (CH-1’), 79.39 (d, J = 8.3 Hz, CH-4’), 67.91 (d, J = 5.8 Hz, CH_2-5’), 54.05 (C-7), 30.81 (CH_2-2’), 26.18 (CH_2-3’). ^{31}P NMR (162 MHz, D_2O) δ -11.13 (d, J = 19.8 Hz, P_γ), -11.47 (d, J = 20.1 Hz, P_α), -23.48 (t, J = 20.0 Hz, P_β). HRMS (ESI$^-$) m/z: [M-H]$^-$ calcd for $C_{11}H_{15}IN_4O_{12}P_3$ 614.8944; found 614.8952.

7-Deaza-7-(3-aminoprop-1-ynyl)-2’,3’-dideoxyadenosine 5’-triphosphate (dd^{PA}GTP)

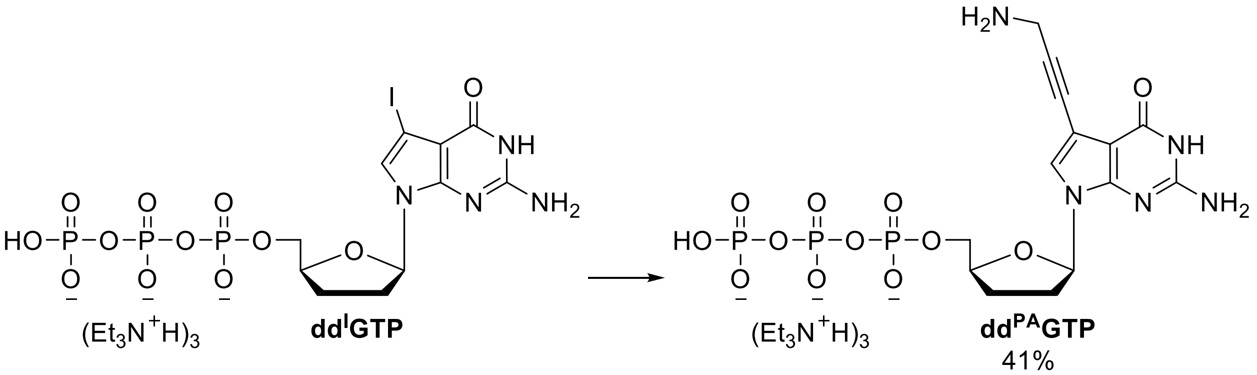

7-Deaza-7-PA-2’,3’-dideoxyguanosine (**dd^{PA}GTP**) was synthesised from 7-deaza-7-iodo-2’,3’-dideoxyguanosine (**dd^{I}GTP**) (0.2 mmol) according to the procedure applied for preparation of **dd^{PA}CTP.** The product was purified by ion exchange chromatography on Q Sepharose FF resin applying water/1M TEAB gradient (5-62 %). Desalting was carried out on C18 column using water/acetonitrile gradient (0-100 %). The product was obtained in 41 % (0.08 mmol) yield. UV: λmax = 272 nm, ε = 11900 l·mol^{-1}·cm^{-1}. ^{1}H NMR (400 MHz, D_2O) δ 7.24 (s, 1H, H-8), 5.96 (dd, J = 6.6, 3.6 Hz, 1H, H-1’), 4.32 – 4.25 (m, 1H, H-4’), 4.17 – 4.09 (m, 1H, H-5’a), 4.02 – 3.94 (m, 3H, H-12 and H-5’b), 2.43 – 2.31 (m, 1H, H-2’), 2.15 – 2.04 (m, 2H, H-2’ and H-3’a), 2.00 – 1.89 (m, 1H, H-3’). ^{13}C NMR (101 MHz, D_2O) δ 159.75 (C=O-6), 152.77 (C-2), 149.33 (C-4), 124.50 (CH-8), 99.59 (C-5), 96.94 (C-7), 84.21 (CH-1’), 81.61 (C-11), 80.02 (C-10), 79.81 (d, J = 8.5 Hz, CH-4’), 67.43 (d, J = 5.6 Hz, CH_2-5’), 31.56 (CH_2-2’), 30.19 (CH_2-12), 25.61 (CH_2-3’). ^{31}P NMR (162 MHz, D_2O) δ -10.99 (d, J = 19.3 Hz, P_γ), -11.30 (d, J = 19.7 Hz, P_α), -23.34 (t, J = 18.7 Hz, P_β). HRMS (ESI$^-$) m/z: [M-H]$^-$ calcd for $C_{14}H_{19}N_5O_{12}P_3$ 542.0243; found 542.0251.

7-Deaza-7-(3-(2-azidoacetamido)prop-1-ynyl)-2',3'-dideoxyadenosine 5'-triphosphate (dd^{N3}GTP)

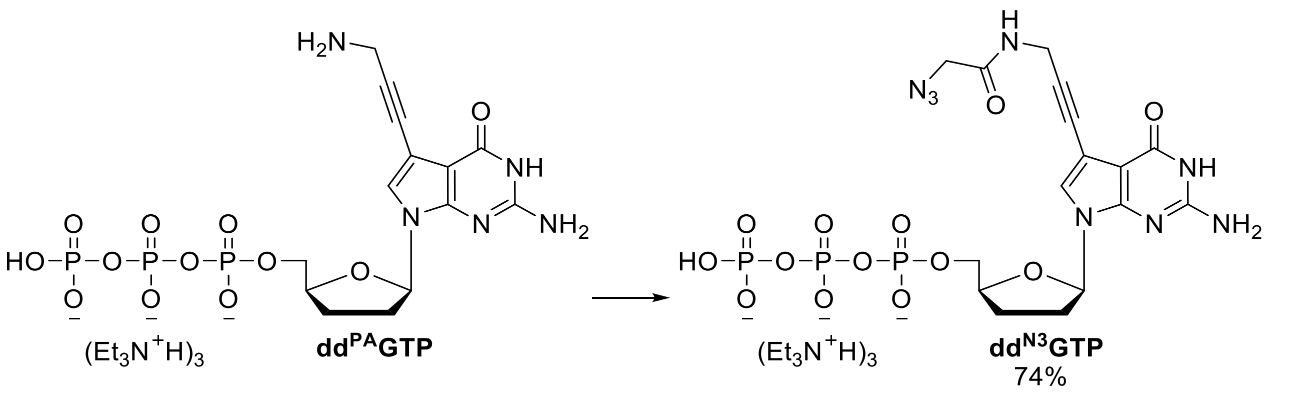

7-Deaza-7-N_3-2',3'-dideoxyguanosine (**dd^{N3}GTP**) was synthesised from 7-deaza-7-PA-2',3'-dideoxyguanosine (**dd^{PA}GTP**) (0.04 mmol) according to the procedure applied for preparation of **dd^{N3}CTP.** The product was purified by ion exchange chromatography on Q Sepharose FF resin applying water/1M TEAB gradient (5-70%). Desalting was carried out on C18 column using water/acetonitrile gradient (0-100%). The product was obtained in 74 % (0.03 mmol) yield. UV: λmax = 272 nm, ε = 11900 $l\cdot mol^{-1}\cdot cm^{-1}$. ^{1}H NMR (400 MHz, D_2O) δ 7.21 (s, 1H, H-8), 6.09 (dd, *J* = 6.7, 4.1 Hz, 1H, H-1'), 4.37 – 4.29 (m, 1H, H-4'), 4.23 (s, 2H, H-15), 4.18 – 4.10 (m, 1H, H-5'a), 4.05 (s, 2H, H-12), 4.01 – 3.91 (m, 1H, H-5'b), 2.51 – 2.38 (m, 1H, H-2'a), 2.29 – 2.14 (m, 2H, H-2'b and H-3'a), 2.07 – 1.95 (m, 1H, H-3'b). ^{13}C NMR (101 MHz, D_2O) δ 170.07 (C=O-14), 160.15 (C=O-6), 152.92 (C-2), 149.87 (C-4), 123.71 (CH-8), 99.74 (C-5), 98.07 (C-7), 85.86 (CH-11), 83.88 (CH-1'), 79.65 (d, *J* = 8.3 Hz, CH-4'), 75.97 (C-10), 67.79 (d, *J* = 5.6 Hz, CH_2-5'), 51.84 (CH_2-15), 30.87 (CH_2-2'), 29.88 (CH_2-12), 26.04 (CH_2-3'). ^{31}P NMR (162 MHz, D^2O) δ -11.09 (br s, P_γ and P_α), -23.33 (P_β). HRMS (ESI^-) m/z: $[M-H]^-$ calcd for $C_{16}H_{20}N_8O_{13}P_3$ 625.0363; found 625.0376.

General procedure for CuAAC click reaction

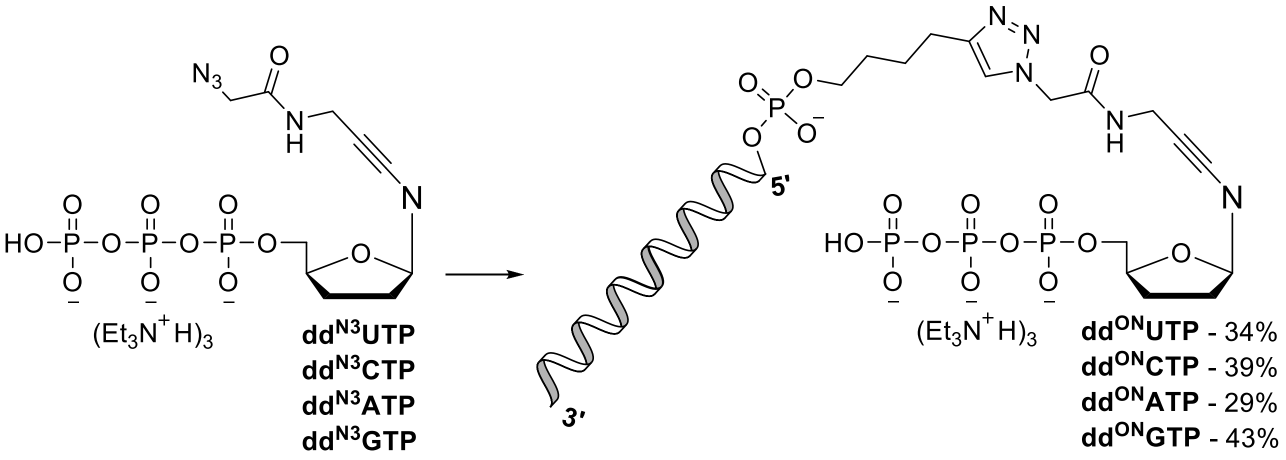

All reaction components were applied to the reaction mixture as solutions in water unless specified differently. Corresponding **dd^{N3}NTP** (3 eq., 2-4 mM) solution was added to alxyl-oligonucleotide (5'-hexynyl-AGATCGGAAGAGCACACGTCTG-3'-biotin; full structure in provided in Supplementary Figure S2) (100-210 nmol) solution in sodium phosphate buffer (1 ml, 100 mM, pH 7). In a separate vial $CuSO_4$ (100 mM, 12 eq.) and THPTA (250 mM, 5 eq. to $CuSO_4$) were premixed and added to the reaction mixture, followed by the addition of sodium ascorbate (1 M, 50 eq.to $CuSO_4$). Reaction mixture was stirred for 20 min at 42 ^{o}C, quenched with 0.5 M EDTA-Na_2 solution (0.5 ml, pH 8). The products were purified by semi-preparative HPLC applying reverse-phase chromatography (YMC-Actus Triart C18 column). **dd^{ON}CTP** and **dd^{ON}UTP** were purified using 100 mM TEAAc/ACN (11-18%) eluent system for linear gradient

formation. While **dd^{ON}ATP** and **dd^{ON}GTP** were purified using 100 mM TEAAc/ACN (10-20%) eluent system for linear gradient formation. **dd^{ON}NTPs** were desalted using water/ACN (5-100 %) gradient.

dd^{ON}CTP was obtained – 39% (82 nmol) yield, 98% purity. HRMS (ESI$^-$): calculated monoisotopic mass for [M]: 7916.345; found: 7916.342. **dd^{ON}UTP** was obtained – 34% (67 nmol) yield, 98% purity. HRMS (ESI$^-$): calculated monoisotopic mass for [M]: 7917.329; found: 7917.320. **dd^{ON}ATP** was obtained – 29% (29 nmol) yield, 94% purity. HRMS (ESI$^-$): calculated monoisotopic mass for [M]: 7939.352; found: 7939.371. **dd^{ON}GTP** was obtained – 43 % (90 nmol) yield, 96% purity. HRMS (ESI$^-$): calculated monoisotopic mass for [M]: 7955.352; found: 7955.349.

3.1. Preparation of dd^{ON}UTP for read-through assay

dd^{ON}UTP product for read-through efficiency measurements was synthesized according to general procedure for CuAAC click reaction above using oligonucleotide with sequence: 5'-hexynyl-AGATCGGAAGAGCACACGTCTG-3'-phosphate. The product was purified by semi-preparative HPLC applying reverse-phase chromatography (YMC-Actus Triart C18 column) using 100 mM TEAAc/ACN (10-15 %) eluent system for linear gradient formation and desalted using water/ACN (5-100%) gradient. Product was obtained in 29% yield, 97% purity. HRMS (ESI-): calculated monoisotopic mass for [M]: 7618.203; found: 76185.192.

3.2. Purification of ON-dd^{ON}U – read-through product

ON-dd^{ON}U purification was performed applying reverse-phase chromatography (YMC-Actus Triart C18 column) using 100 mM TEAAc/ACN (10-16%) eluent system for linear gradient formation and desalted using water/ACN (5-100%) gradient via analytical HPLC system (final product structure is provided in Scheme S3). HRMS (ESI$^-$): calculated monoisotopic mass for [M]: 16409.752; found: 16409.787.

3.3. HPLC and mass spectra of dd^{ON}NTPs and ON-dd^{ON}U

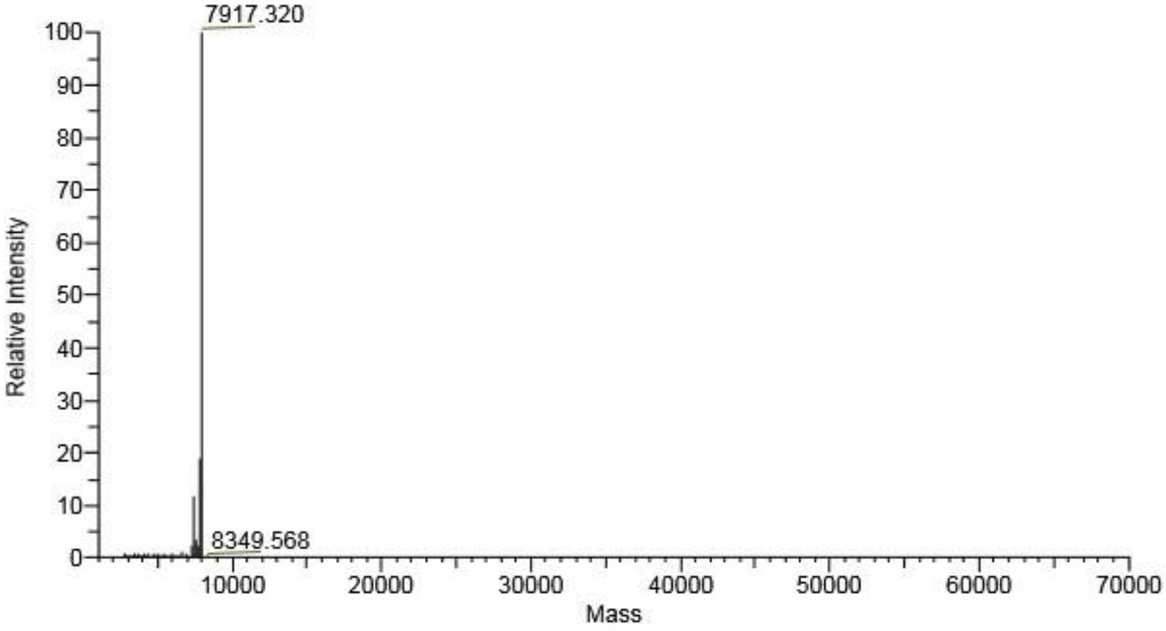

Supplementary Figure S4. Deconvoluted MS spectrum of **dd^{ON}UTP.** HRMS (ESI$^-$): calculated monoisotopic mass for [M]: 7917.329; found: 7917.320.

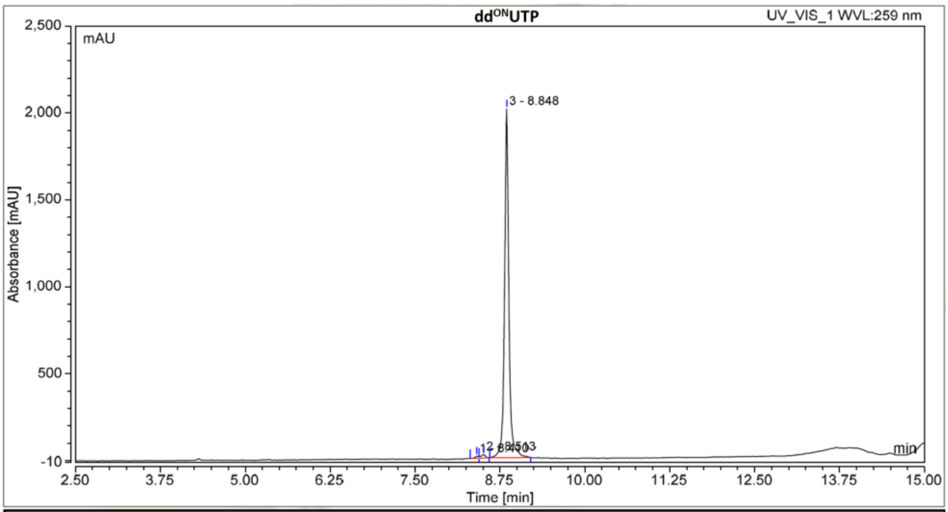

Integration Results

No.	Peak Name	Retention Time min	Area mAU*min	Height mAU	Relative Area %	Relative Height %	Amount n.a.
1		8.410	0.734	12.738	0.49	0.62	n.a.
2		8.513	1.410	20.132	0.95	0.99	n.a.
3		8.848	146.565	2006.390	98.56	98.39	n.a.
**Total:**			**148.708**	**2039.259**	**100.00**	**100.00**	

Supplementary Figure S5. HPLC chromatogram of **dd^{ON}UTP.**

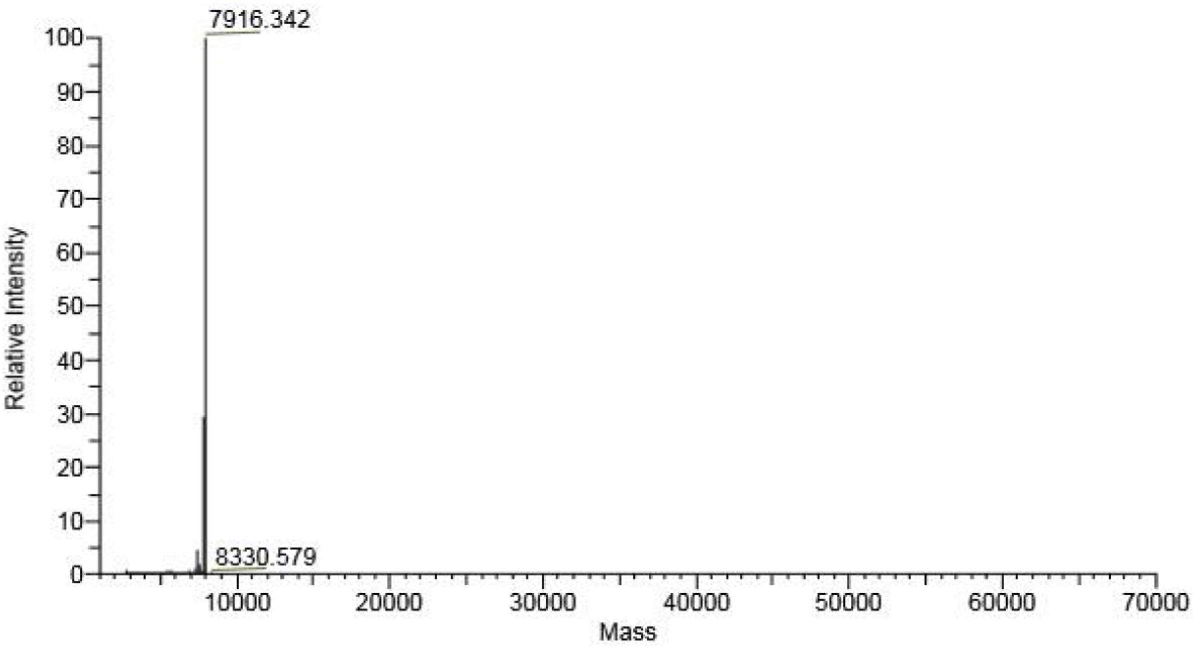

Supplementary Figure S6. Deconvoluted MS spectrum of **dd^{ON}CTP.** HRMS (ESI$^-$): calculated monoisotopic mass for [M]: 7916.345; found: 7916.342.

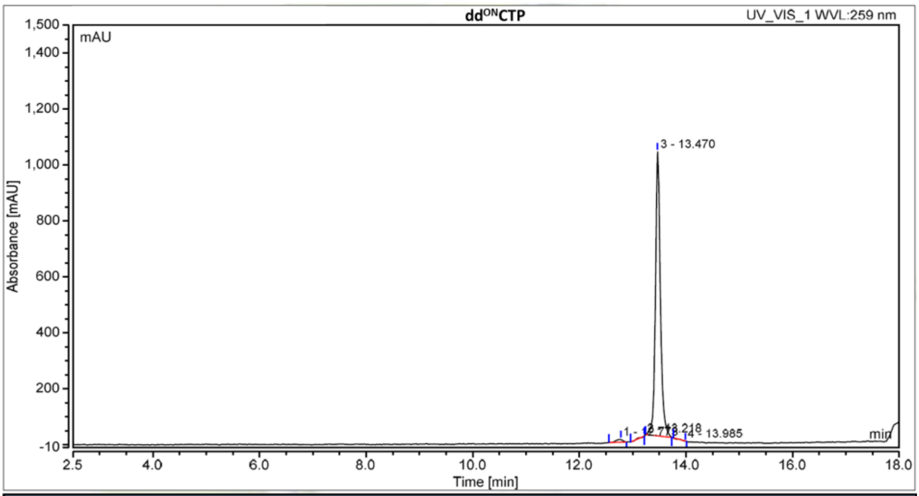

Integration Results

No.	Peak Name	Retention Time min	Area mAU*min	Height mAU	Relative Area %	Relative Height %	Amount n.a.
1		12.773	1.510	10.992	1.35	1.07	n.a.
2		13.218	0.059	0.000	0.05	0.00	n.a.
3		13.470	109.772	1015.464	98.45	98.74	n.a.
4		13.985	0.158	2.004	0.14	0.19	n.a.
**Total:**			**111.499**	**1028.459**	**100.00**	**100.00**	

Supplementary Figure S7. HPLC chromatogram of **dd^{ON}CTP.**

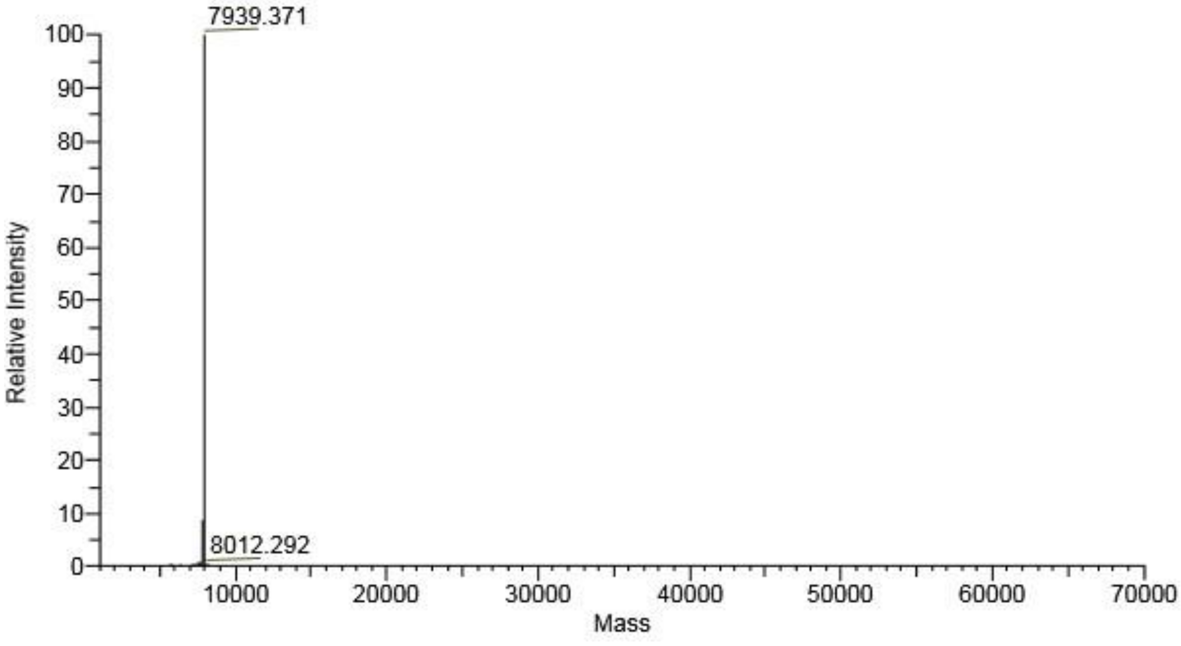

Supplementary Figure S8. Deconvoluted MS spectrum of **dd^{ON}ATP.** HRMS (ESI^{-}): calculated monoisotopic mass for [M]: 7939.352; found: 7939.371.

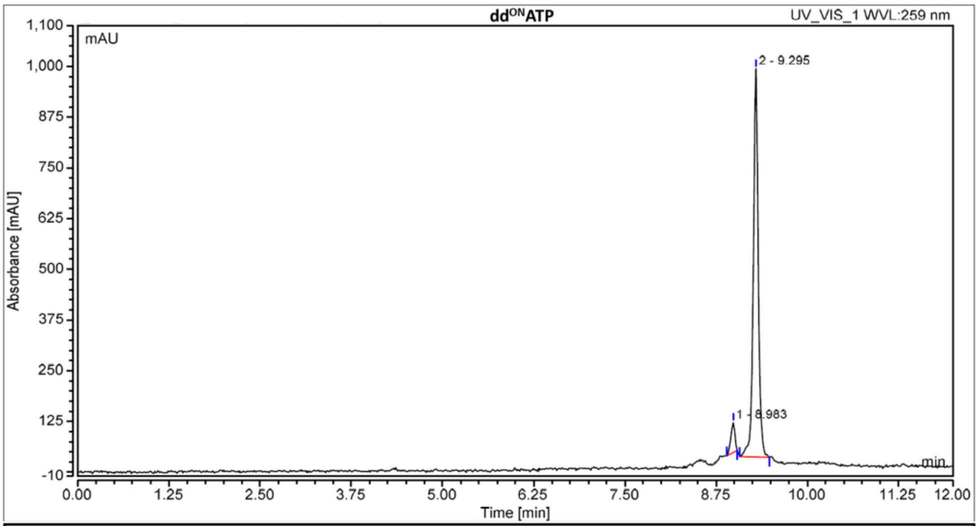

Integration Results

No.	Peak Name	Retention Time min	Area mAU*min	Height mAU	Relative Area %	Relative Height %	Amount n.a.
1		8.983	4.762	73.968	6.01	7.17	n.a.
2		9.295	74.521	957.426	93.99	92.83	n.a.
**Total:**			**79.284**	**1031.394**	**100.00**	**100.00**	

Supplementary Figure S9. HPLC chromatogram of **dd^{ON}ATP.**

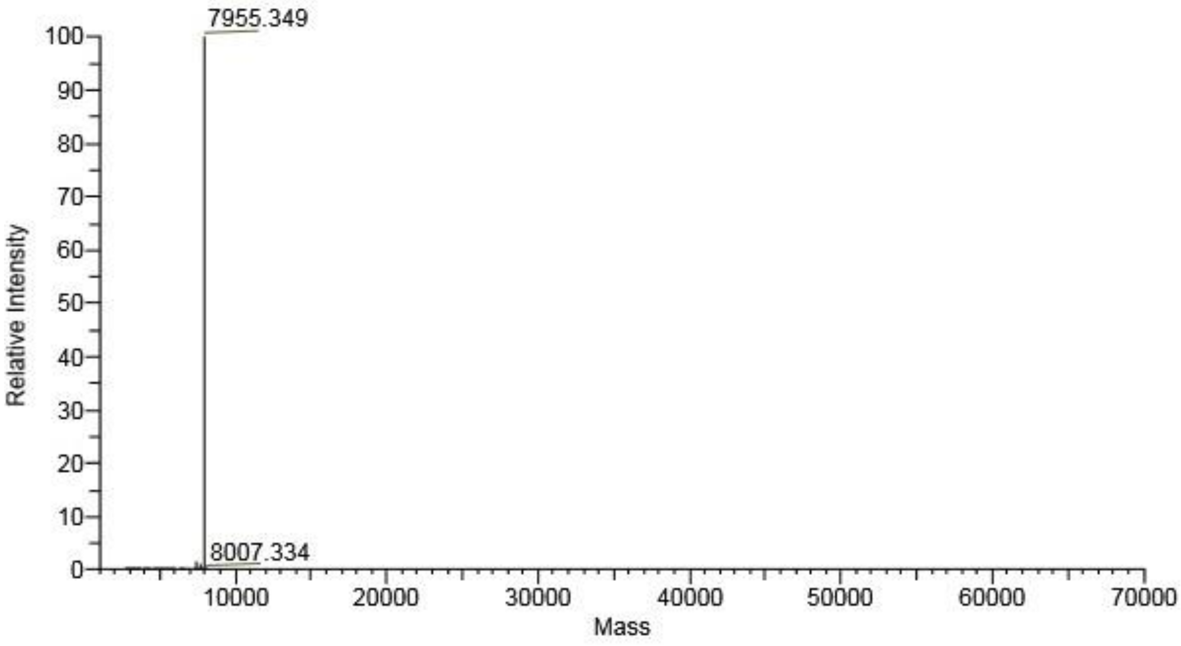

Supplementary Figure S10. Deconvoluted MS spectrum of **dd^{ON}GTP.** HRMS (ESI^{-}): calculated monoisotopic mass for [M]: 7955.352; found: 7955.349.

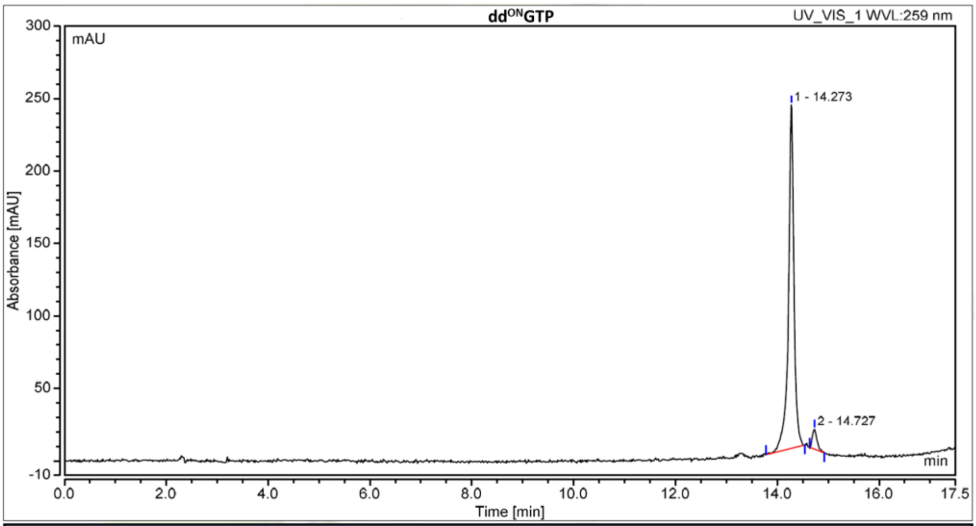

Integration Results

No.	Peak Name	Retention Time min	Area mAU*min	Height mAU	Relative Area %	Relative Height %	Amount n.a.
1		14.273	29.581	237.058	95.54	94.52	n.a.
2		14.727	1.380	13.754	4.46	5.48	n.a.
**Total:**			**30.961**	**250.813**	**100.00**	**100.00**	

Supplementary Figure S11. HPLC chromatogram of **dd^{ON}GTP.**

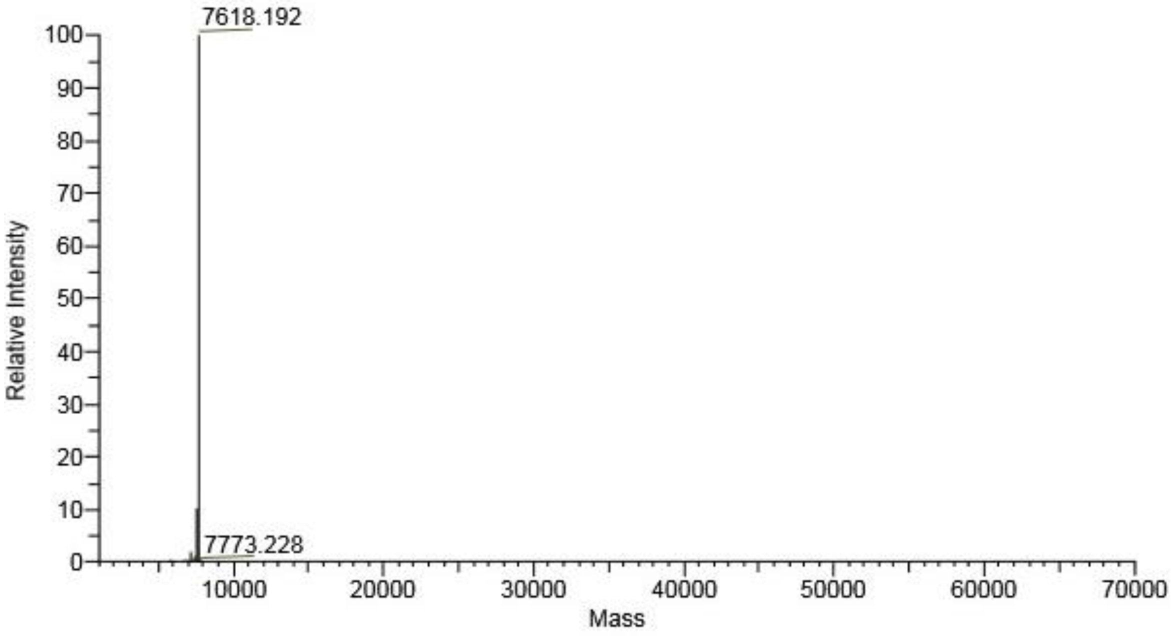

Supplementary Figure S12. Deconvoluted MS spectrum of **dd^{ON}UTP** for read-through assay**.** HRMS (ESI$^-$): calculated monoisotopic mass for [M]: 7618.203; found: 7618.192.

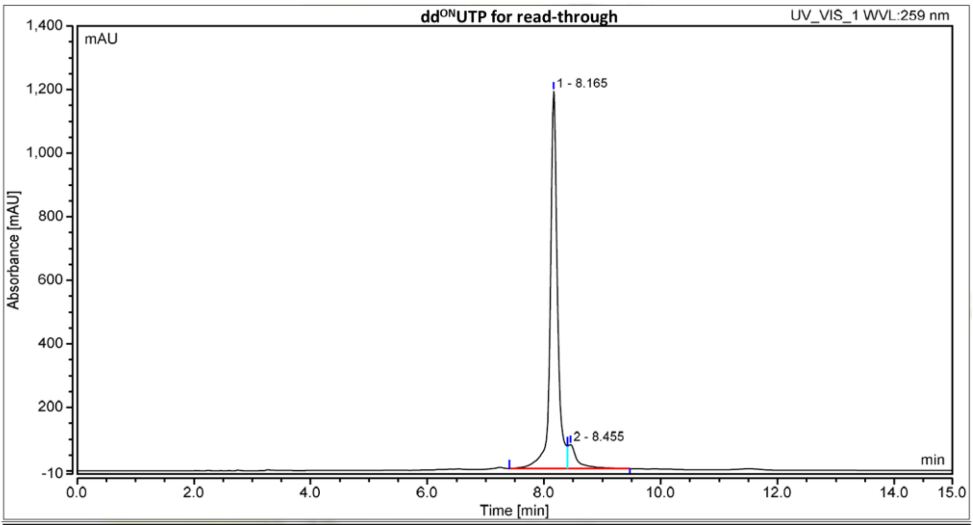

Integration Results

No.	Peak Name	Retention Time<br>min	Area<br>mAU*min	Height<br>mAU	Relative Area<br>%	Relative Height<br>%	Amount<br>n.a.
1		8.165	172.130	1187.467	92.06	94.00	n.a.
2		8.455	14.856	75.740	7.94	6.00	n.a.
**Total:**			**186.986**	**1263.207**	**100.00**	**100.00**	

Supplementary Figure S13. HPLC chromatogram of **dd^{ON}UTP** for read-through assay**.**

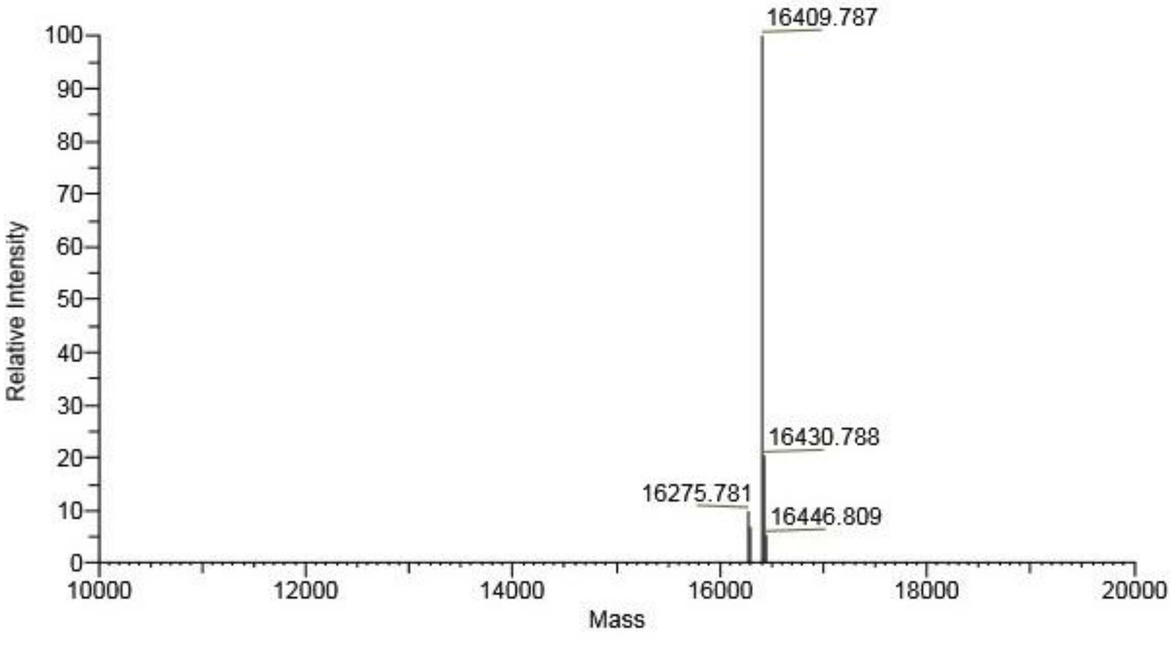

Supplementary Figure S14. Deconvoluted MS spectrum of **ON-dd^{ON}U** for read-through assay**.** HRMS (ESI$^-$): calculated monoisotopic mass for [M]: 16409.752; found: 16409.787.

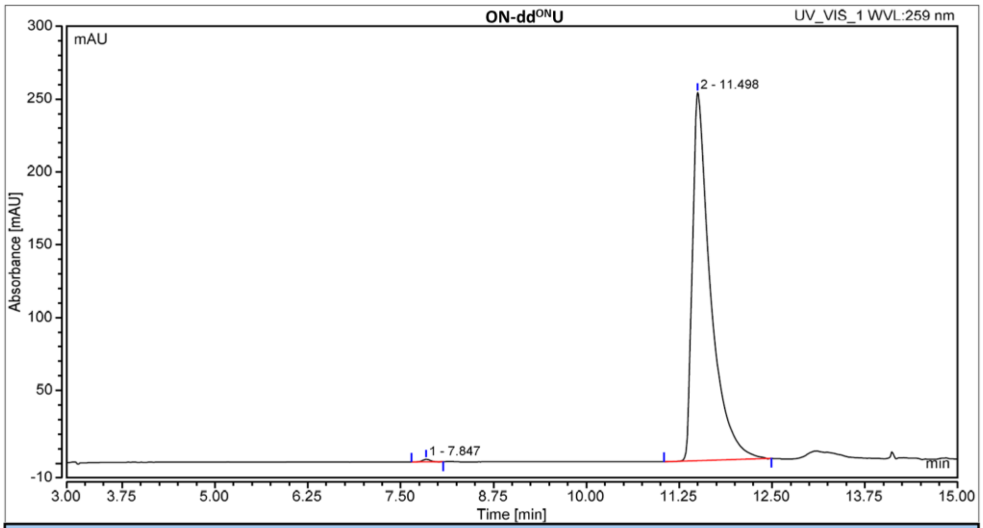

Integration Results

No.	Peak Name	Retention Time min	Area mAU*min	Height mAU	Relative Area %	Relative Height %	Amount n.a.
1		7.847	0.205	1.733	0.29	0.68	n.a.
2		11.498	71.273	252.826	99.71	99.32	n.a.
**Total:**			**71.478**	**254.559**	**100.00**	**100.00**	

Supplementary Figure S15. HPLC chromatogram of **ON-dd^{ON}U** for read-through assay**.**

Supplementary Note

1. NMR spectra

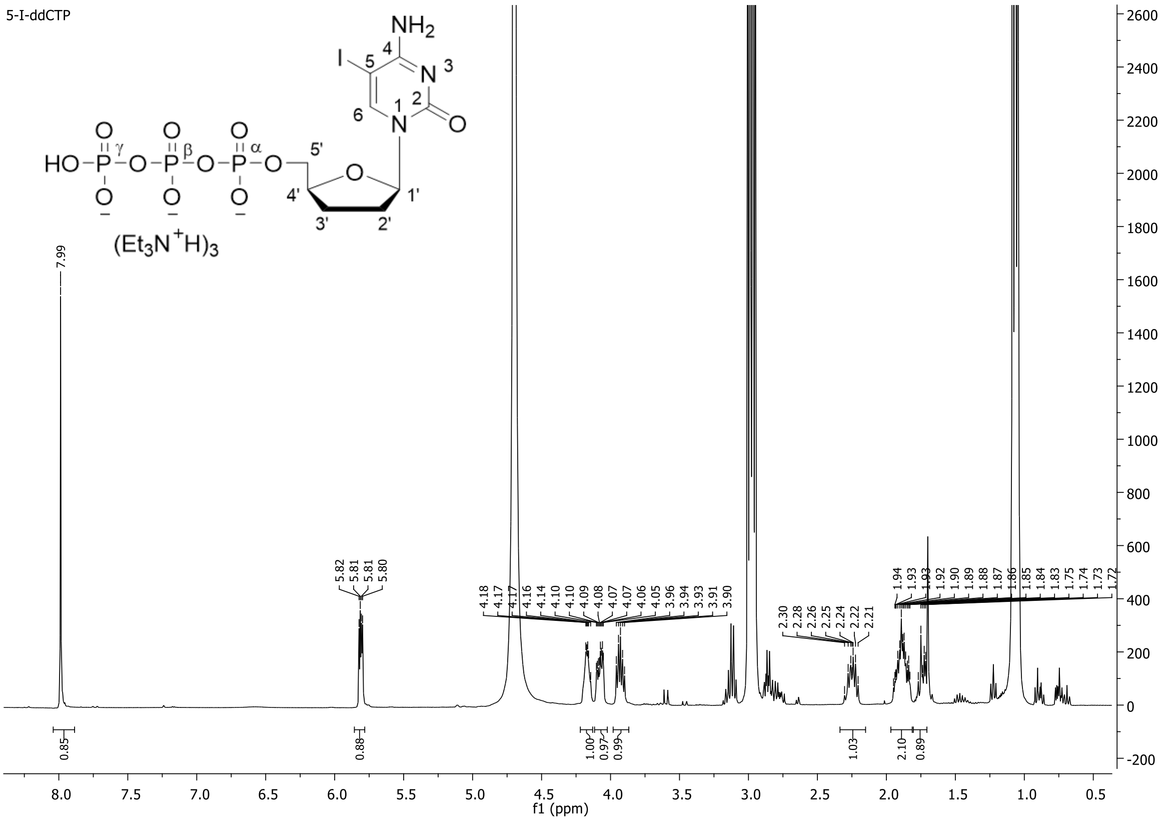

Supplementary Figure S16. ^{1}H NMR spectra of **dd^{I}CTP**

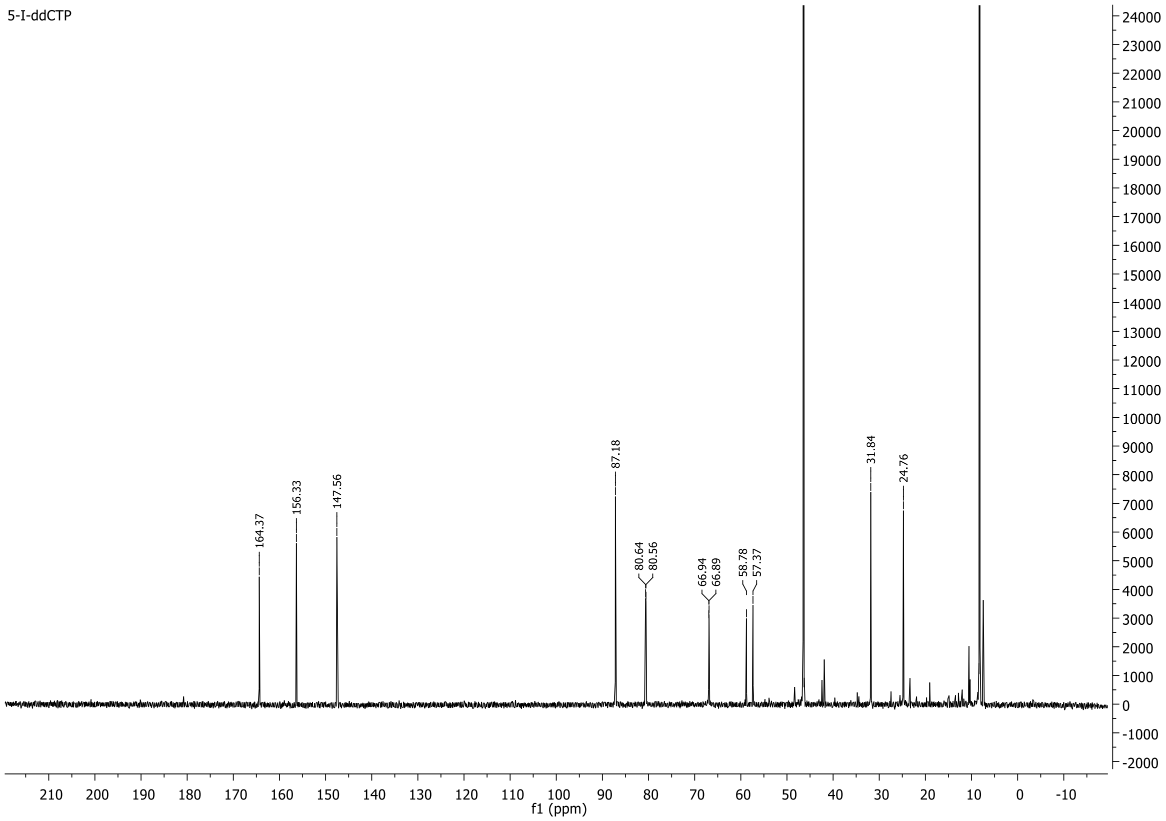

Supplementary Figure S17. ^{13}C NMR spectra of **dd^{I}CTP**

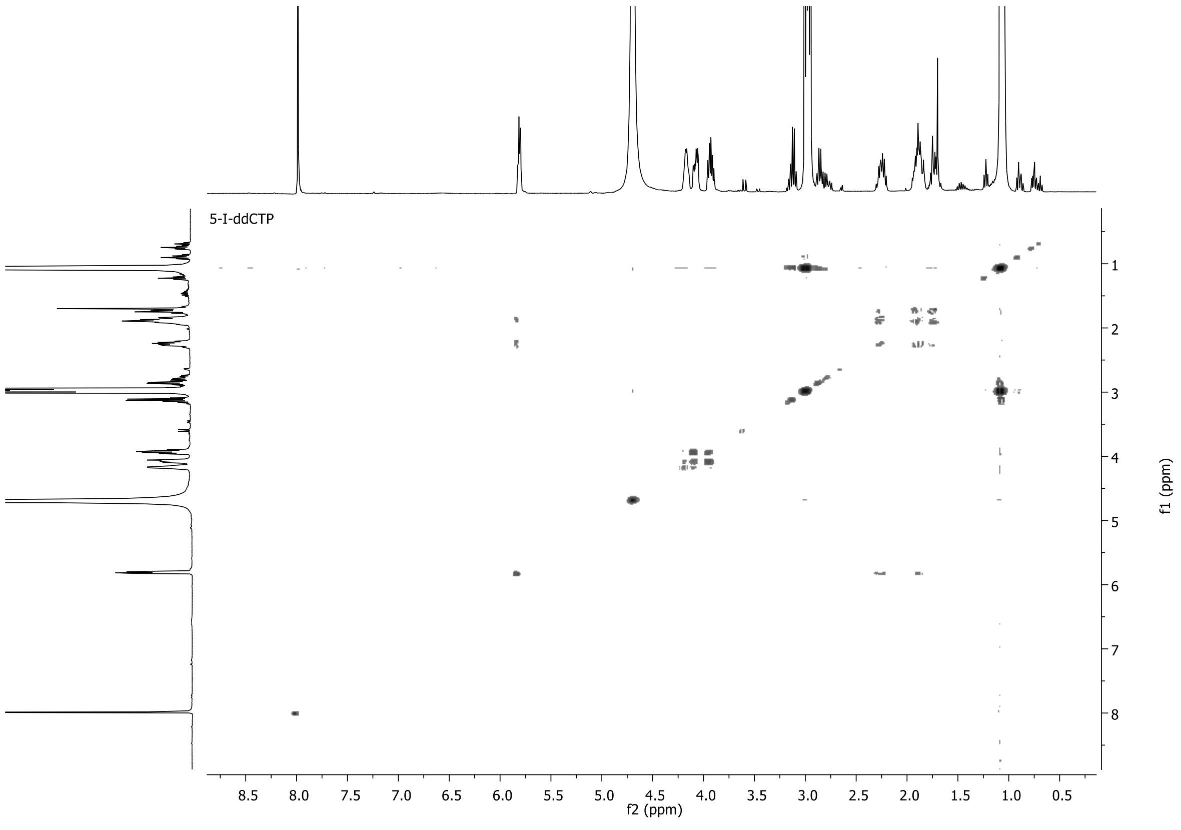

Supplementary Figure S18. H,H-COSY NMR spectra of **dd^{I}CTP**

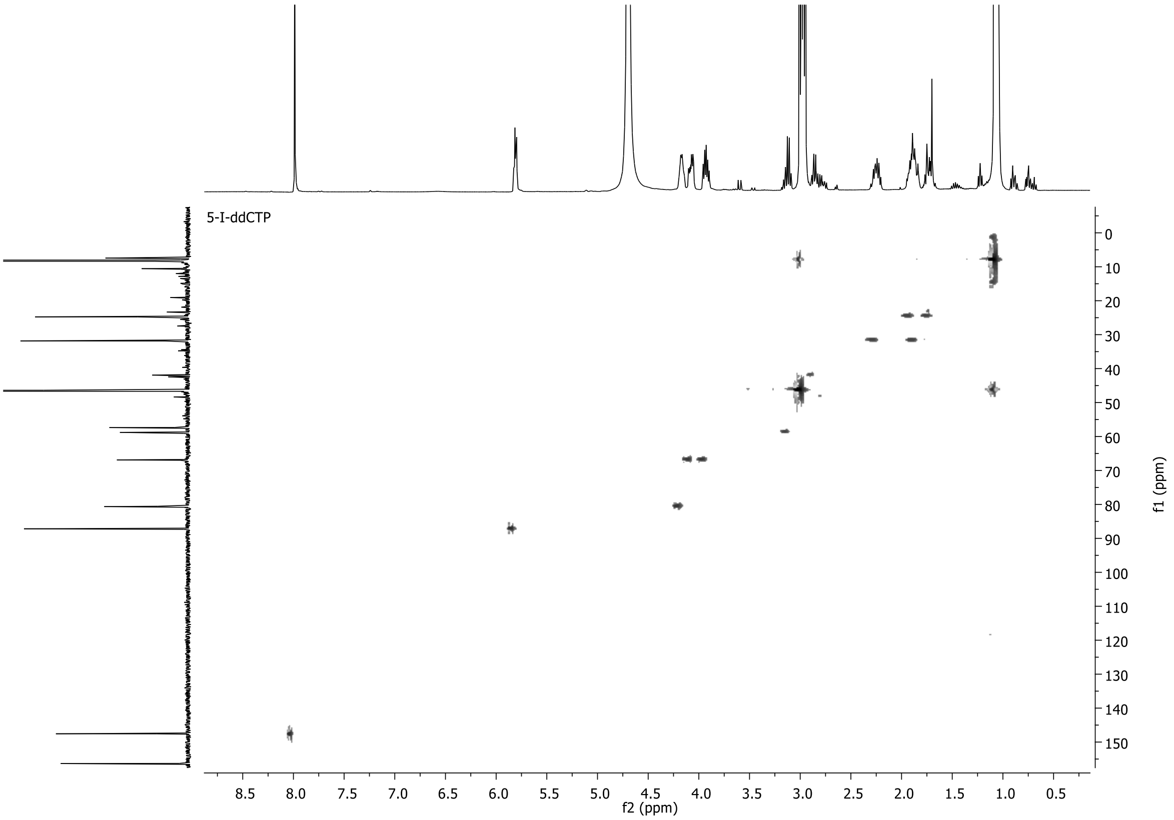

Supplementary Figure S19. H,C-HSQC NMR spectra of **dd^{I}CTP**

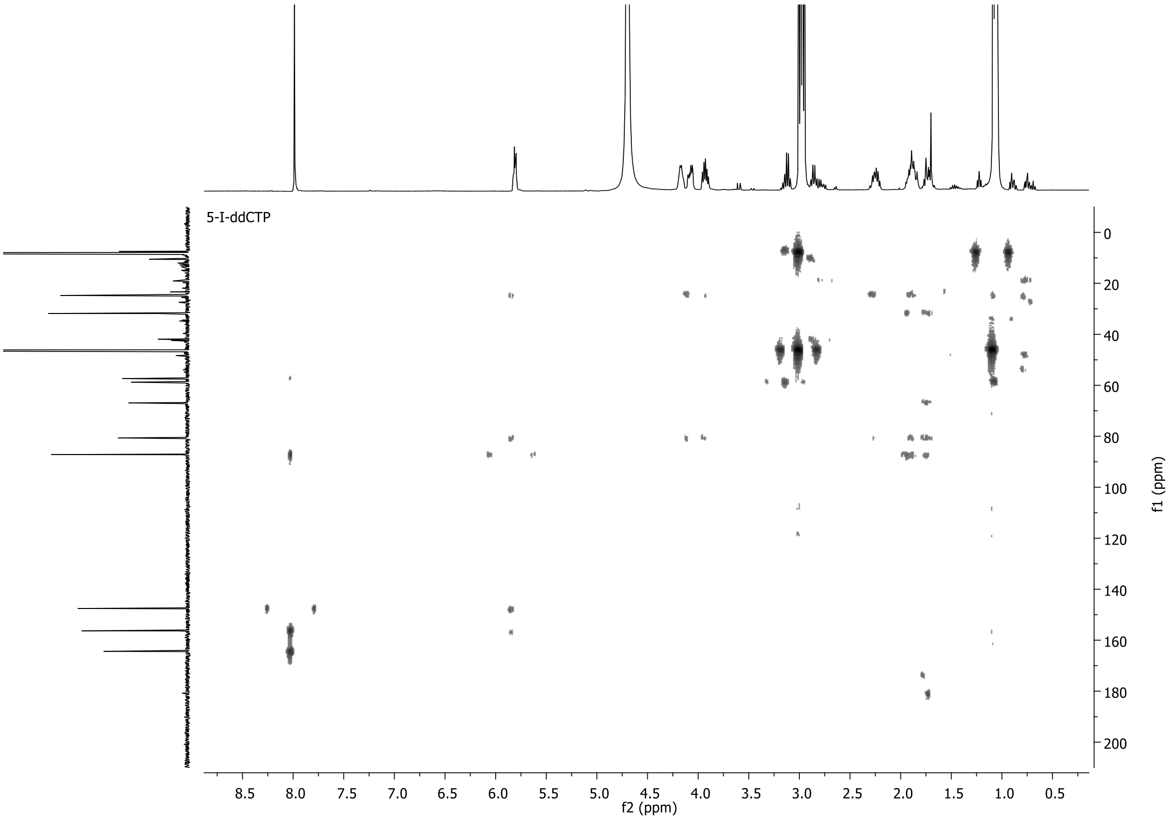

Supplementary Figure S20. H,C-HMBC NMR spectra of **dd^{I}CTP**

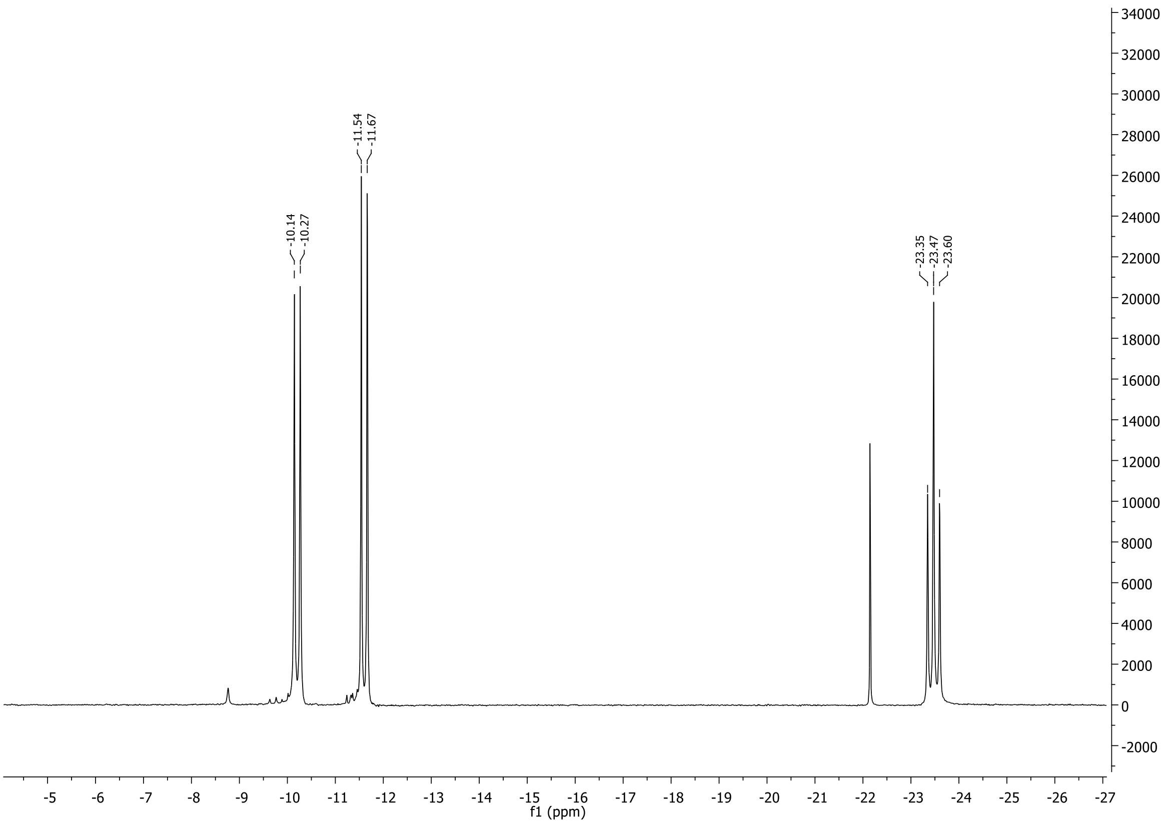

Supplementary Figure S21. ^{31}P NMR spectra of **dd^{I}CTP**

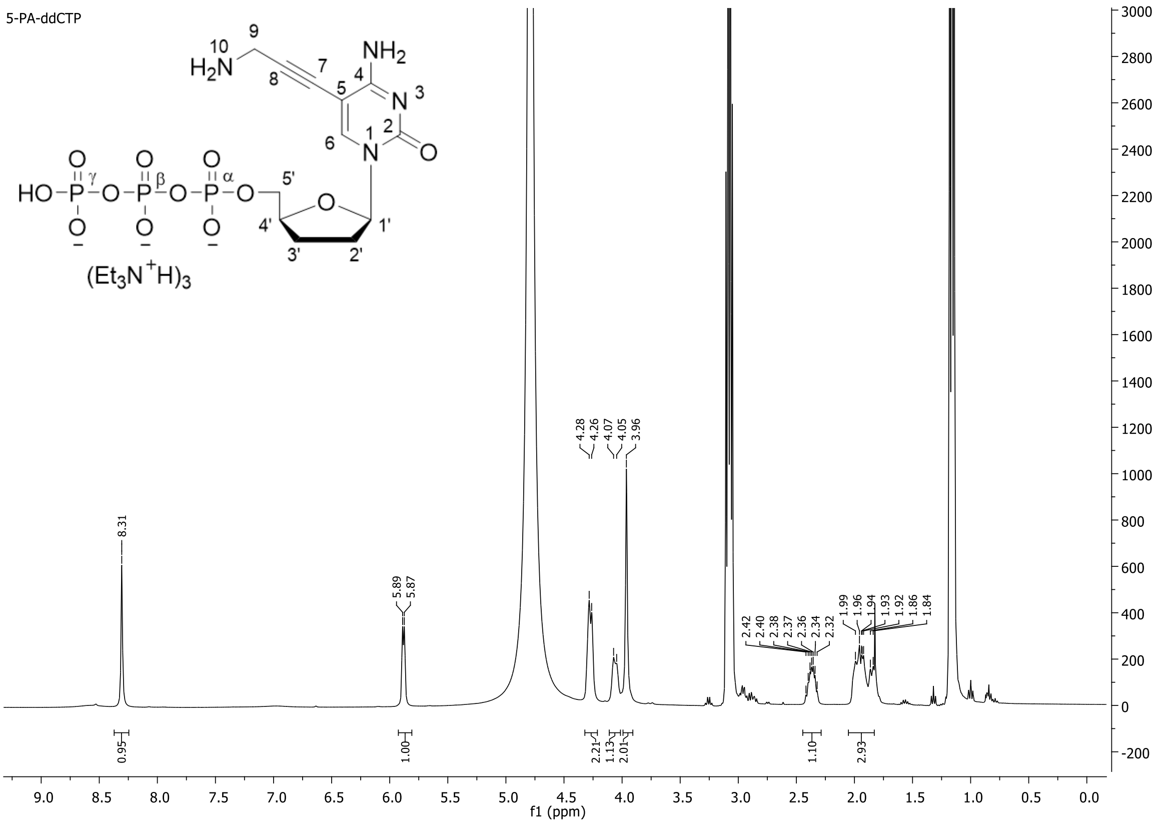

Supplementary Figure S22. ^{1}H NMR spectra of **dd^{PA}CTP**

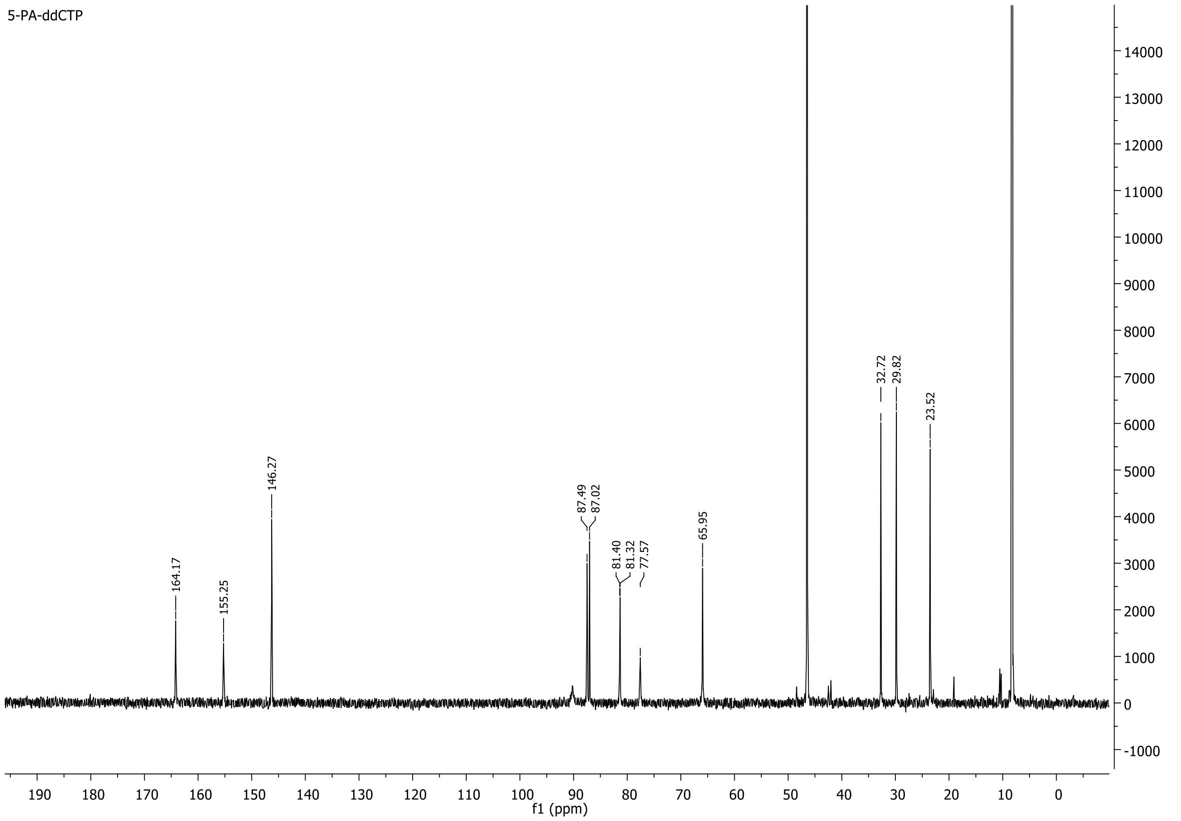

Supplementary Figure S23. ^{13}C NMR spectra of **dd^{PA}CTP**

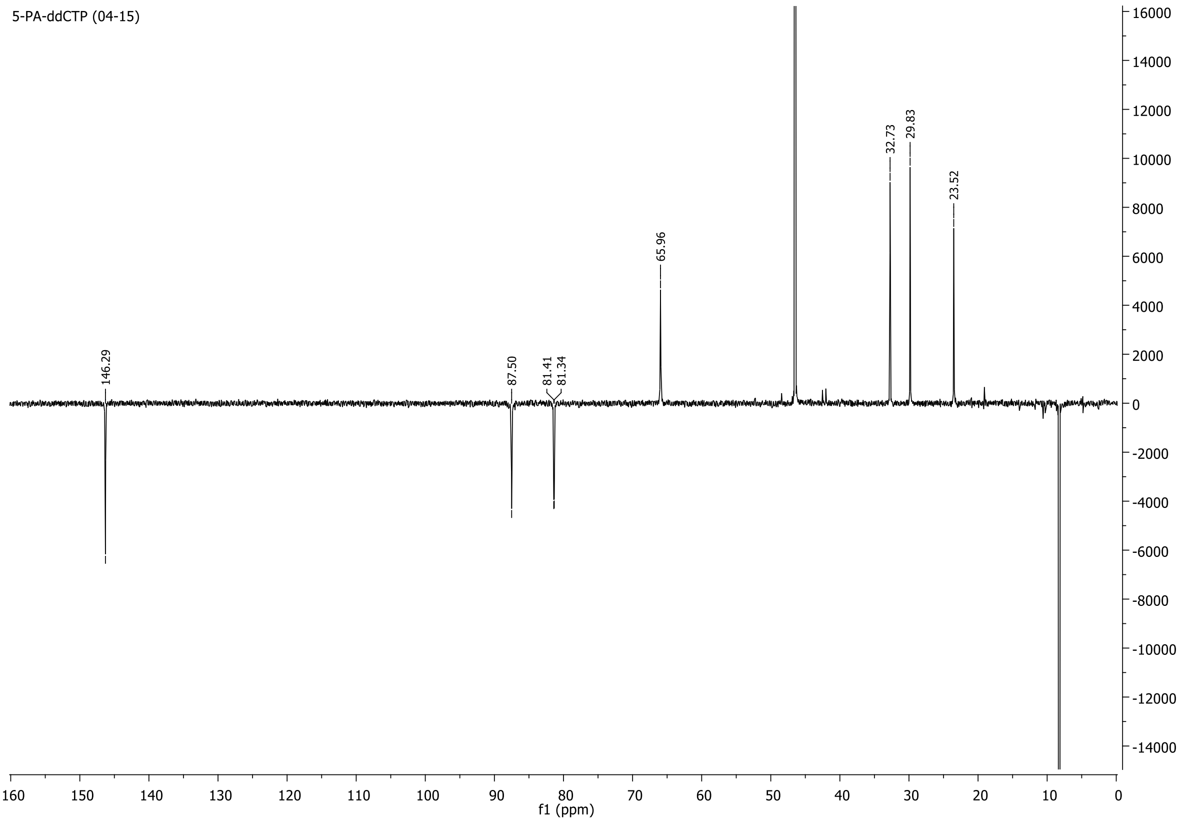

Supplementary Figure S24. ^{13}C Dept-135 NMR spectra of **dd^{PA}CTP**

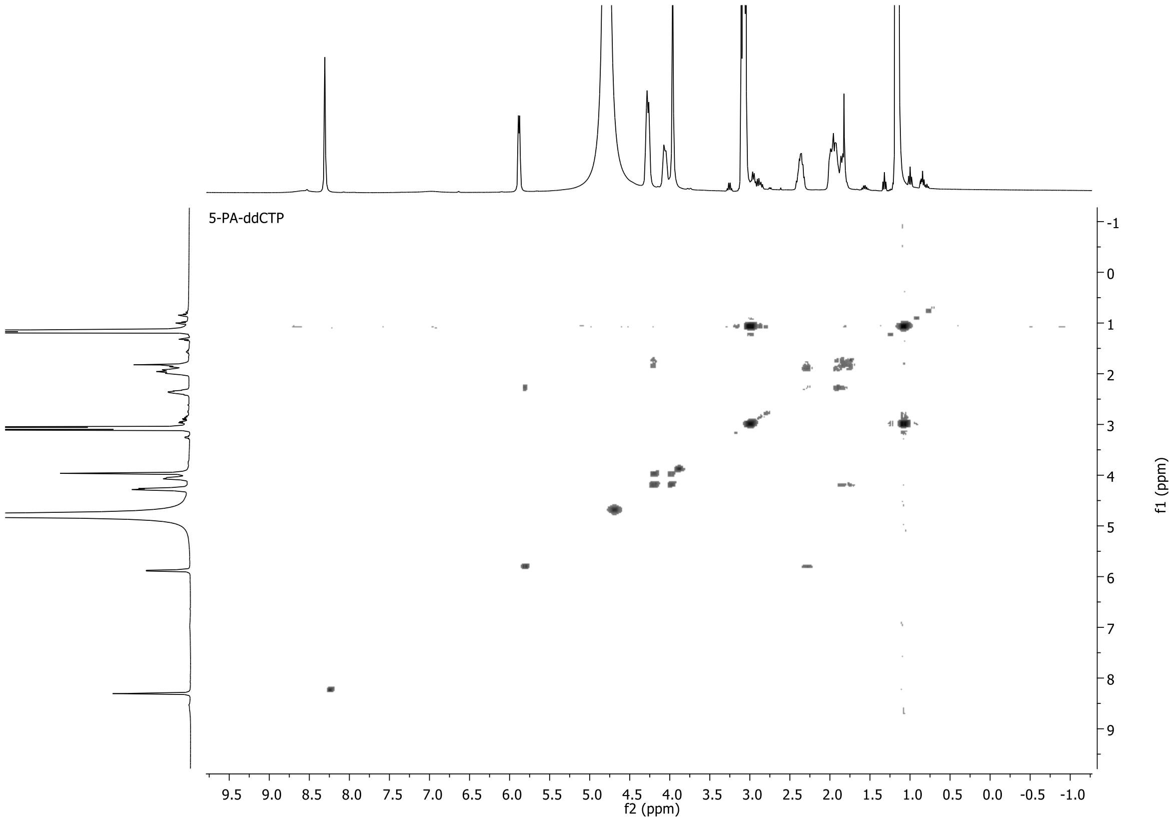

Supplementary Figure S25. H,H-COSY spectra of **dd^{PA}CTP**

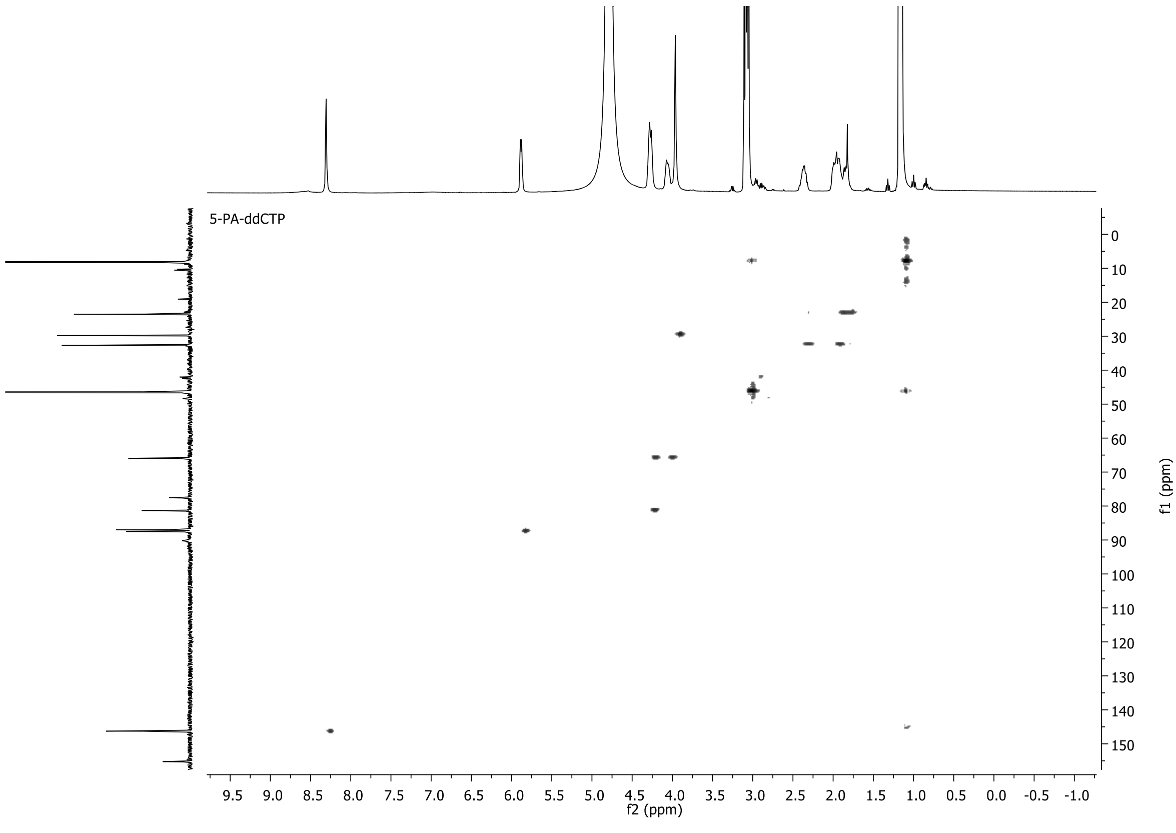

Supplementary Figure S26. H,C-HSQC spectra of **dd^{PA}CTP**

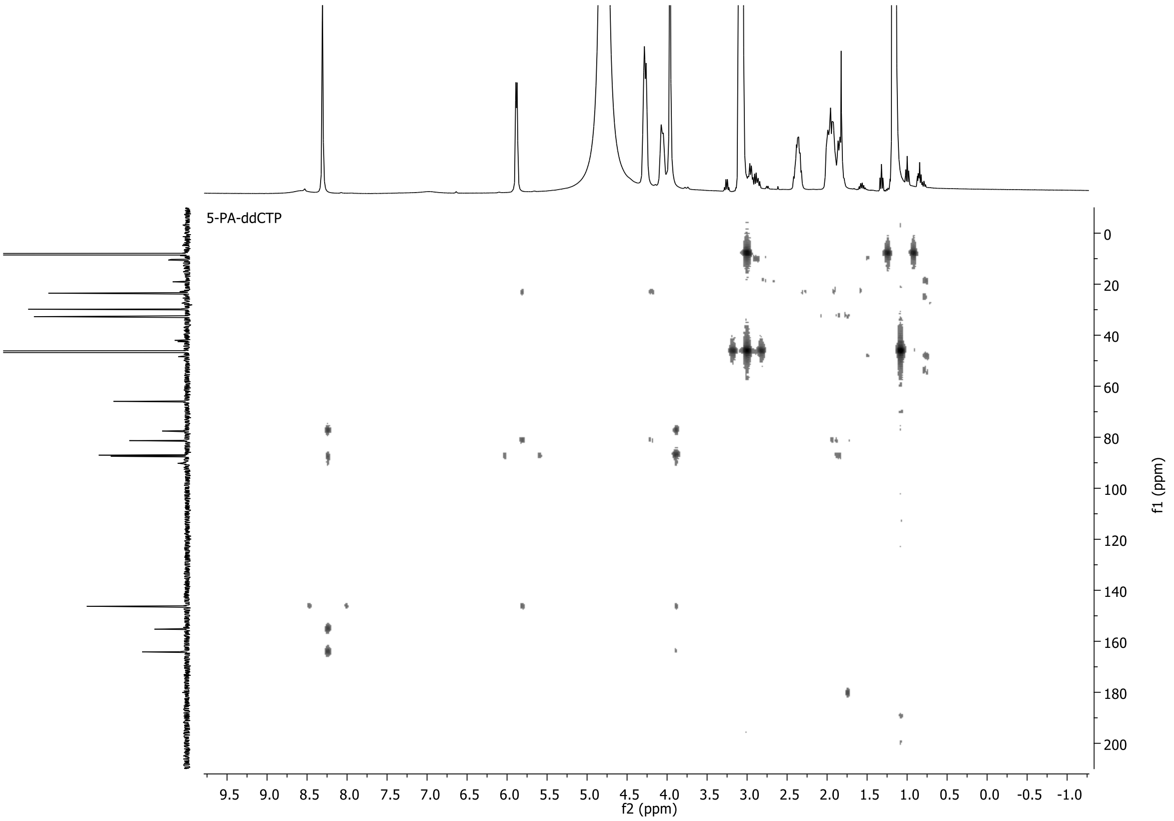

Supplementary Figure S27. H,C-HMBC spectra of **dd^{PA}CTP**

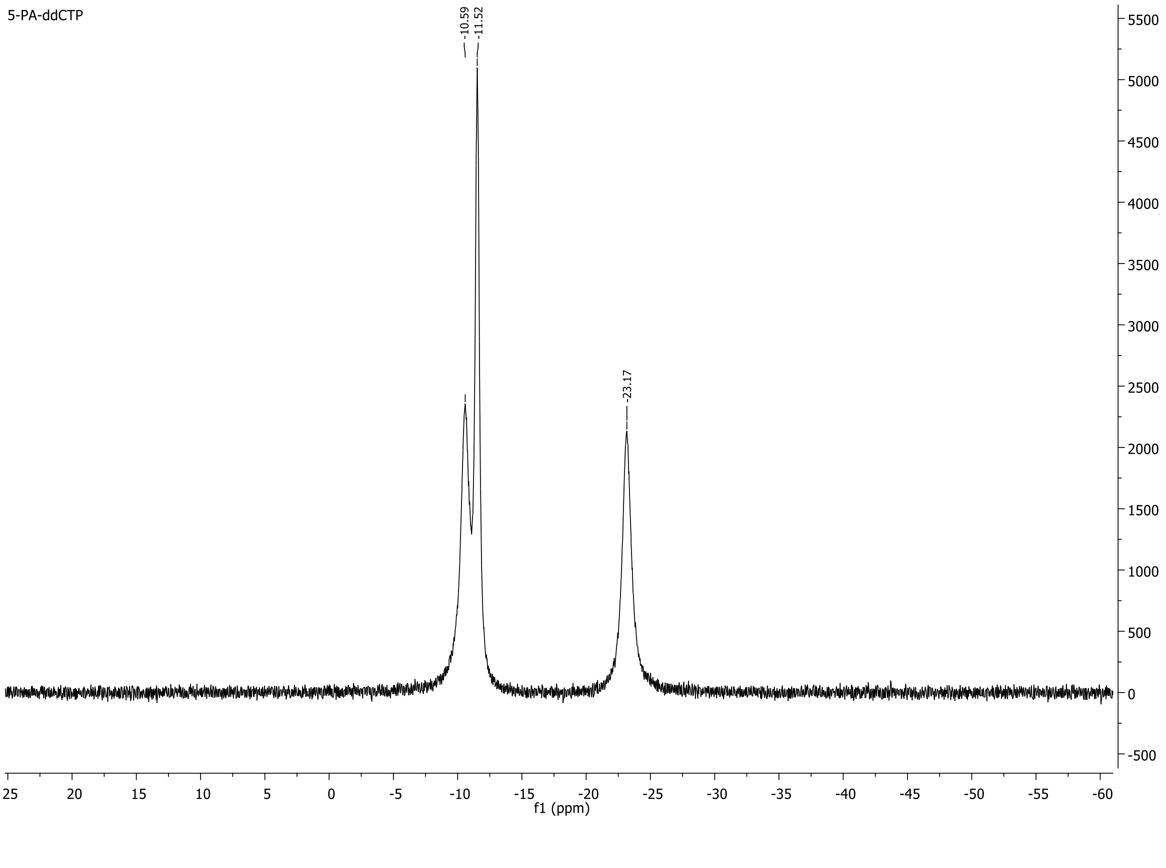

Supplementary Figure S28. ^{31}P spectra of **dd^{PA}CTP**

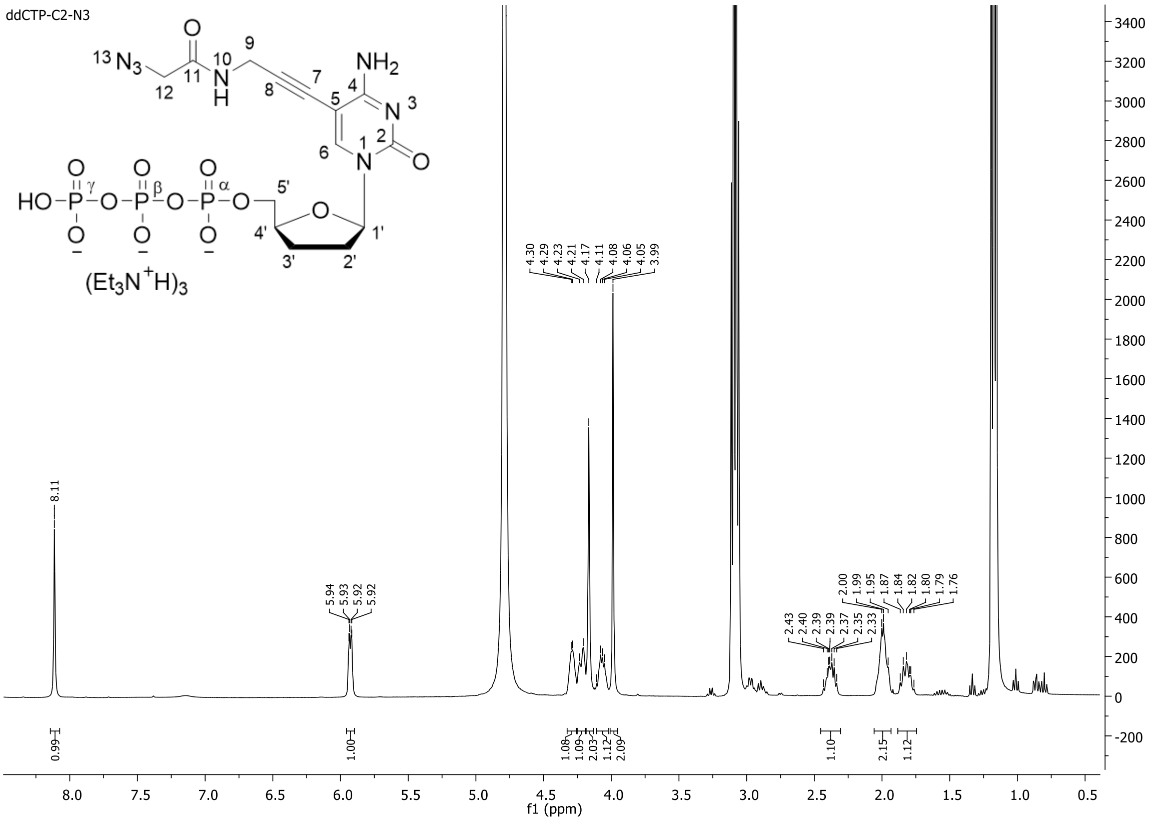

Supplementary Figure S29. ^{1}H spectra of **dd^{N3}CTP**

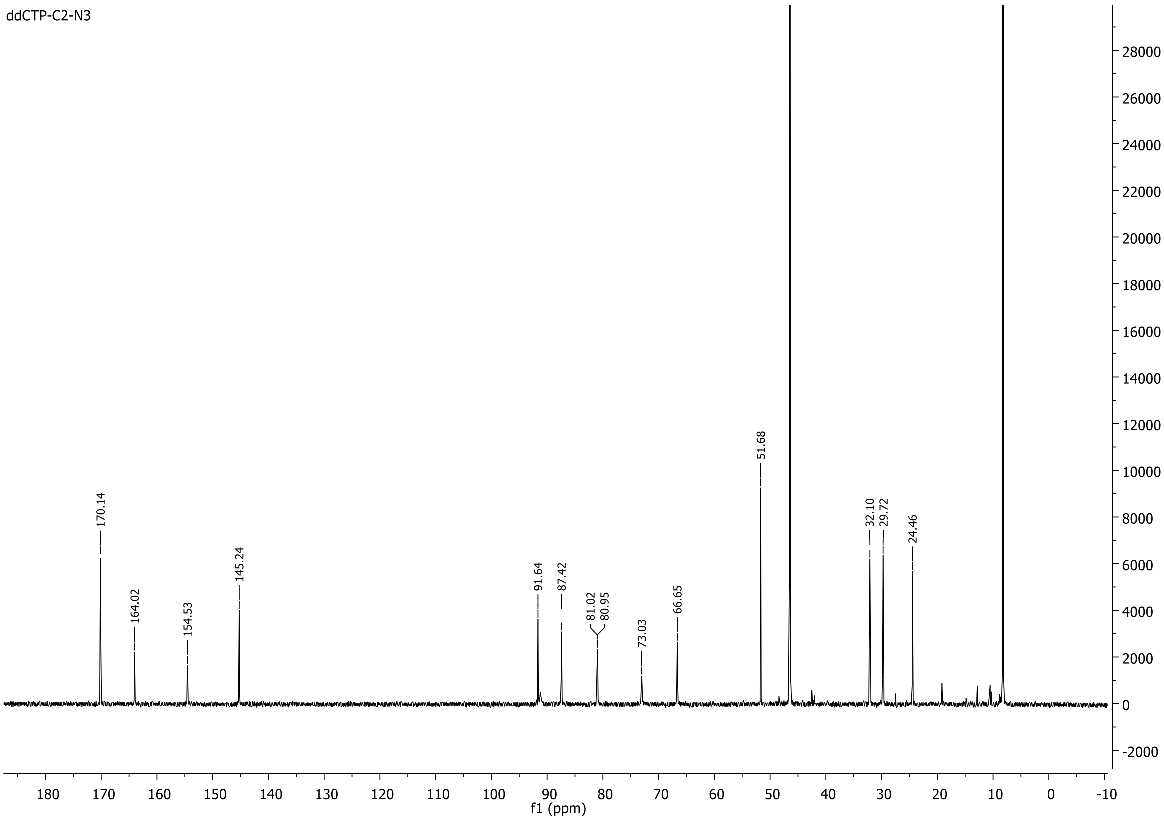

Supplementary Figure S30. ^{13}C spectra of **dd^{N3}CTP**

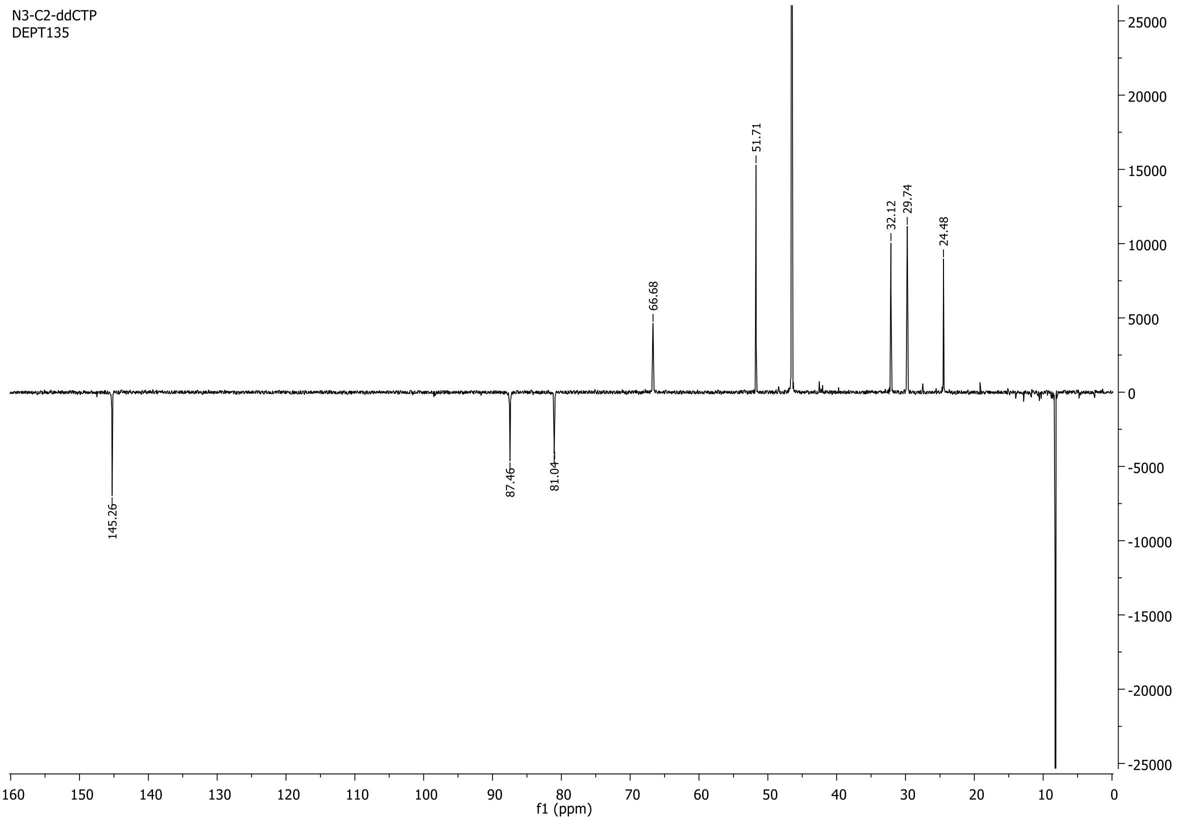

Supplementary Figure S31. ^{13}C Dept-135 of **dd^{N3}CTP**

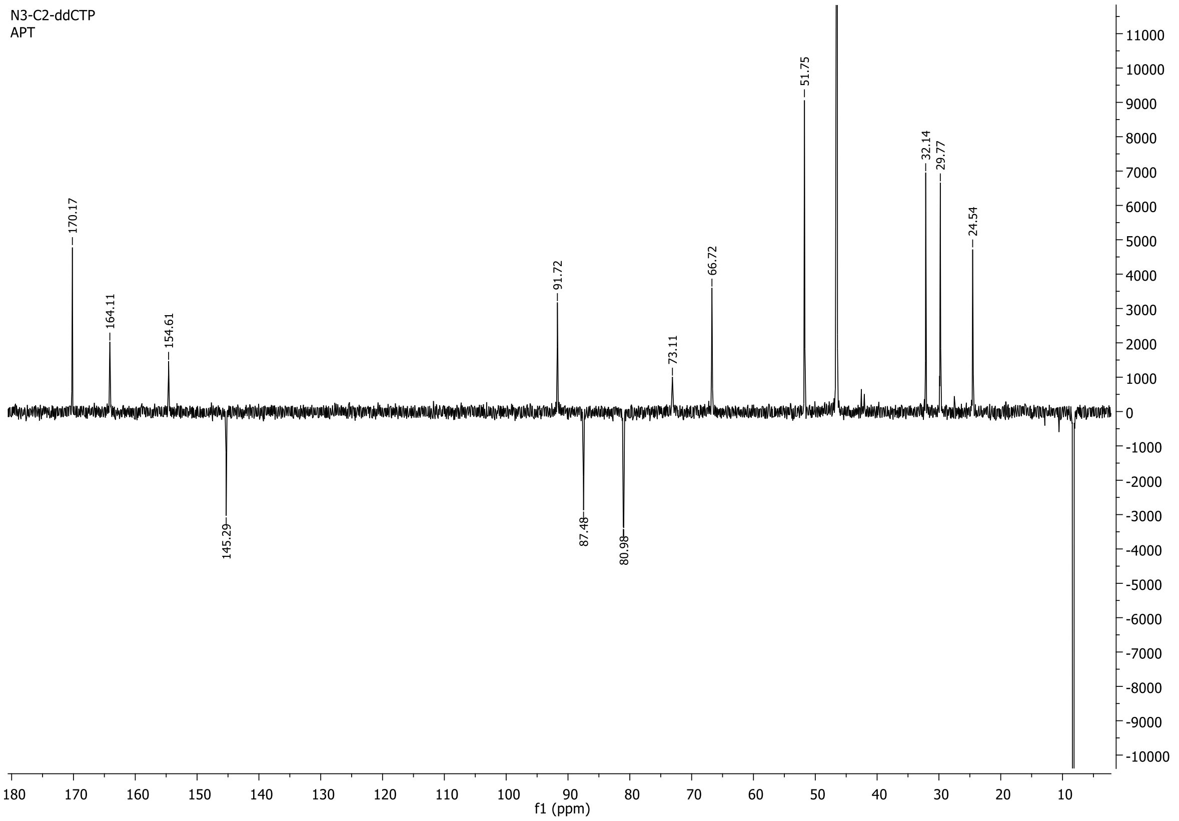

Supplementary Figure S32. ^{13}C APT of **dd^{N3}CTP**

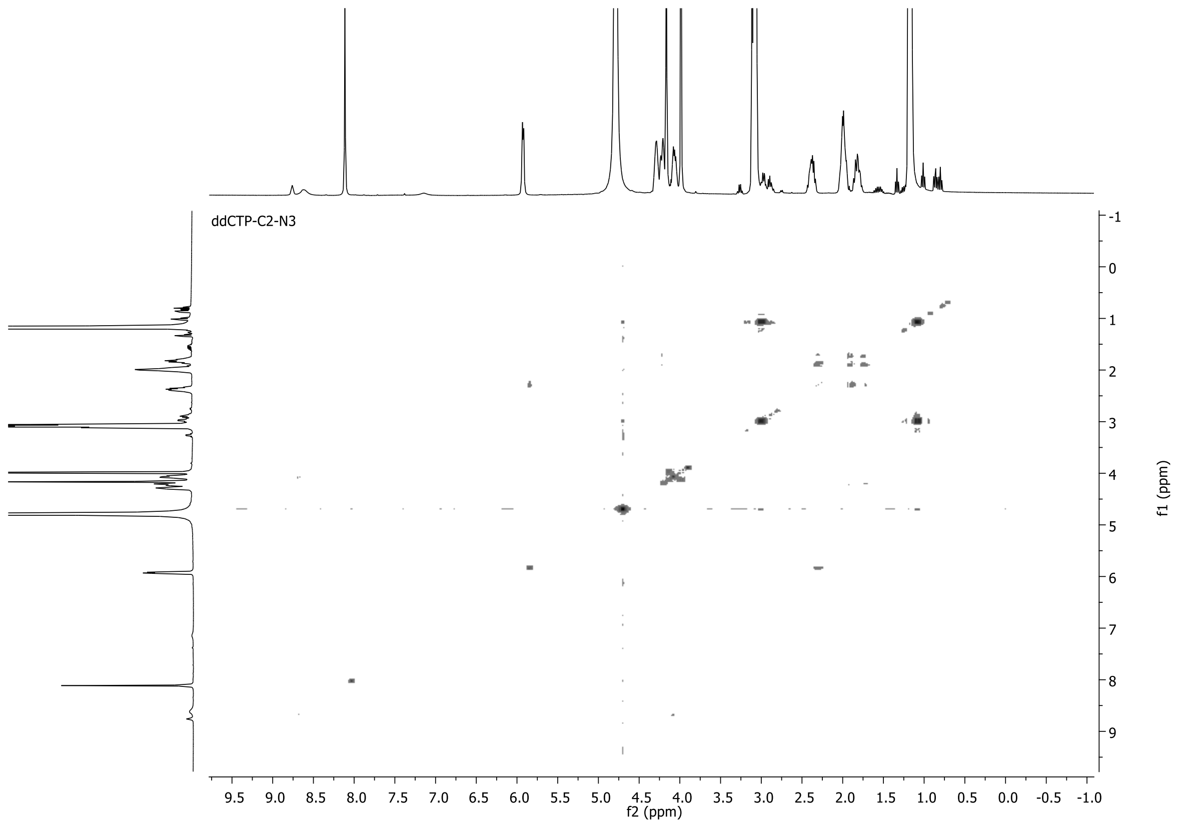

Supplementary Figure S33. H,H-COSY spectra of **dd^{N3}CTP**

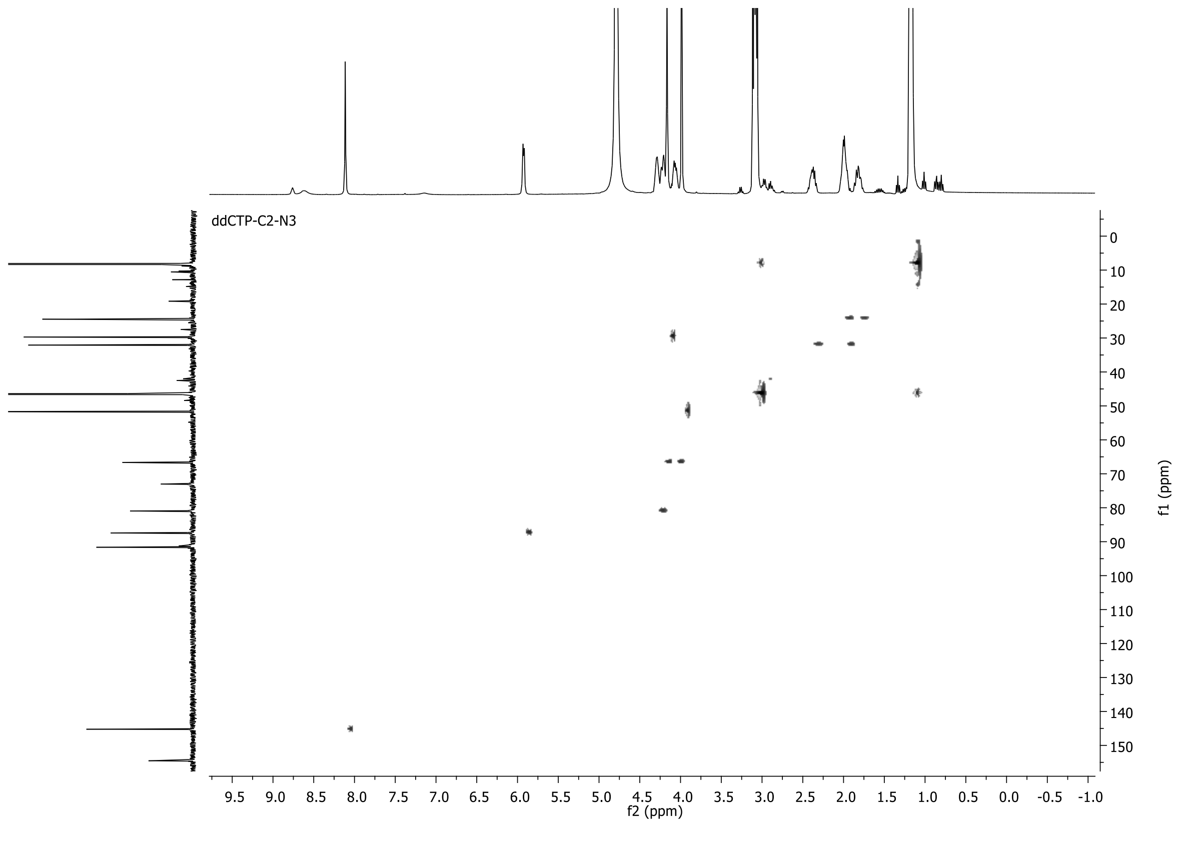

Supplementary Figure S34. H,C-HSQC spectra of **dd^{N3}CTP**

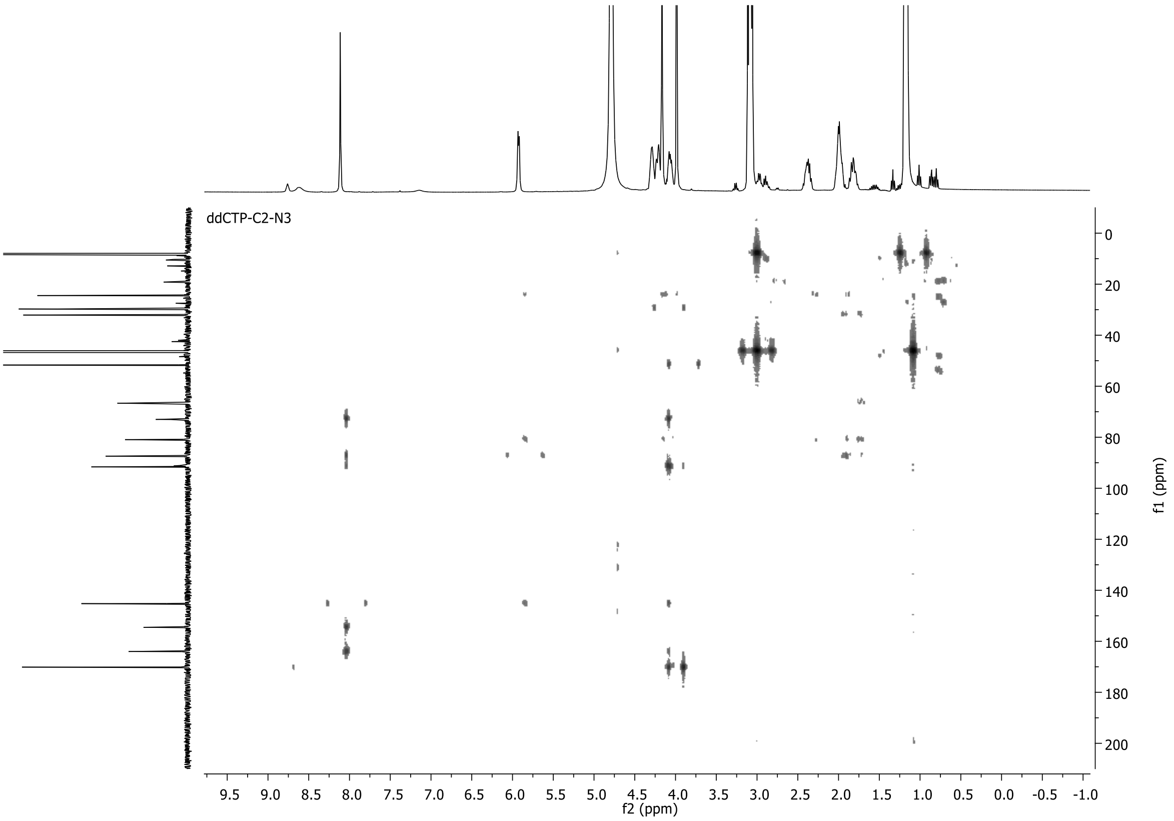

Supplementary Figure S35. H,C-HMBC spectra of **dd^{N3}CTP**

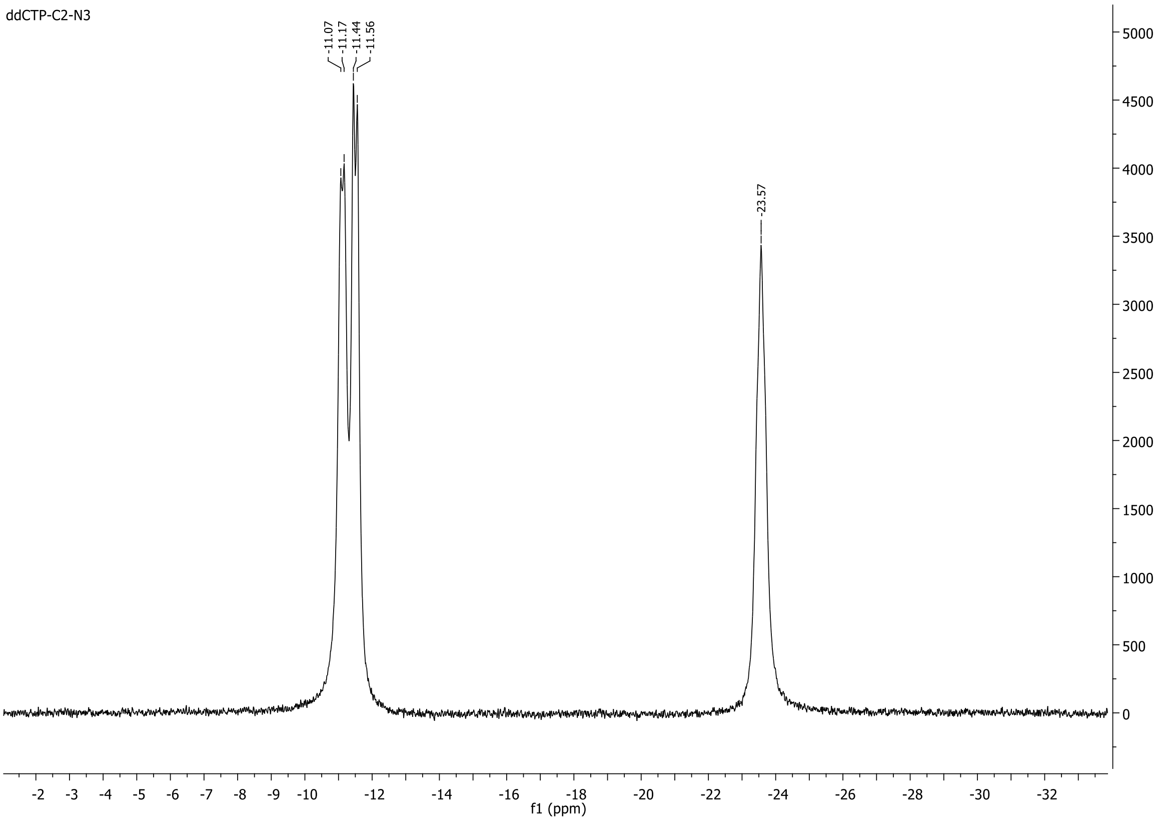

Supplementary Figure S36. ^{31}P spectra of **dd^{N3}CTP**

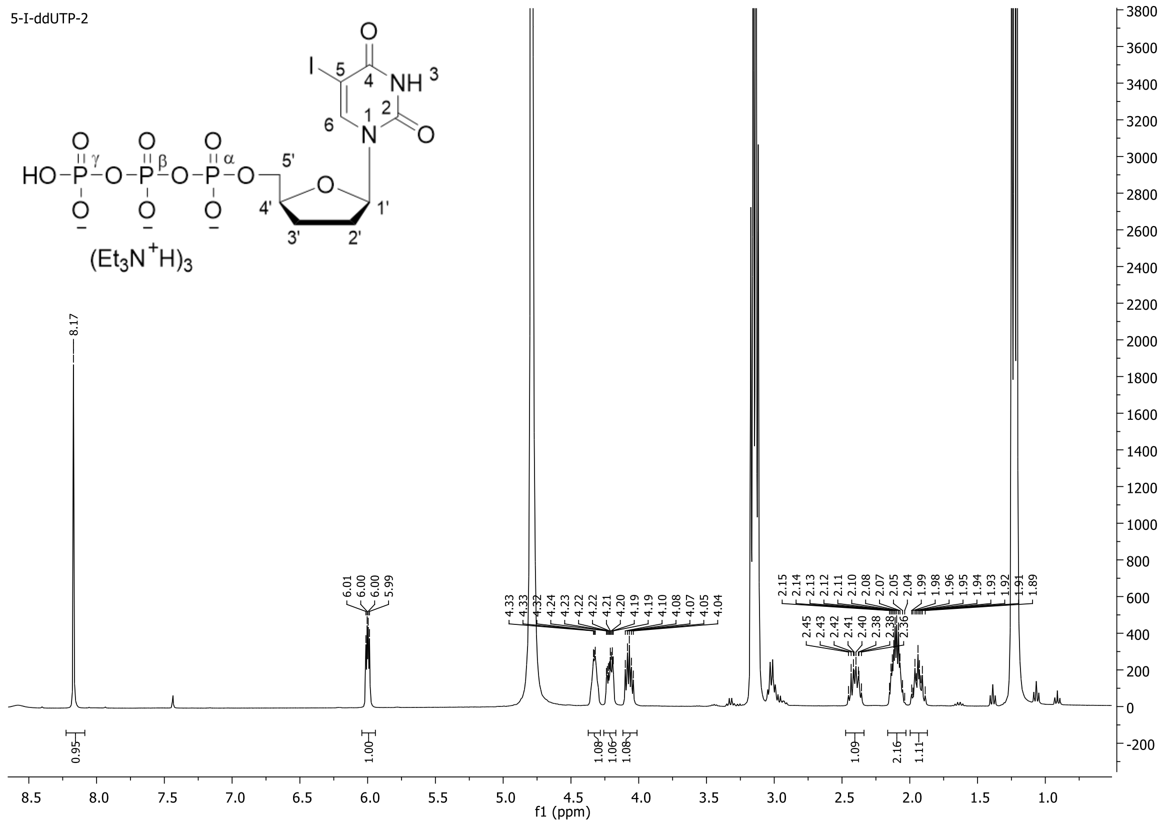

Supplementary Figure S37. ^{1}H spectra of **dd^{I}UTP**

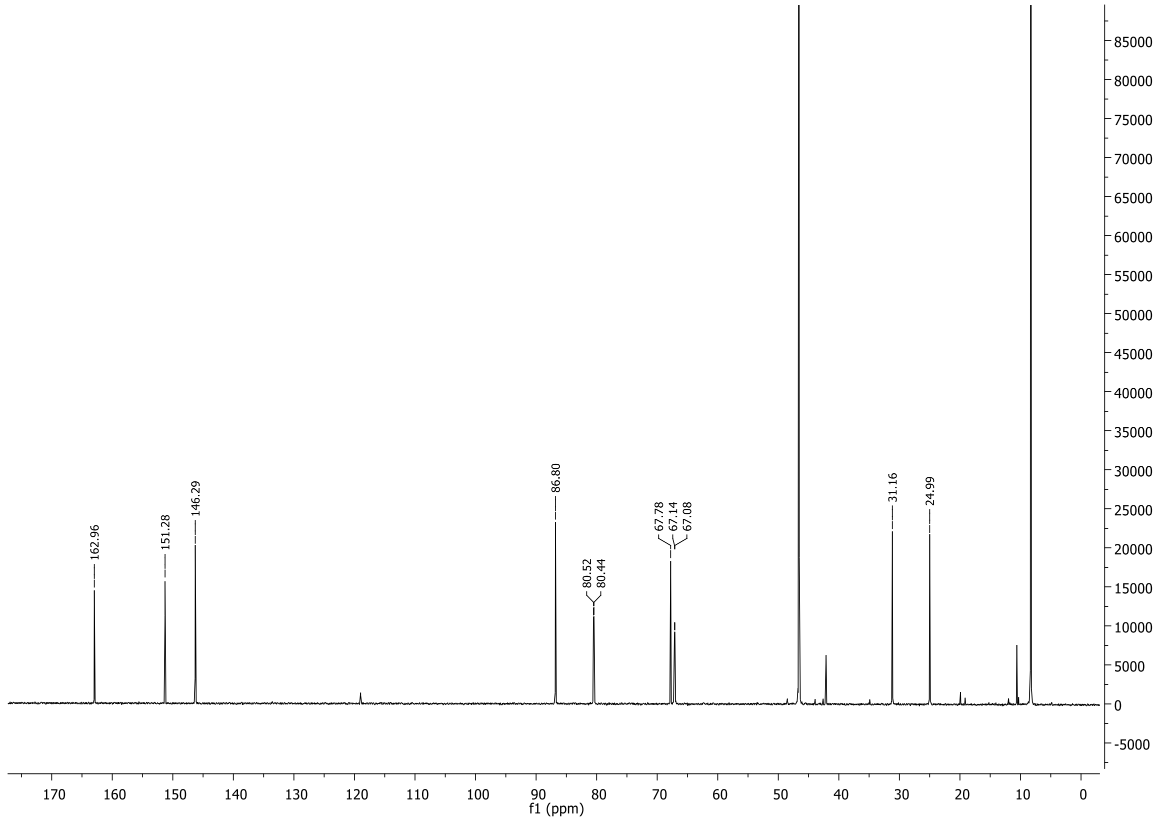

Supplementary Figure S38. ^{13}C spectra of **dd^{I}UTP**

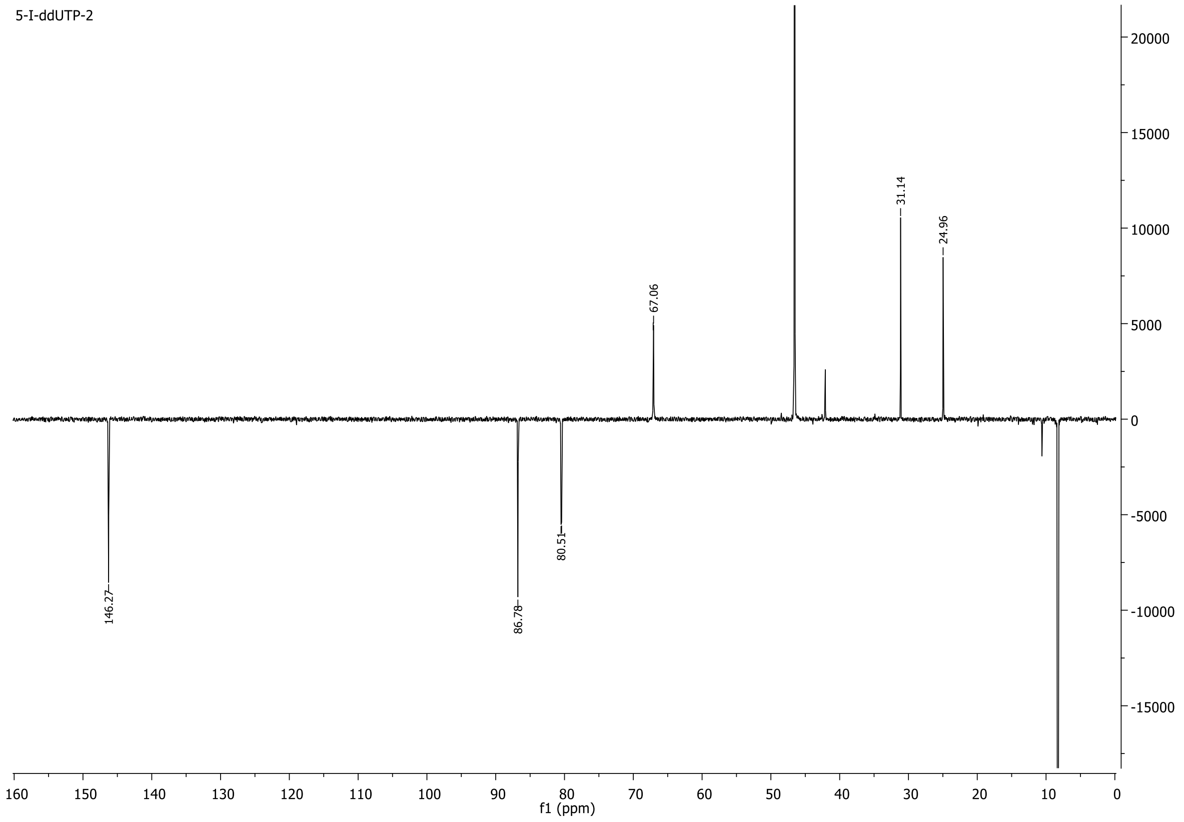

Supplementary Figure S39. ^{13}C Dept-135 spectra of **dd^{I}UTP**

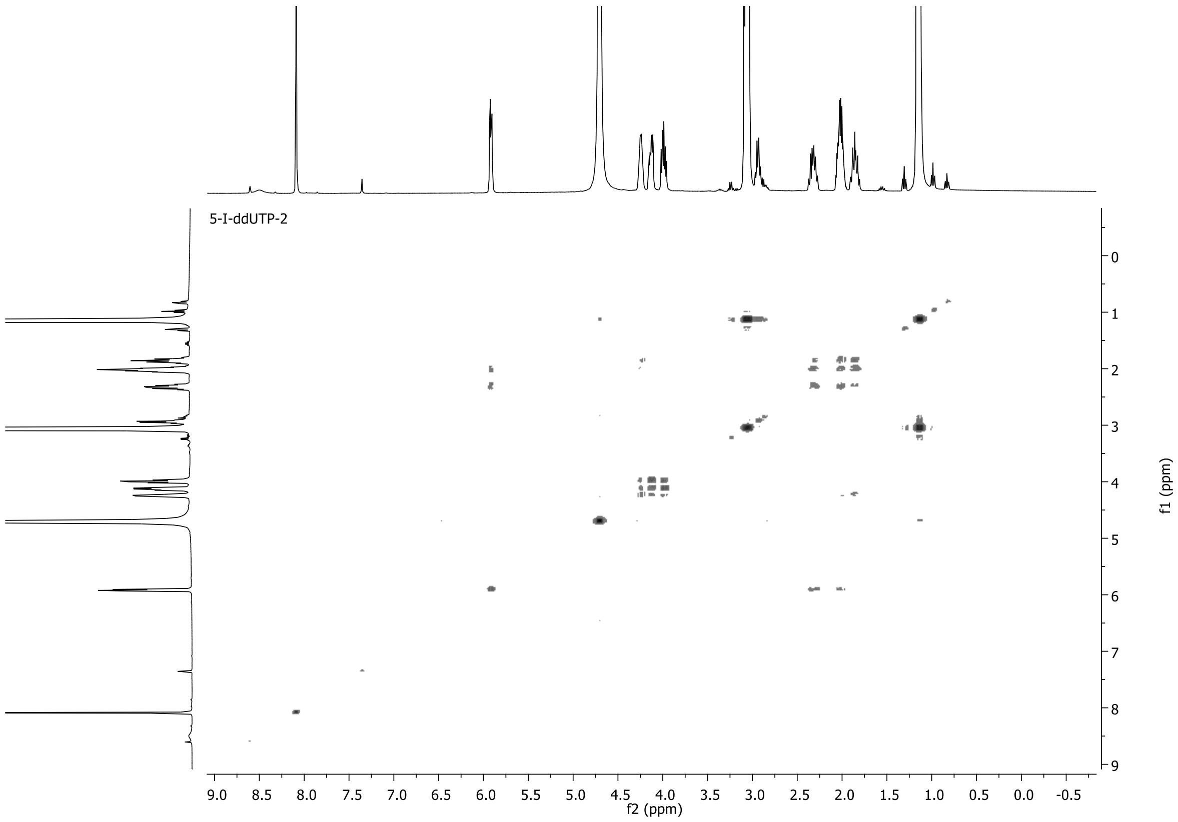

Supplementary Figure S40. H,C-COSY spectra of **dd^{I}UTP**

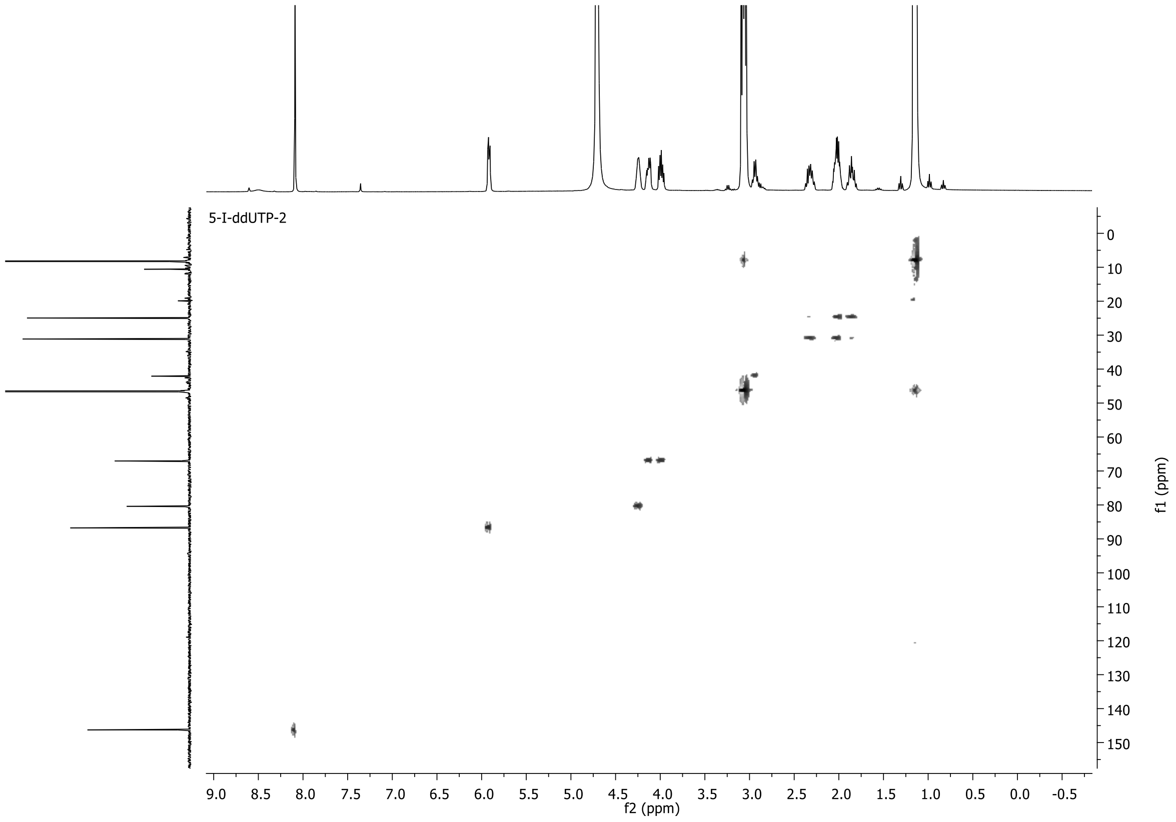

Supplementary Figure S41. H,C-HSQC spectra of **dd^{I}UTP**

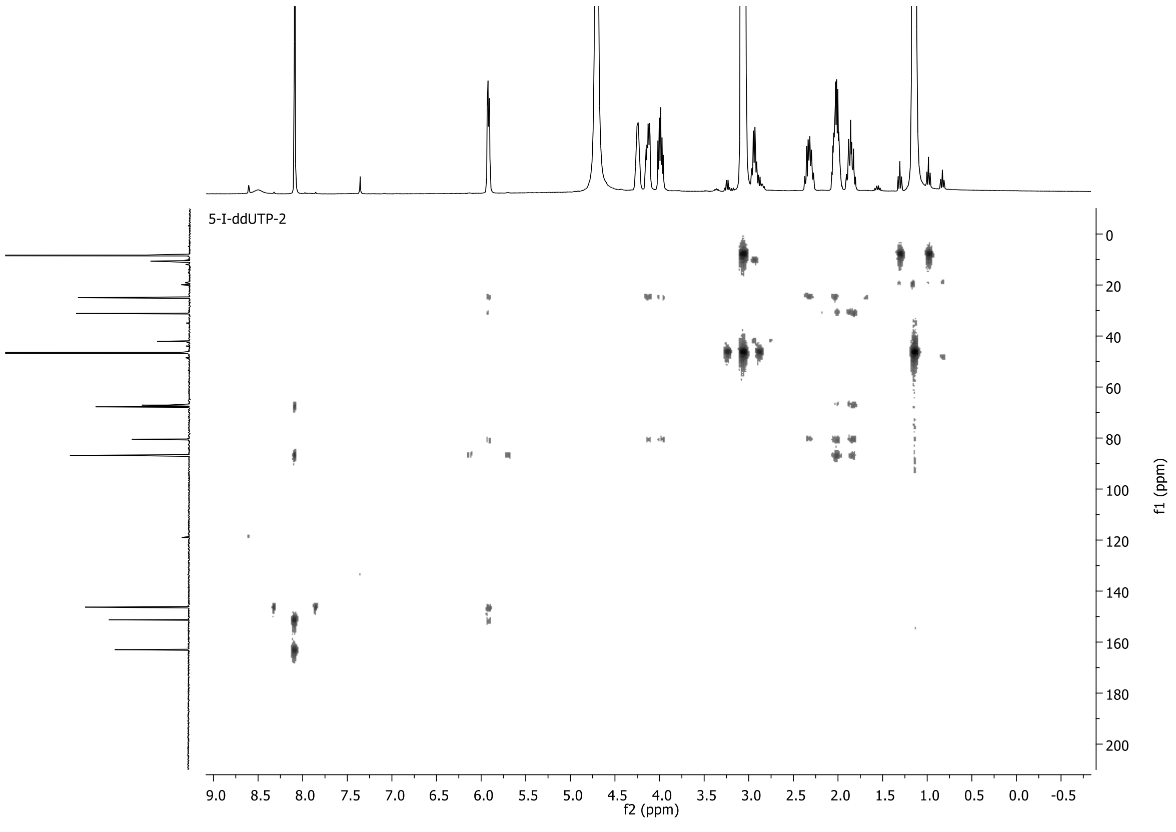

Supplementary Figure S42. H,C-HMBC spectra of **dd^{I}UTP**

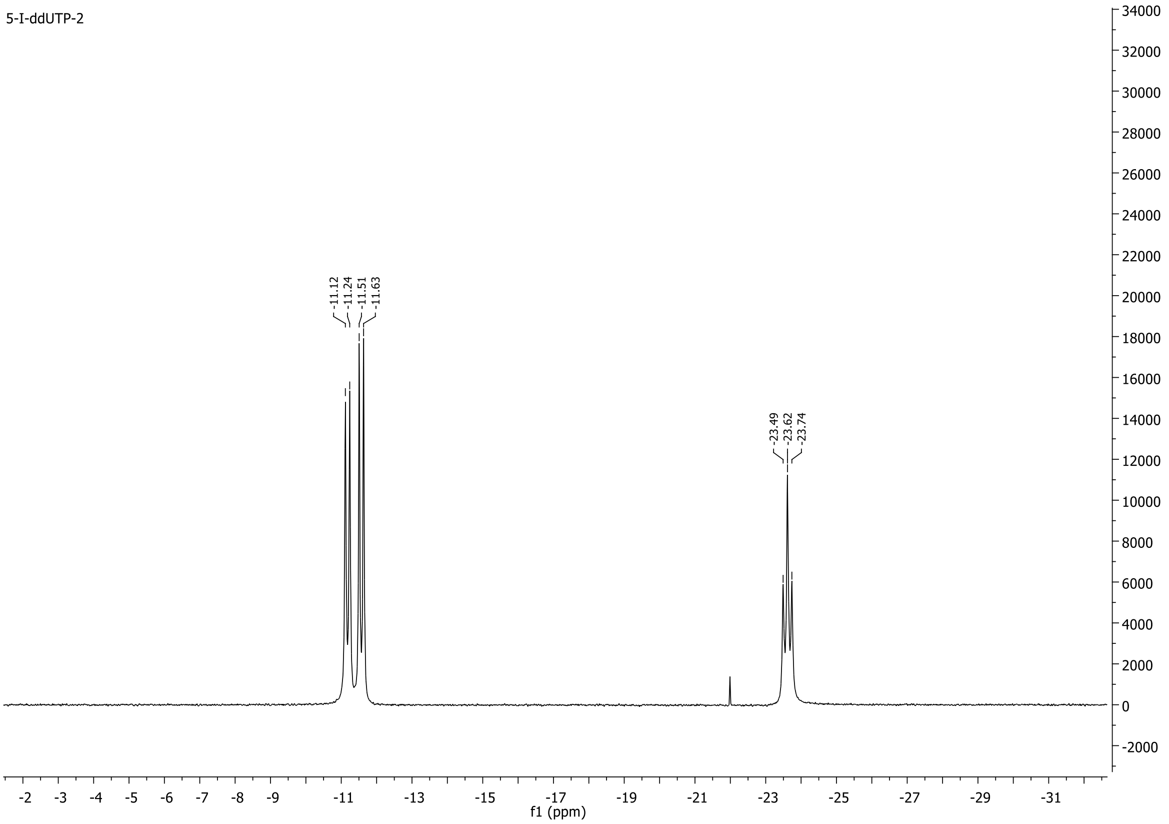

Supplementary Figure S43. ^{31}P spectra of **dd^{I}UTP**

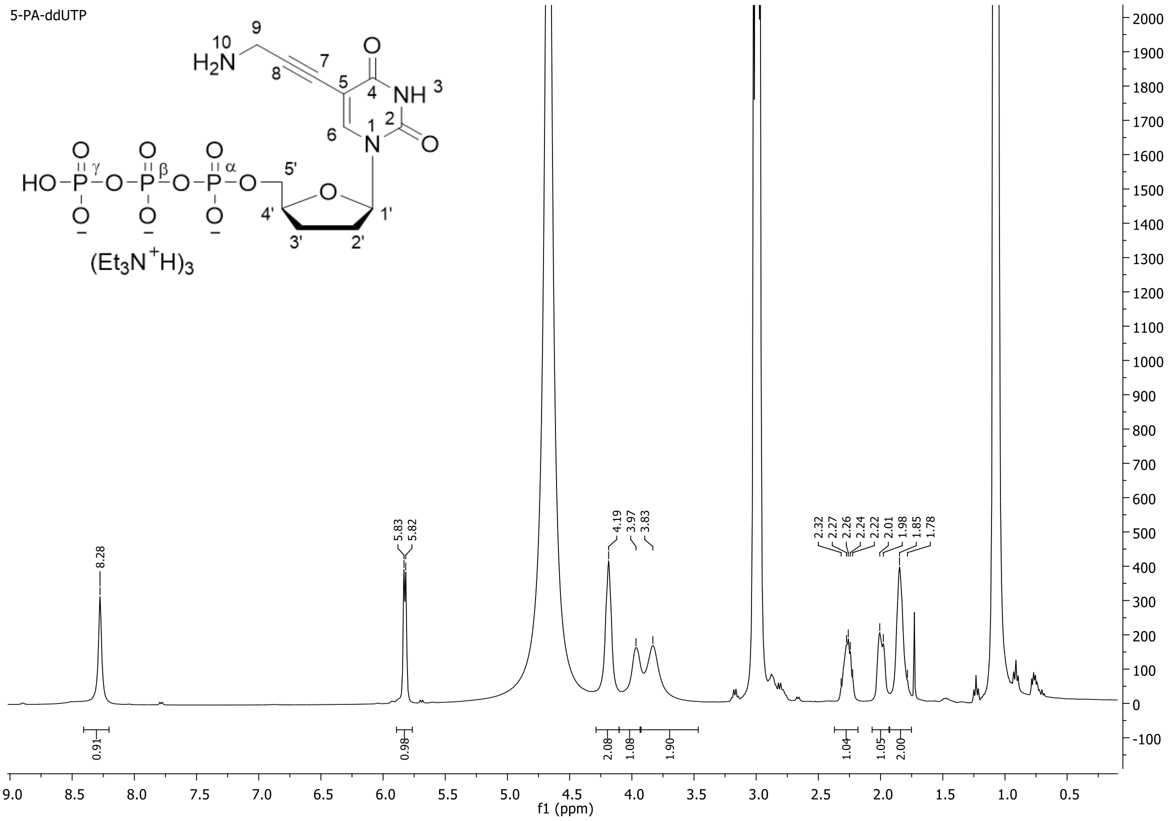

Supplementary Figure S44. ^{1}H spectra of **dd^{PA}UTP**

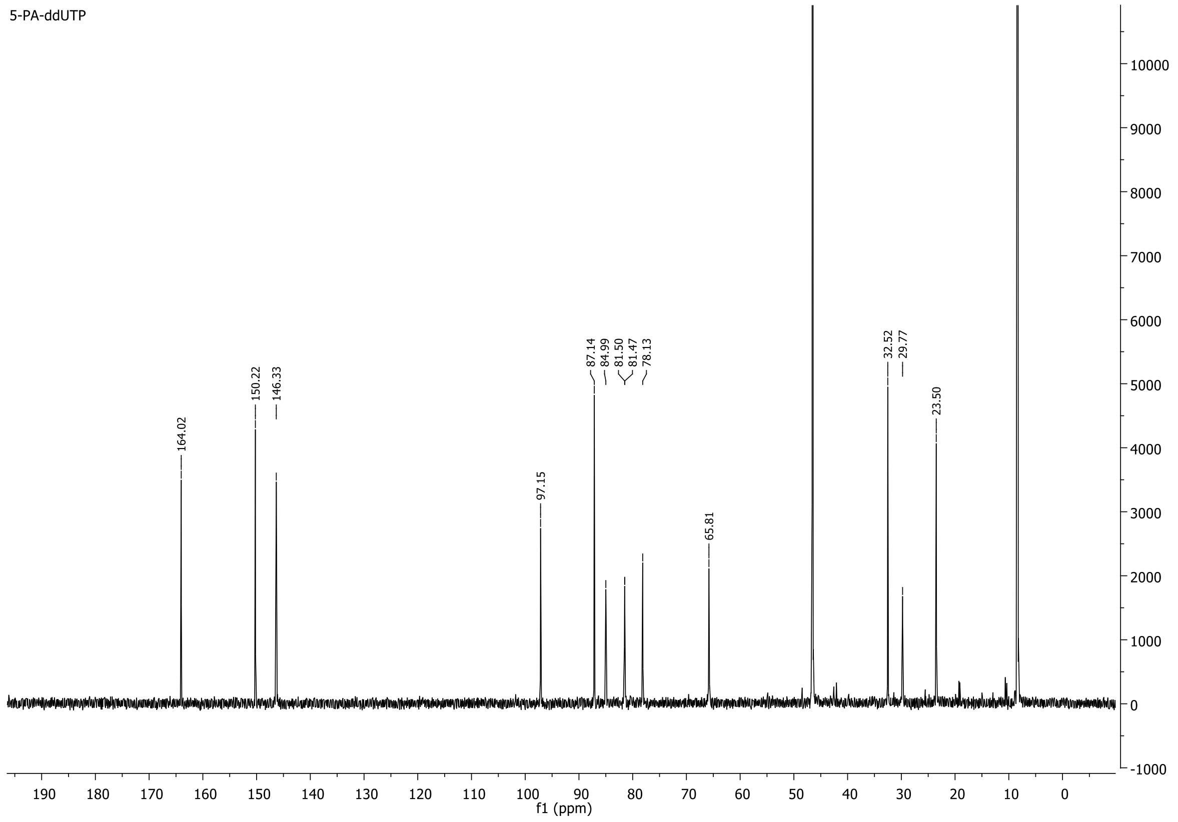

Supplementary Figure S45. ^{13}C spectra of **dd^{PA}UTP**

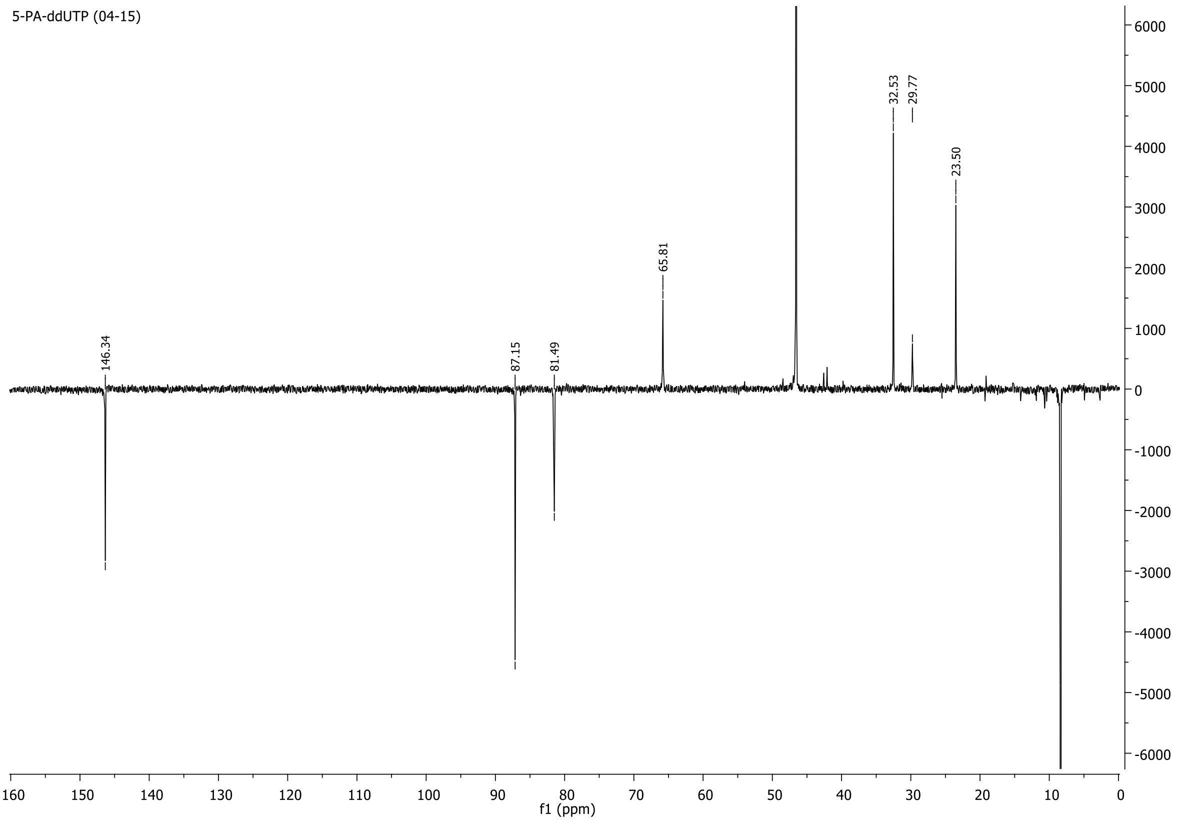

Supplementary Figure S46. ^{13}C Dept-135 spectra of **dd^{PA}UTP**

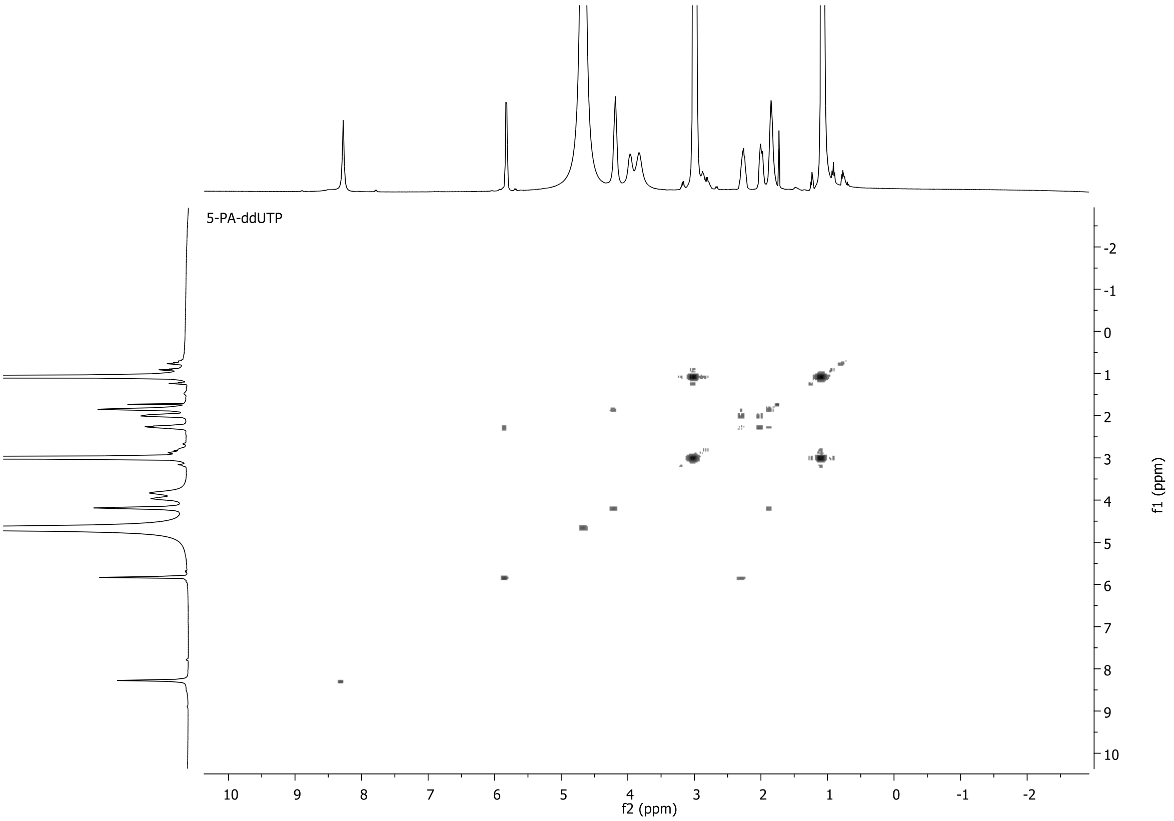

Supplementary Figure S47. H,H-COSY spectra of **dd^{PA}UTP**

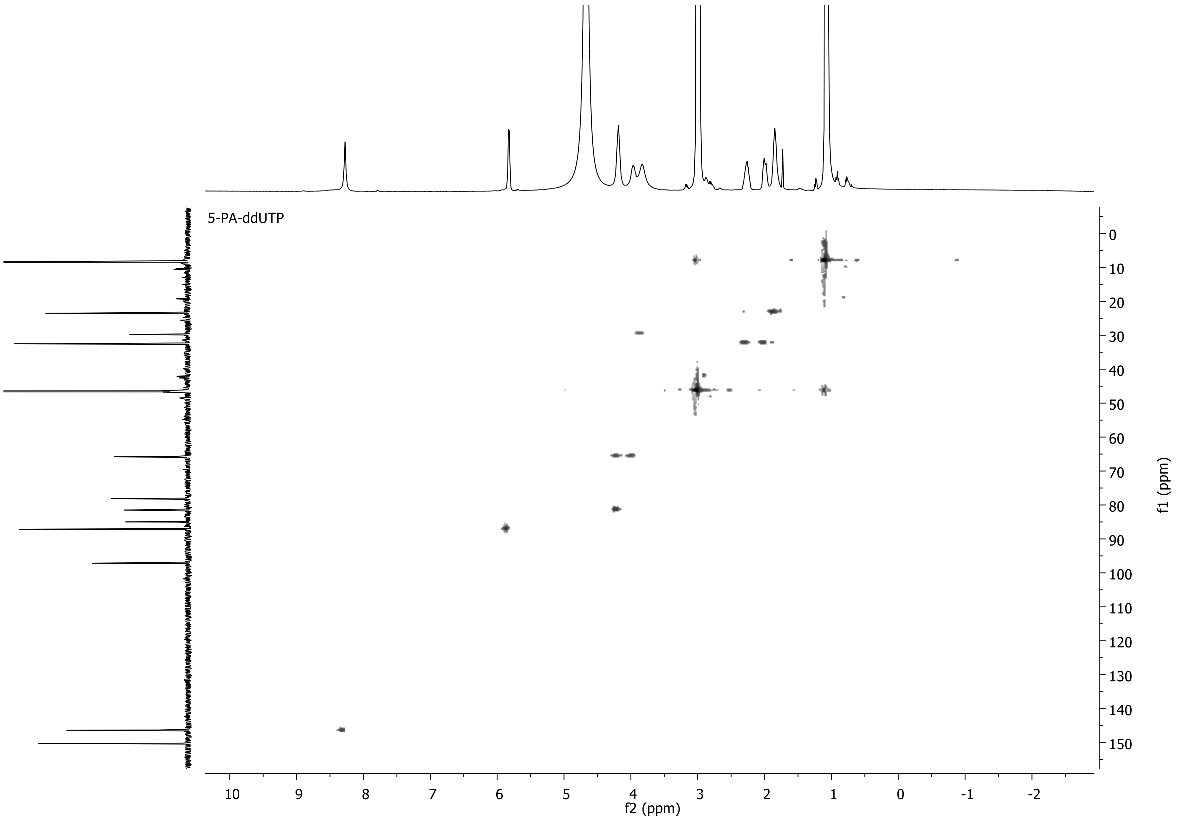

Supplementary Figure S48. H,C-HSQC spectra of **dd^{PA}UTP**

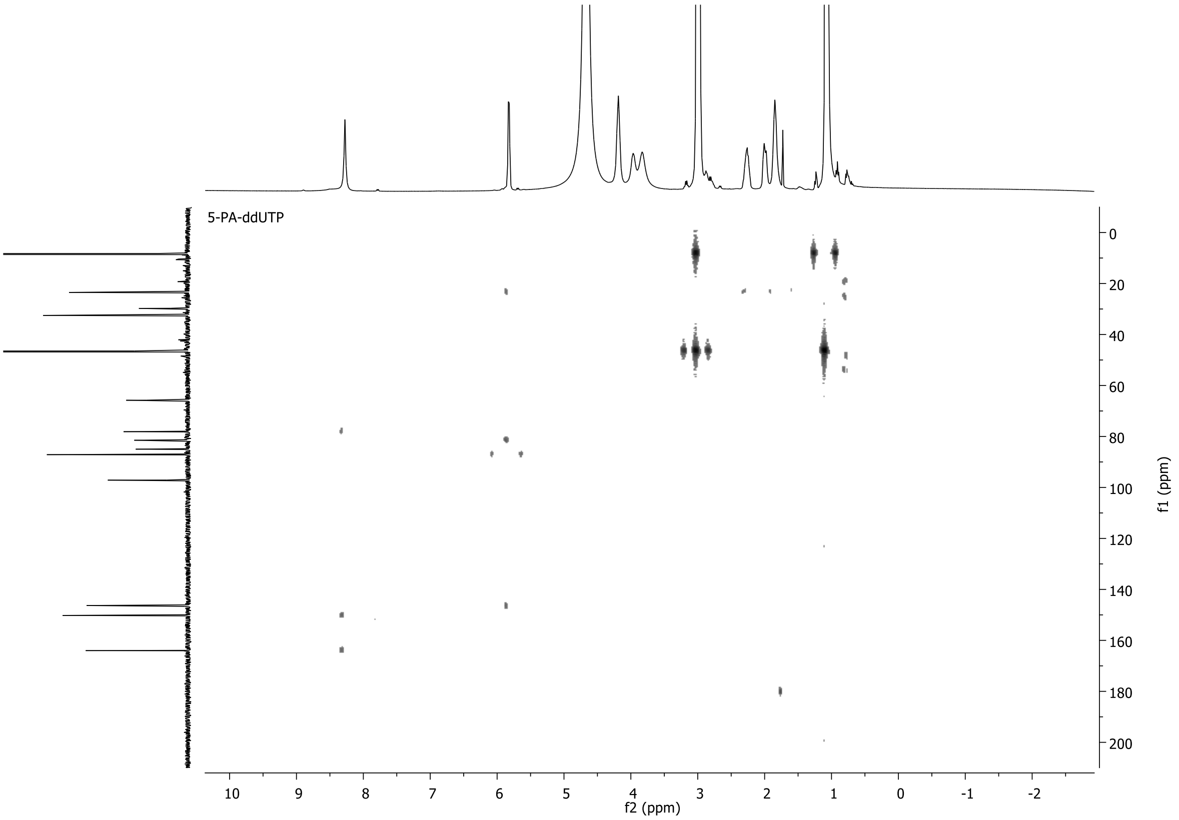

Supplementary Figure S49. H,C-HMBC spectra of **dd^{PA}UTP**

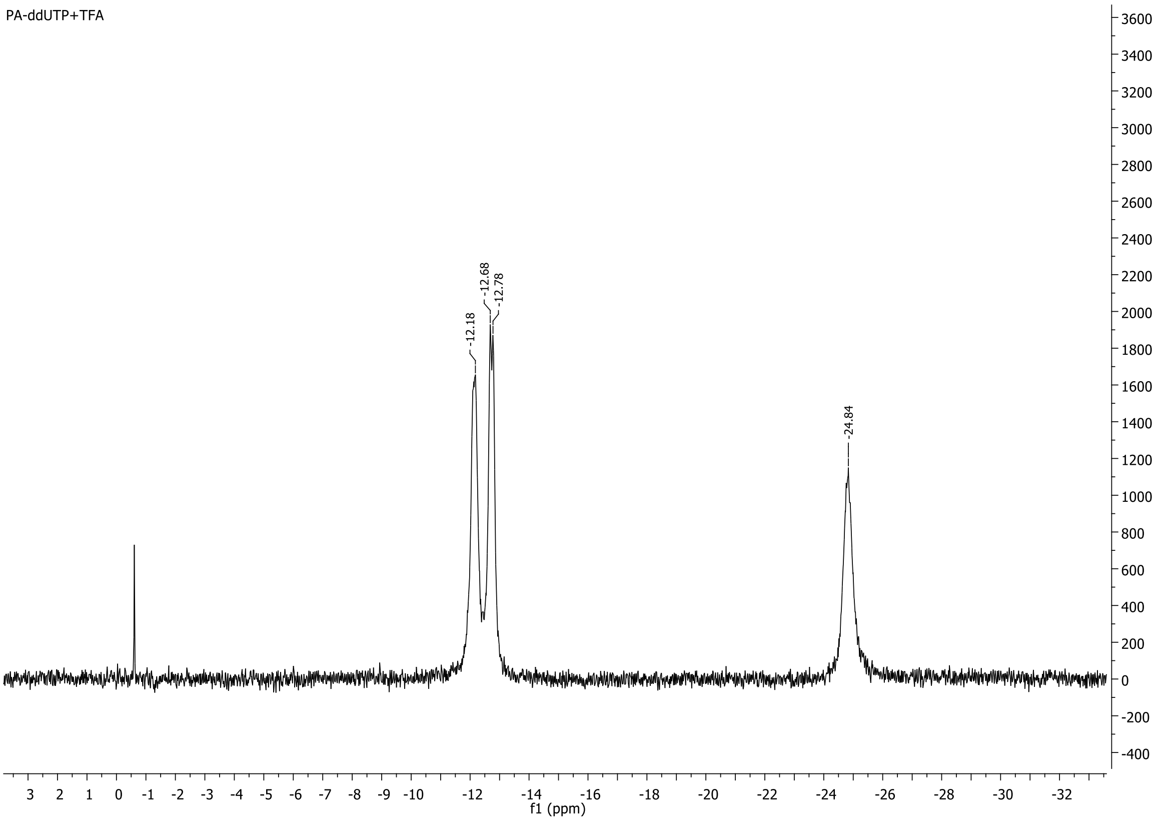

Supplementary Figure S50. ^{31}P spectra of **dd^{PA}UTP**

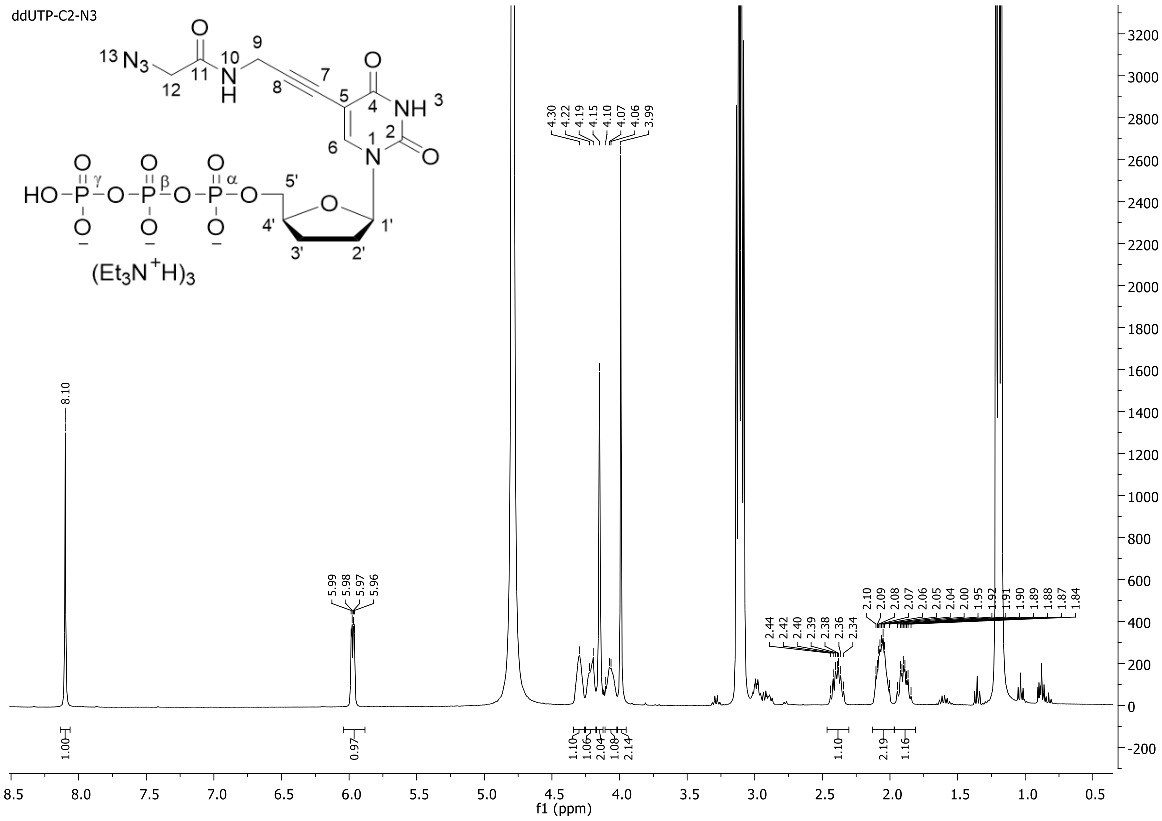

Supplementary Figure S51. ^{1}H spectra of **dd^{N3}UTP**

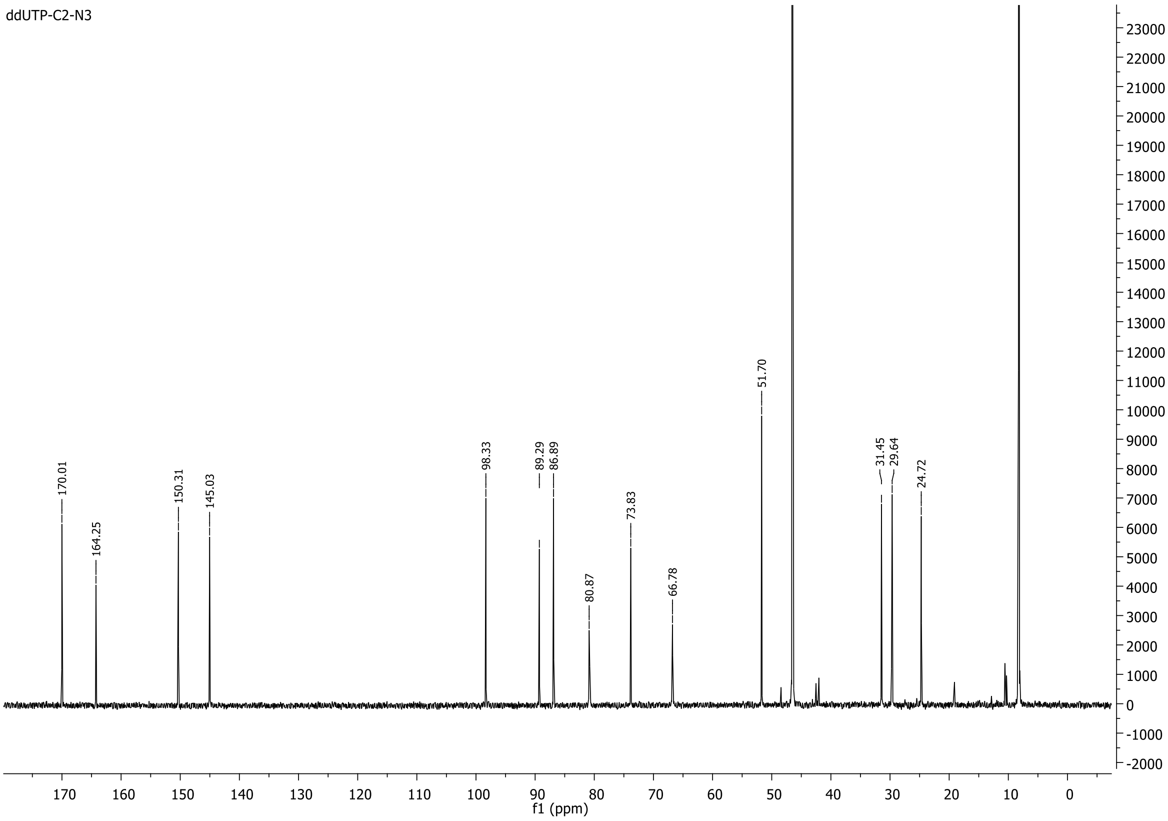

Supplementary Figure S52. ^{13}C spectra of **dd^{N3}UTP**

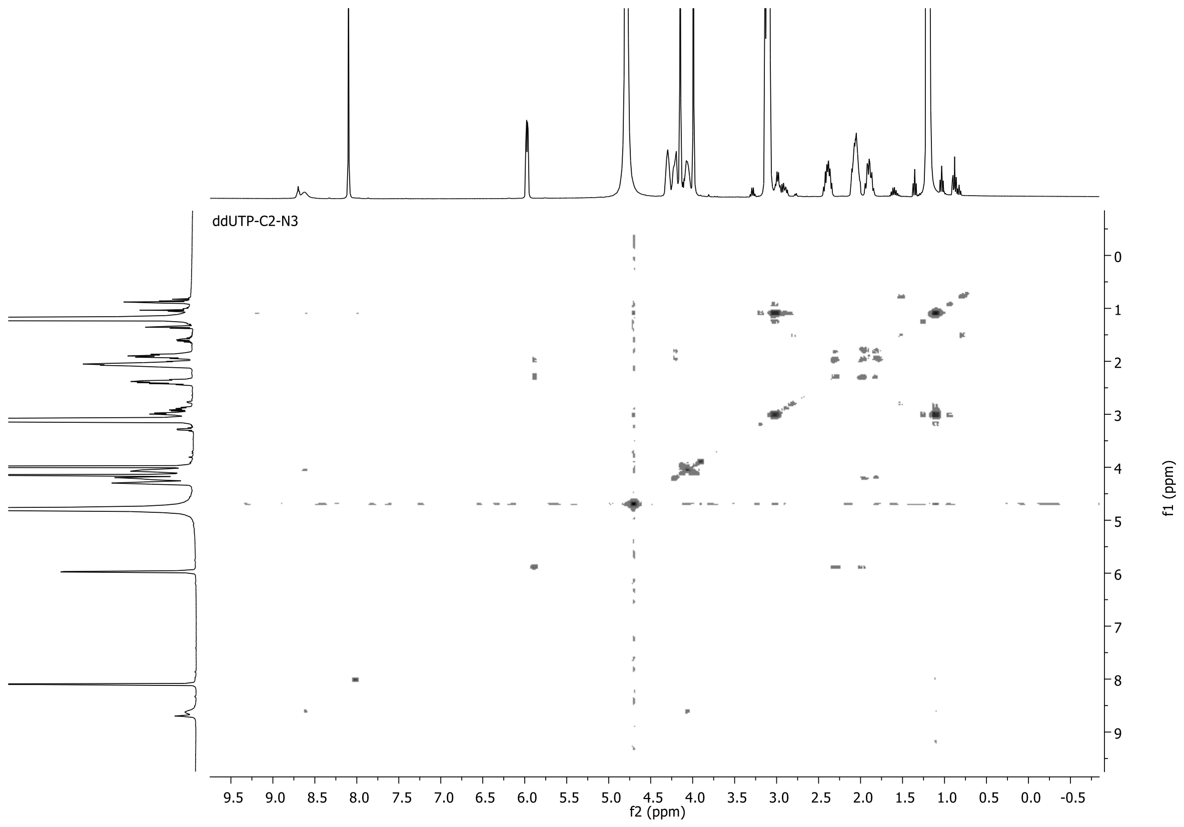

Supplementary Figure S53. H,H-COSY spectra of **dd^{N3}UTP**

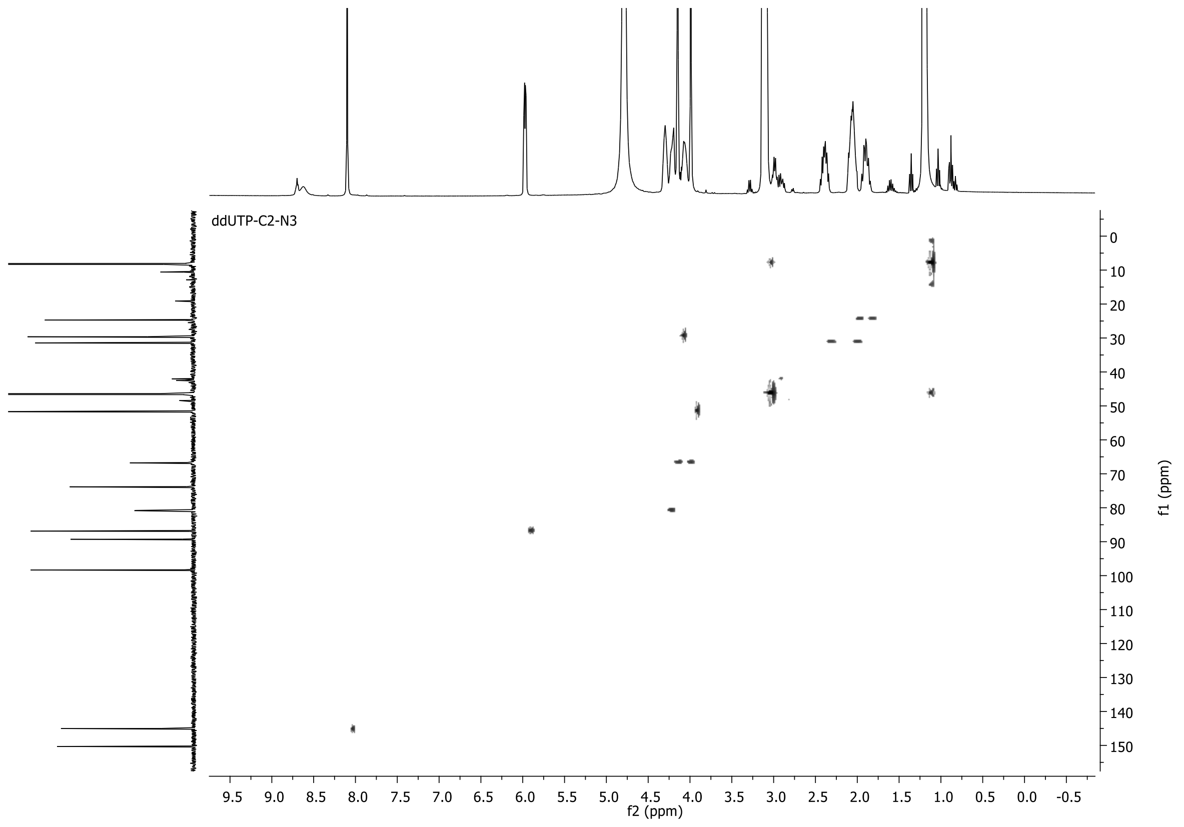

Supplementary Figure S54. H,C-HSQC spectra of **dd^{N3}UTP**

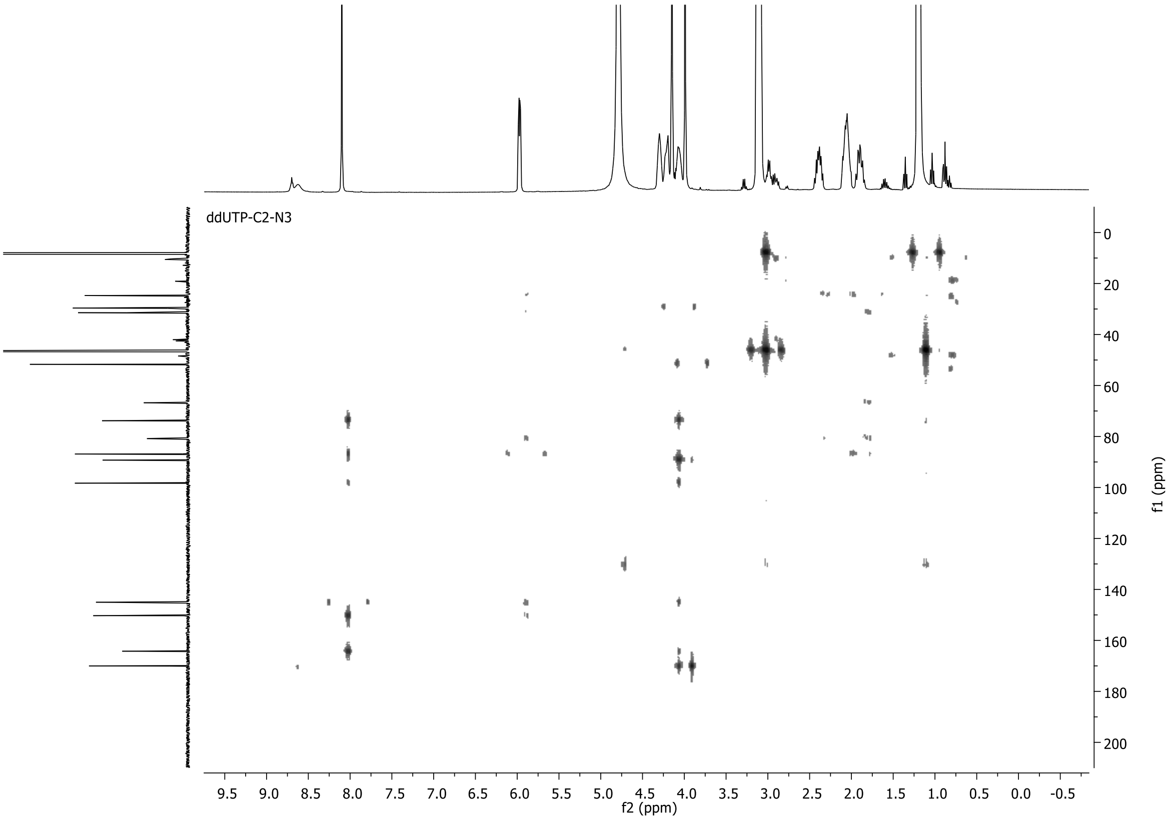

Supplementary Figure S55. H,C-HMBC spectra of **dd^{N3}UTP**

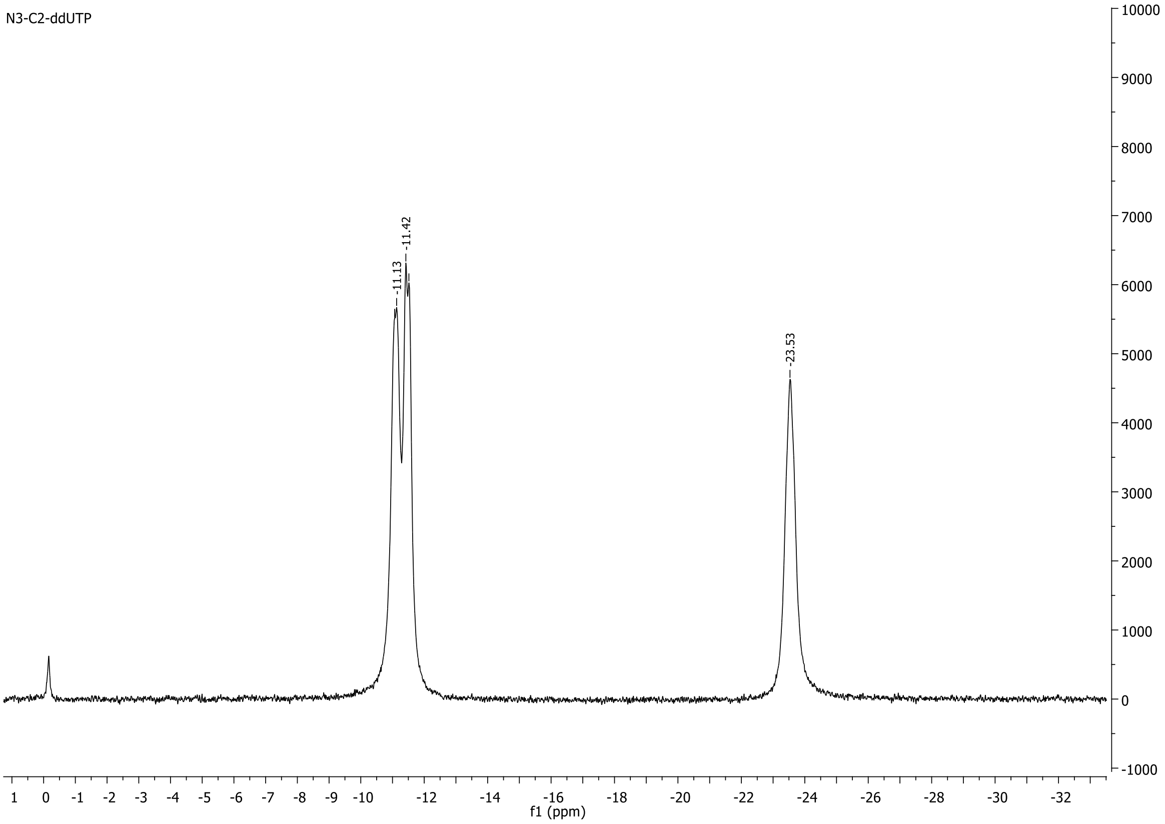

Supplementary Figure S56. ^{31}P spectra of **dd^{N3}UTP**

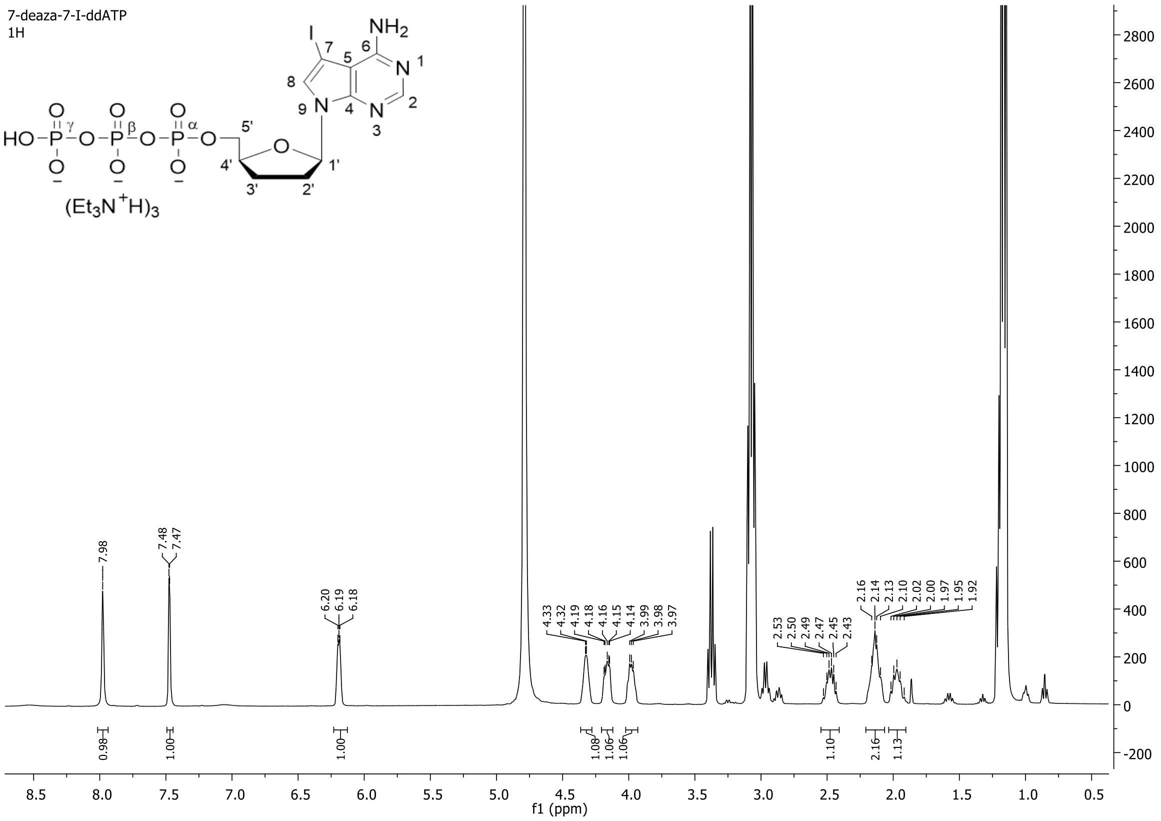

Supplementary Figure S57. ^{1}H spectra of **dd^{I}ATP**

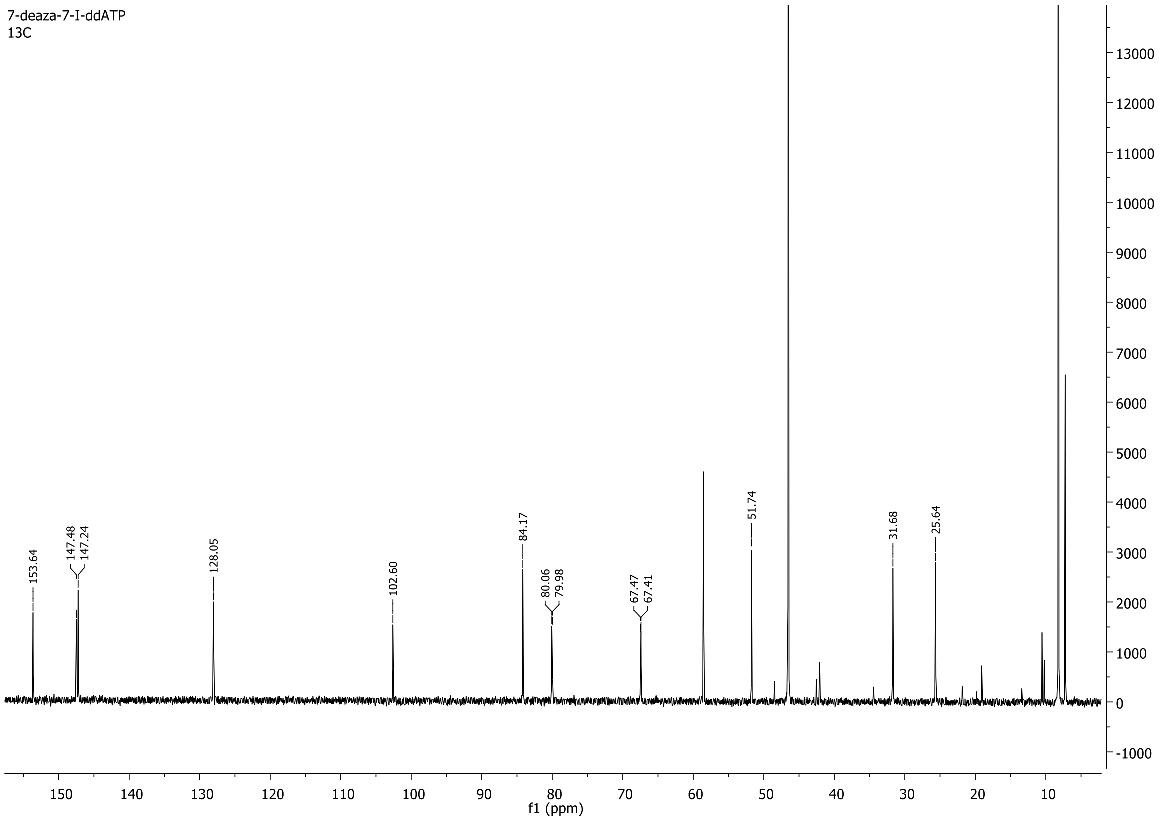

Supplementary Figure S58. ^{13}C spectra of **dd^{I}ATP**

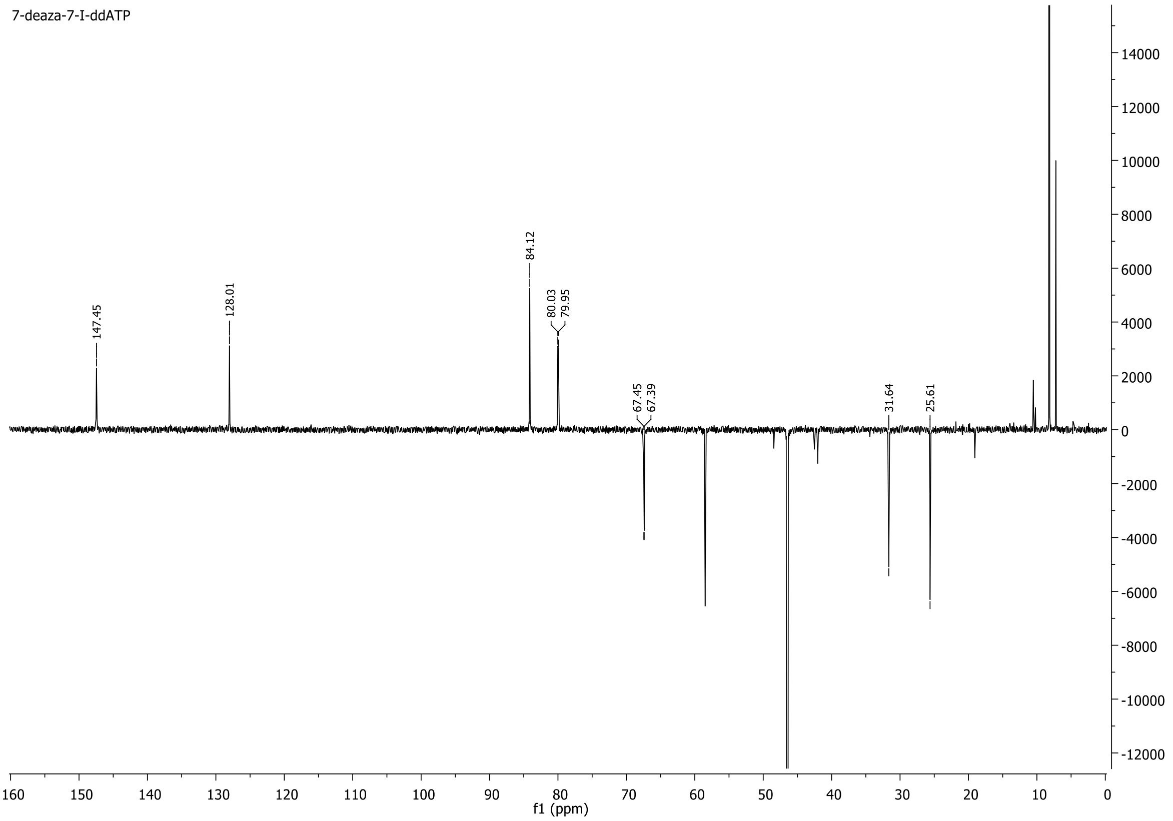

Supplementary Figure S59. ^{13}C Dept-135 spectra of **dd^{I}ATP**

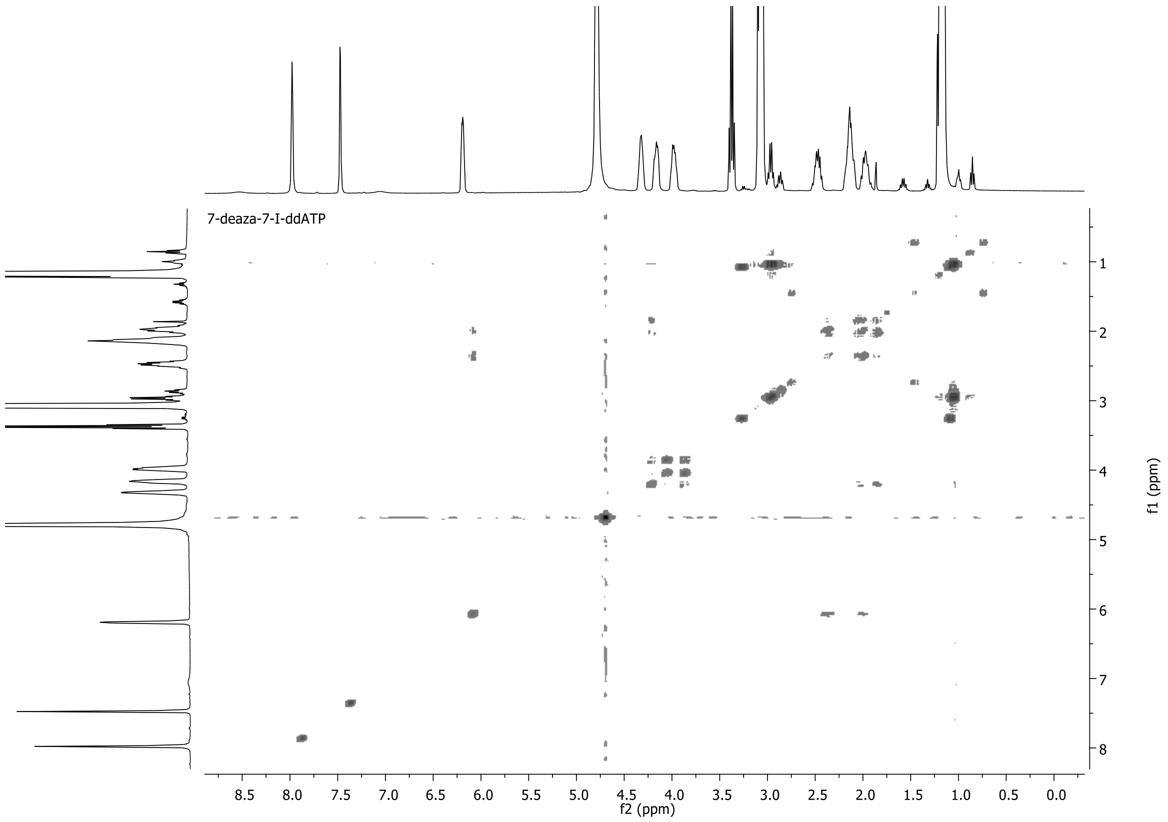

Supplementary Figure S60. H,H-COSY spectra of **dd^{I}ATP**

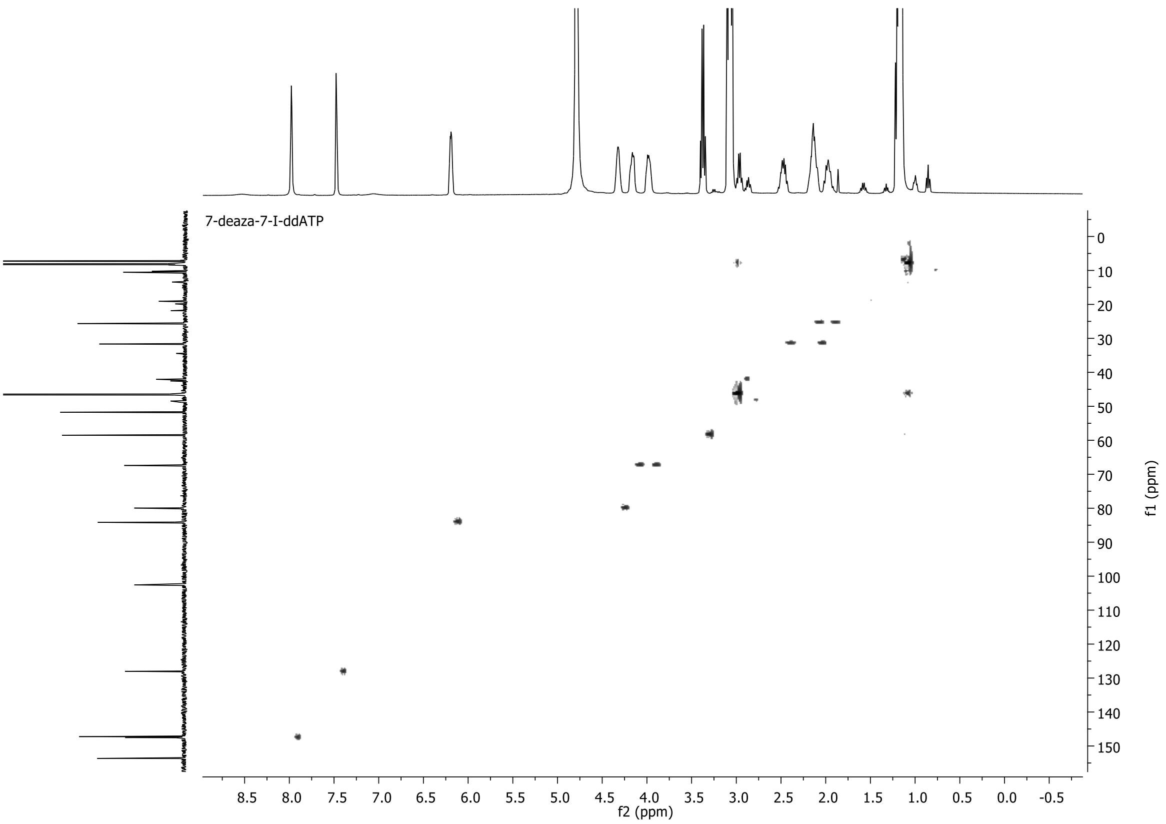

Supplementary Figure S61. H,C-HSQC spectra of **dd^{I}ATP**

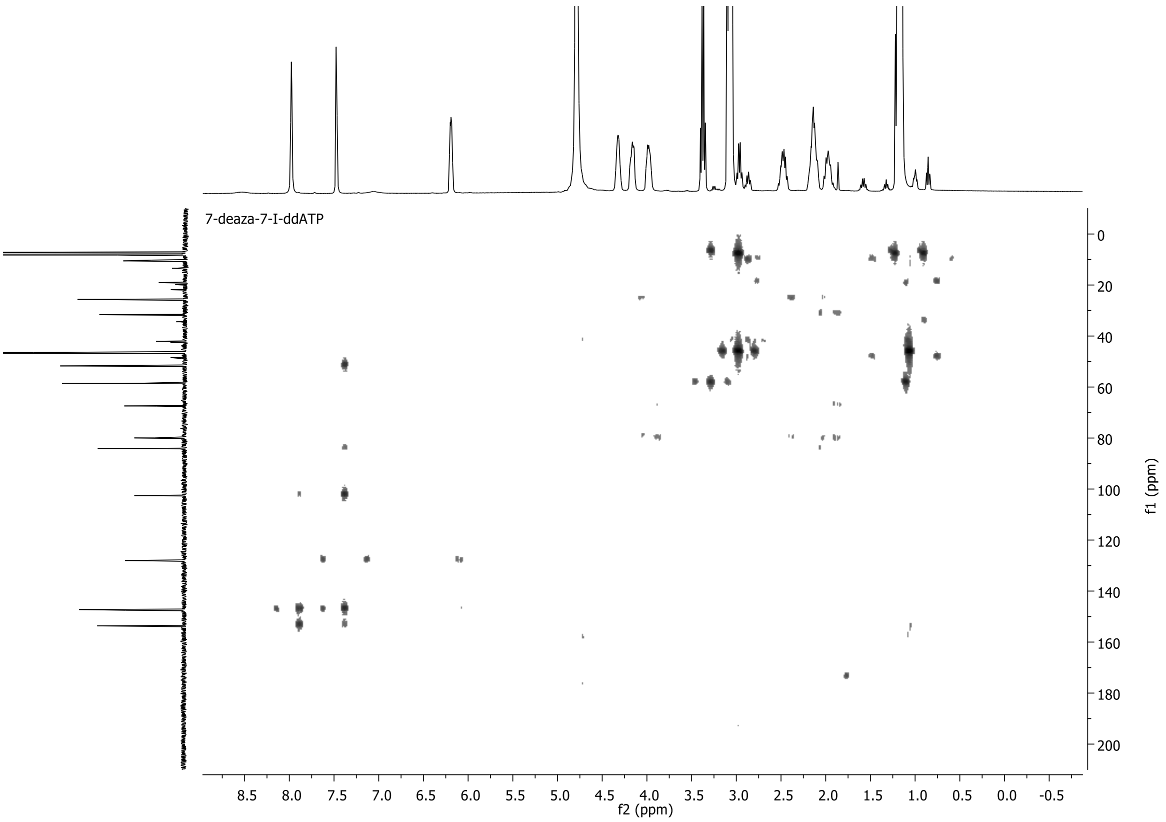

Supplementary Figure S62. H,C-HMBC spectra of **dd^{I}ATP**

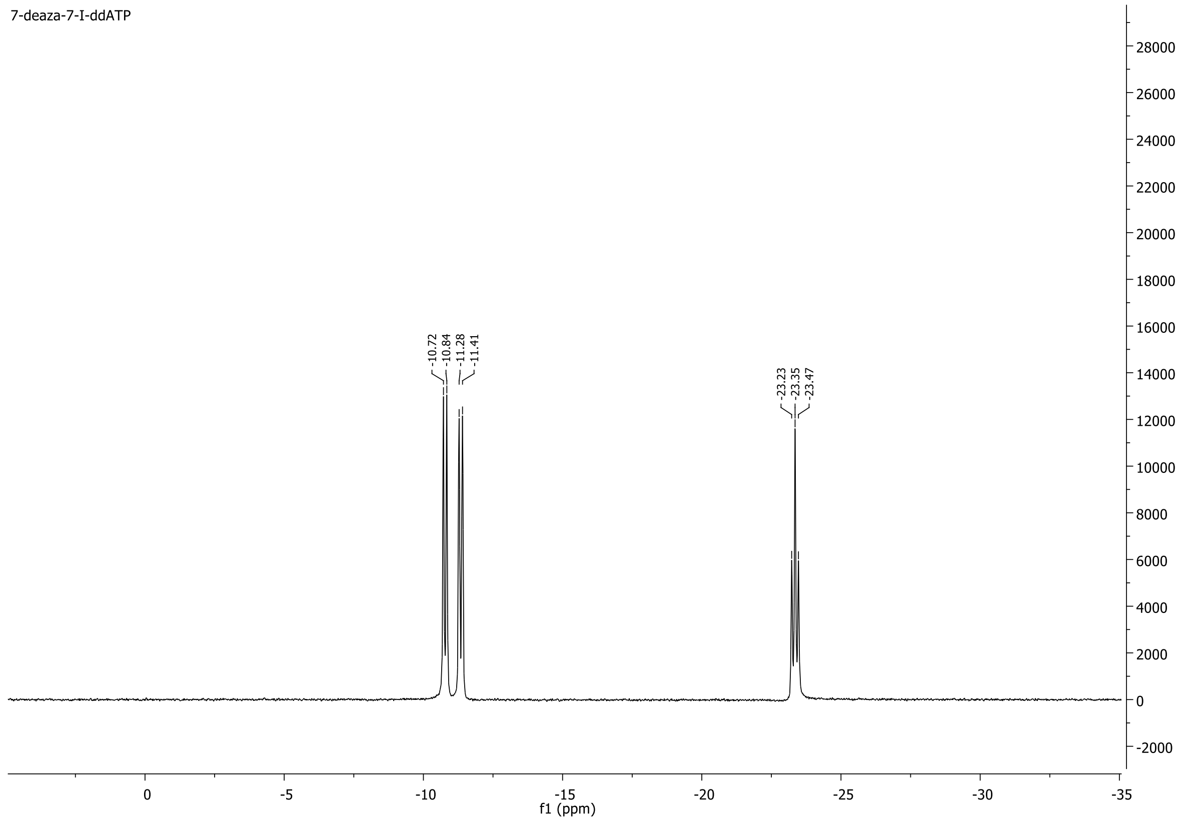

Supplementary Figure S63. ^{31}P spectra of **dd^{I}ATP**

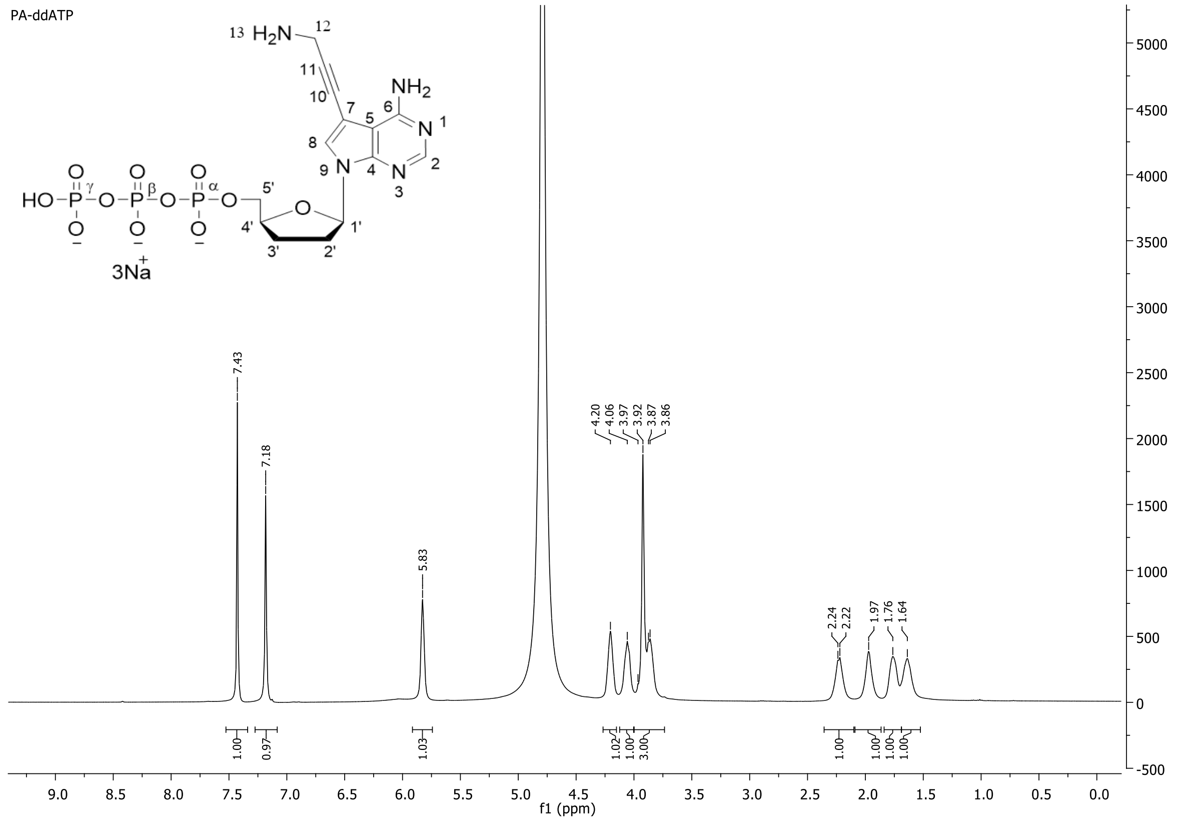

Supplementary Figure S64. ^{1}H spectra of **dd^{PA}ATP**

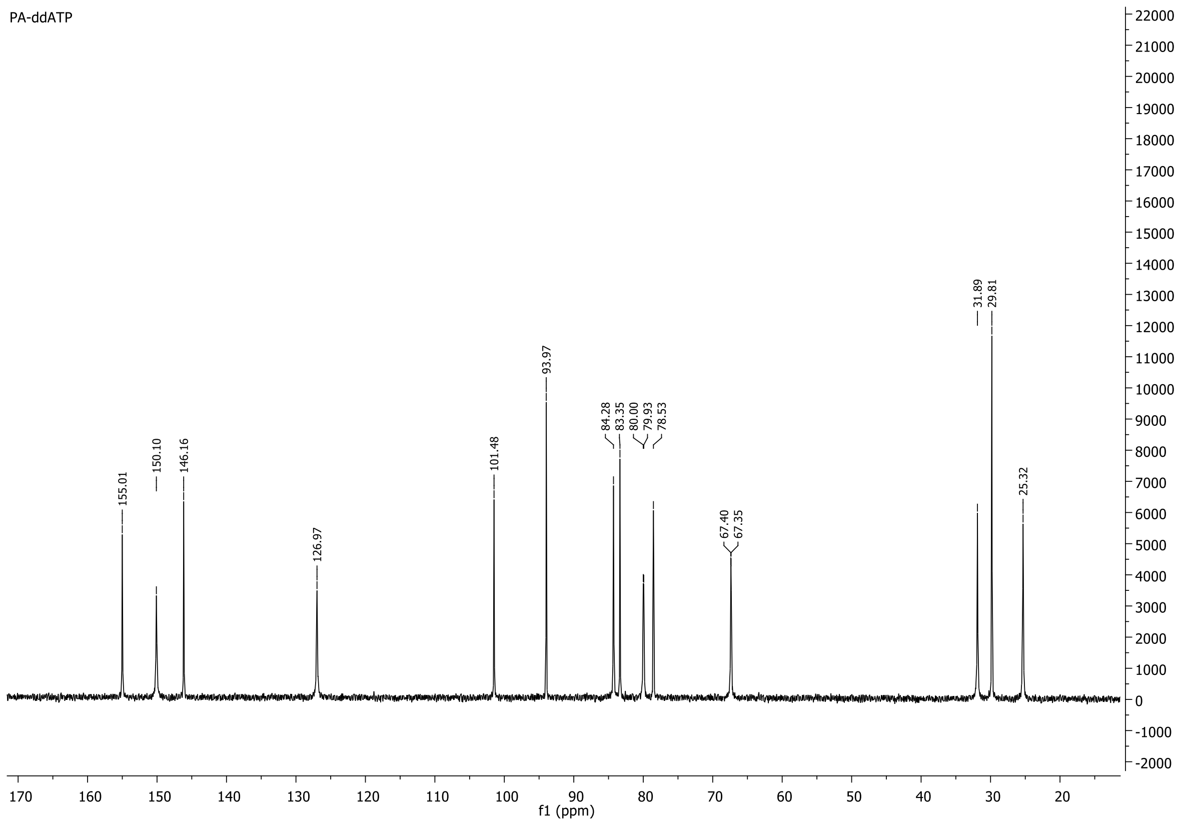

Supplementary Figure S65. ^{13}C spectra of **dd^{PA}ATP**

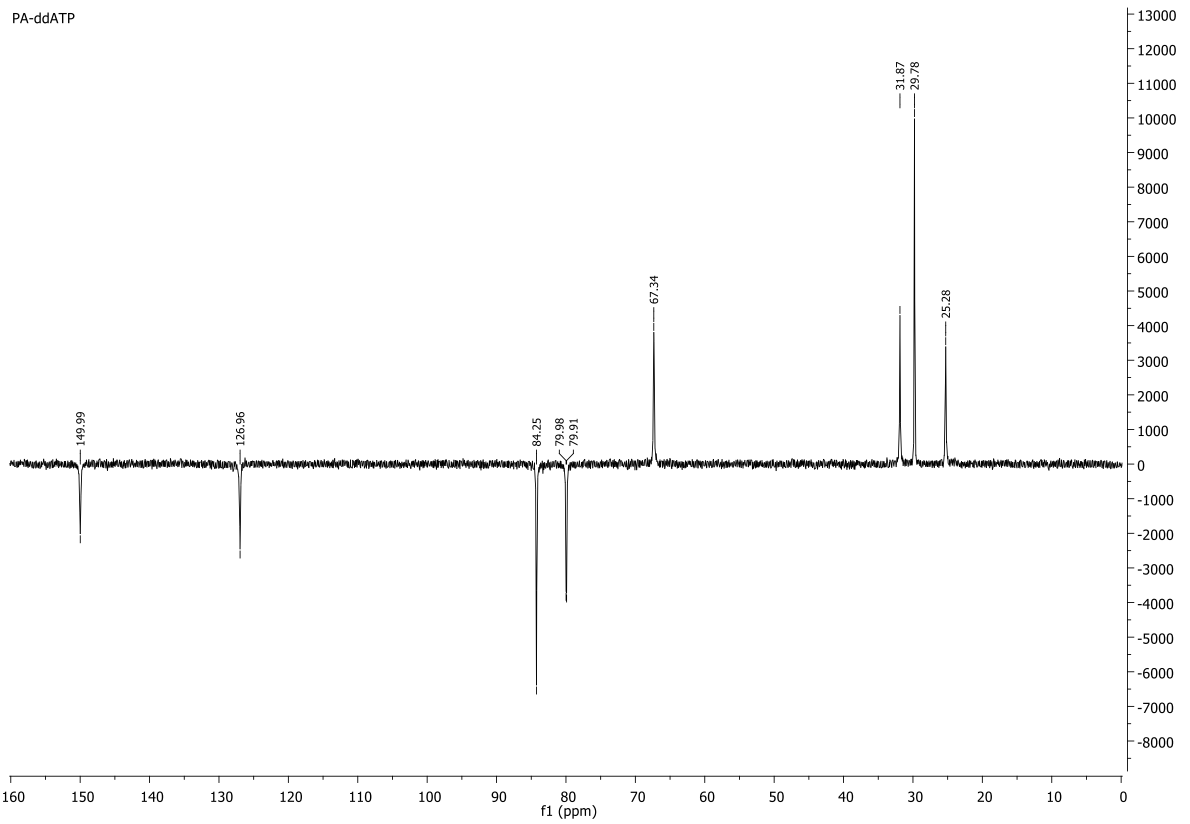

Supplementary Figure S66. ^{13}C Dept-135 spectra of **dd^{PA}ATP**

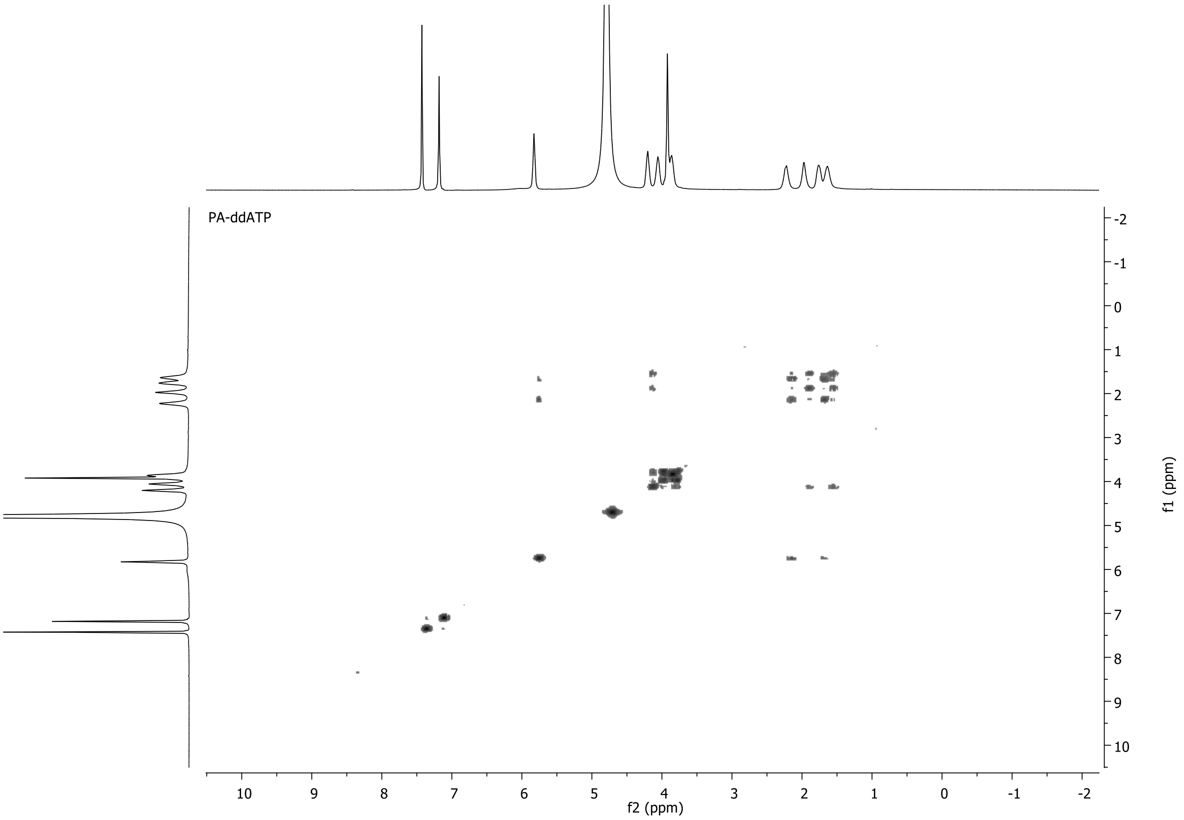

Supplementary Figure S67. H,H-COSY spectra of **dd^{PA}ATP**

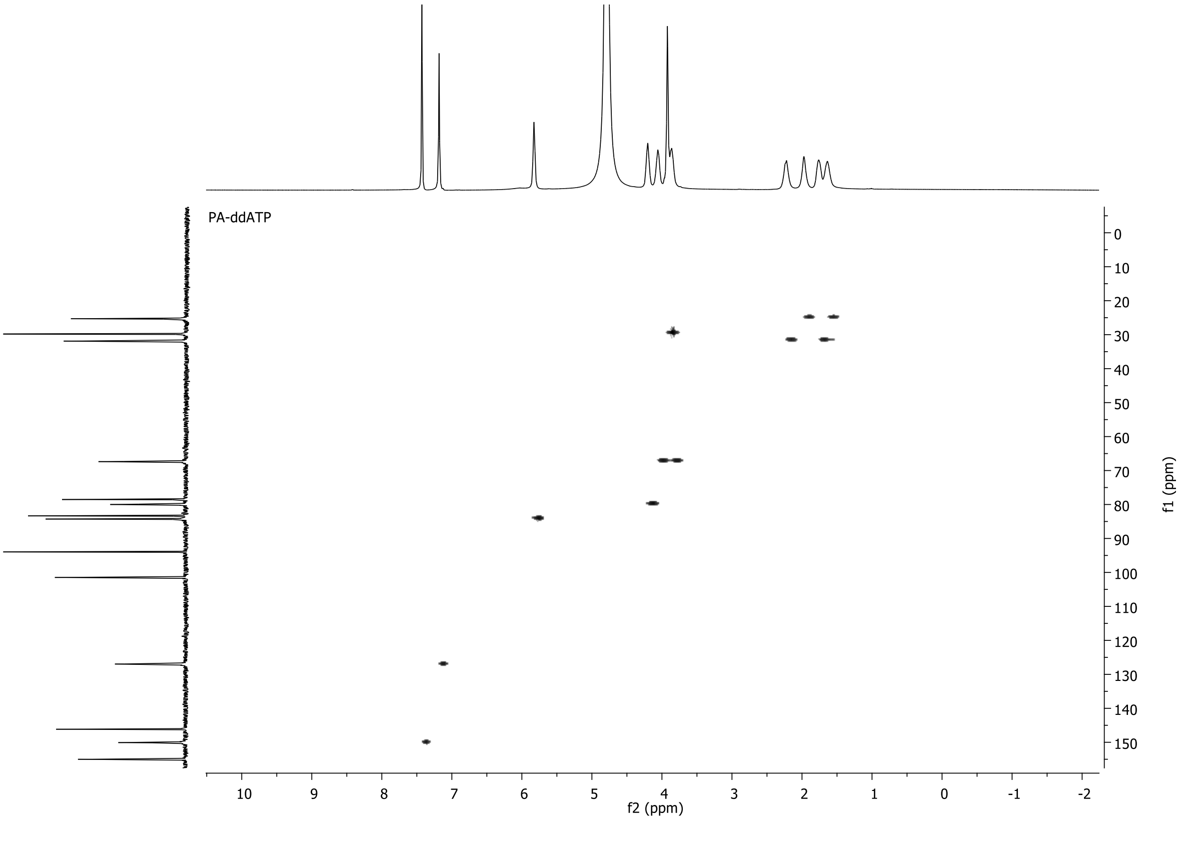

Supplementary Figure S68. H,C-HSQC spectra of **dd^{PA}ATP**

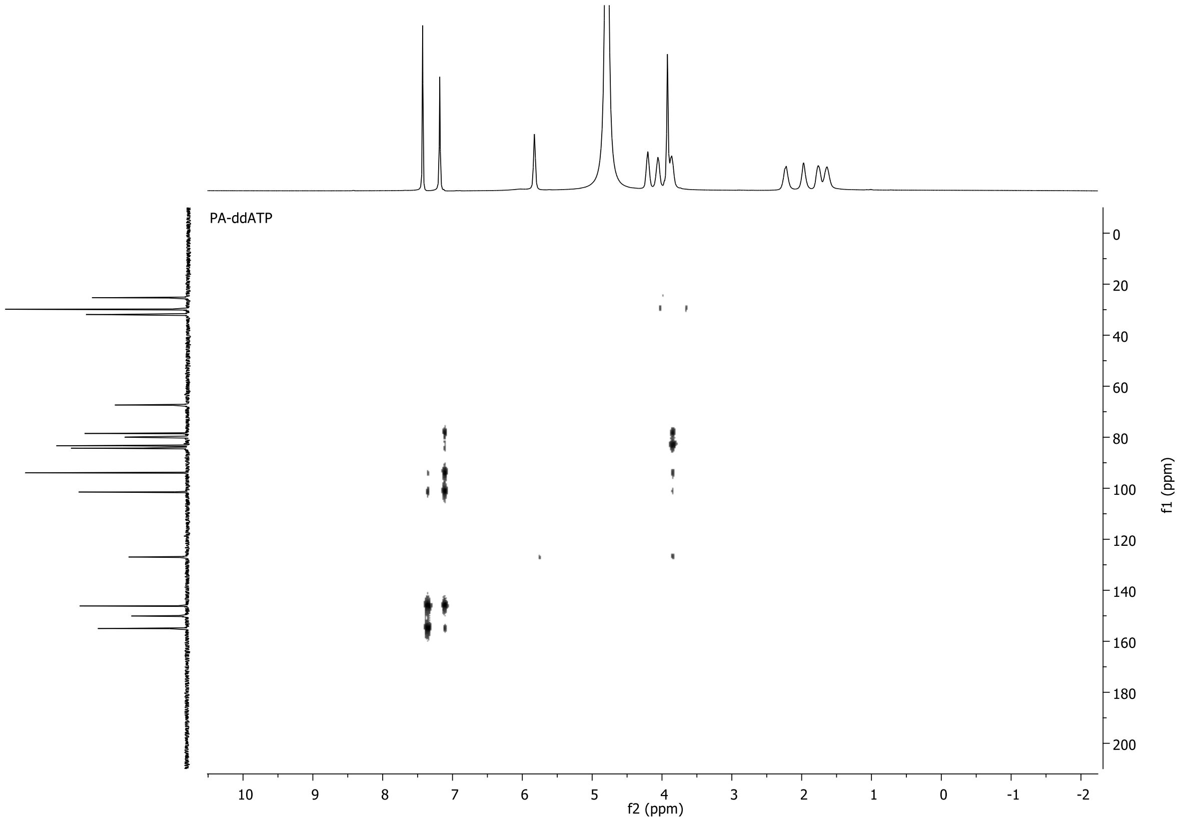

Supplementary Figure S69. H,C-HMBC spectra of **dd^{PA}ATP**

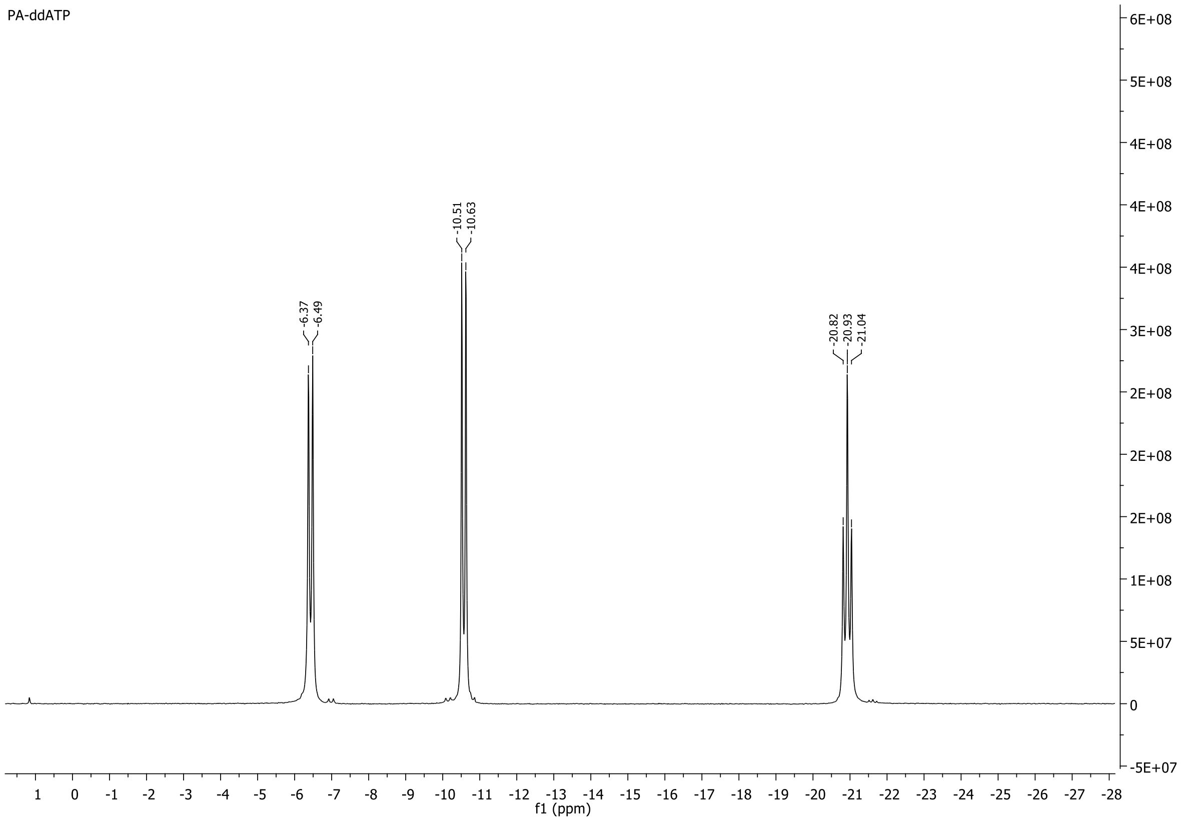

Supplementary Figure S70. ^{31}P spectra of **dd^{PA}ATP**

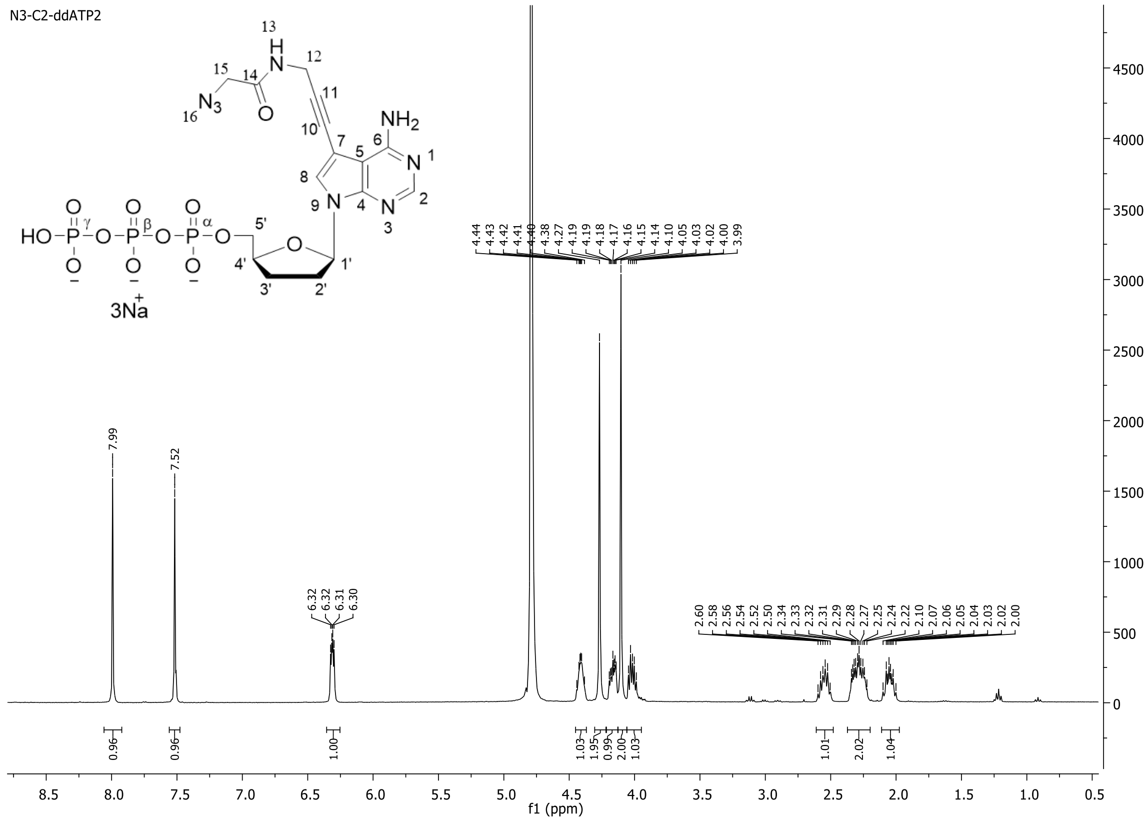

Supplementary Figure S71. ^{1}H spectra of **dd^{N3}ATP**

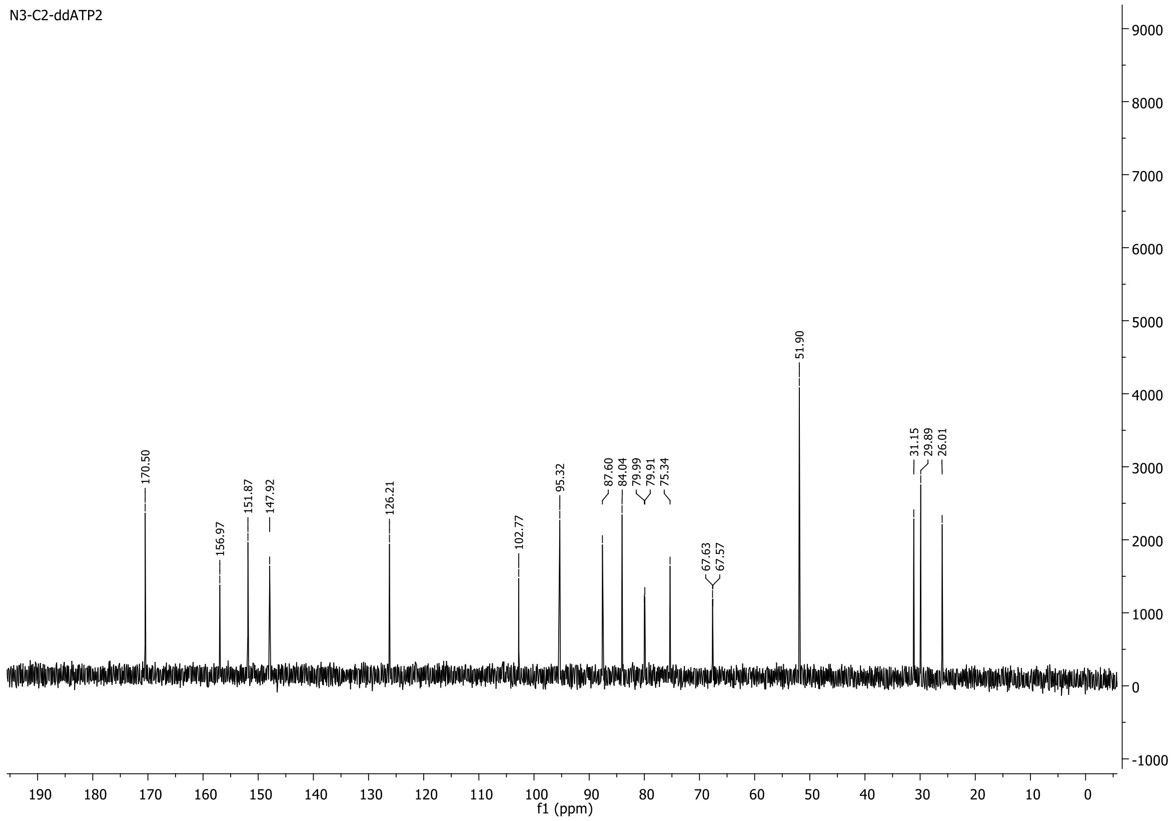

Supplementary Figure S72. ^{13}C spectra of **dd^{N3}ATP**

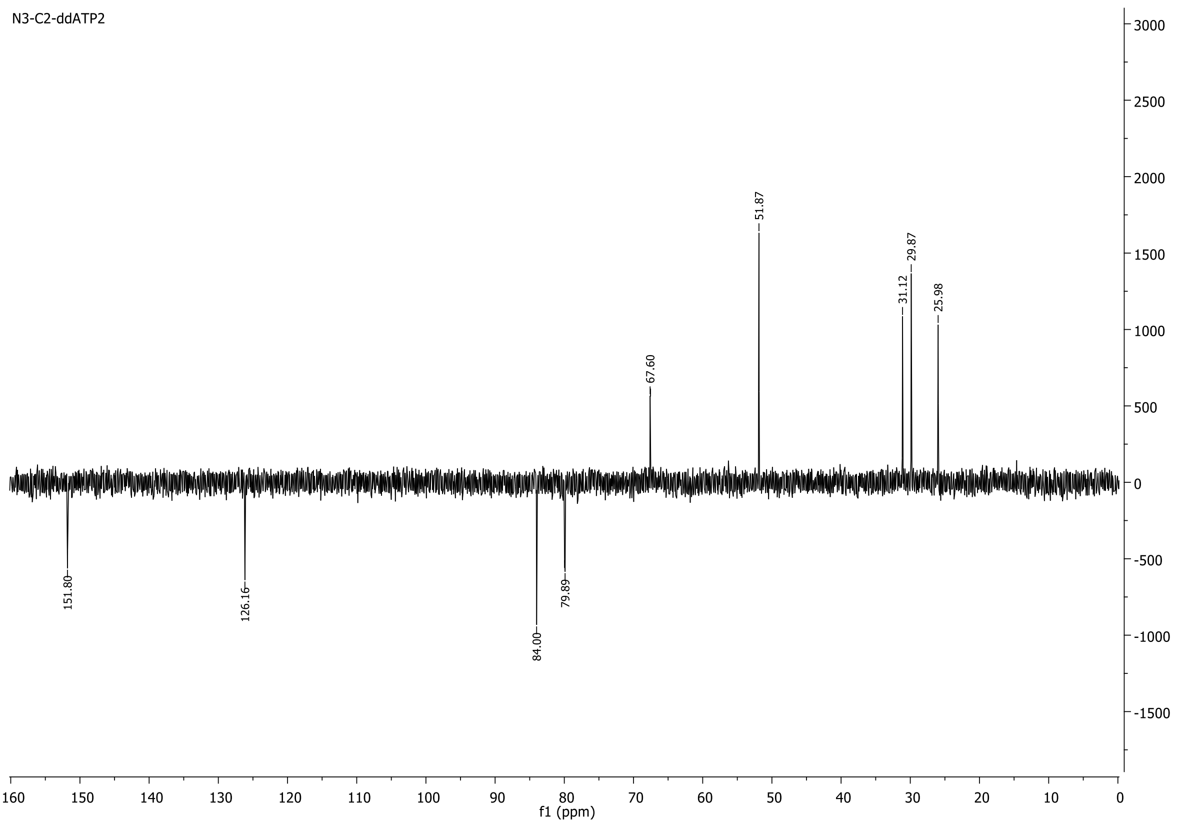

Supplementary Figure S73. ^{13}C Dept-135 spectra of **dd^{N3}ATP**

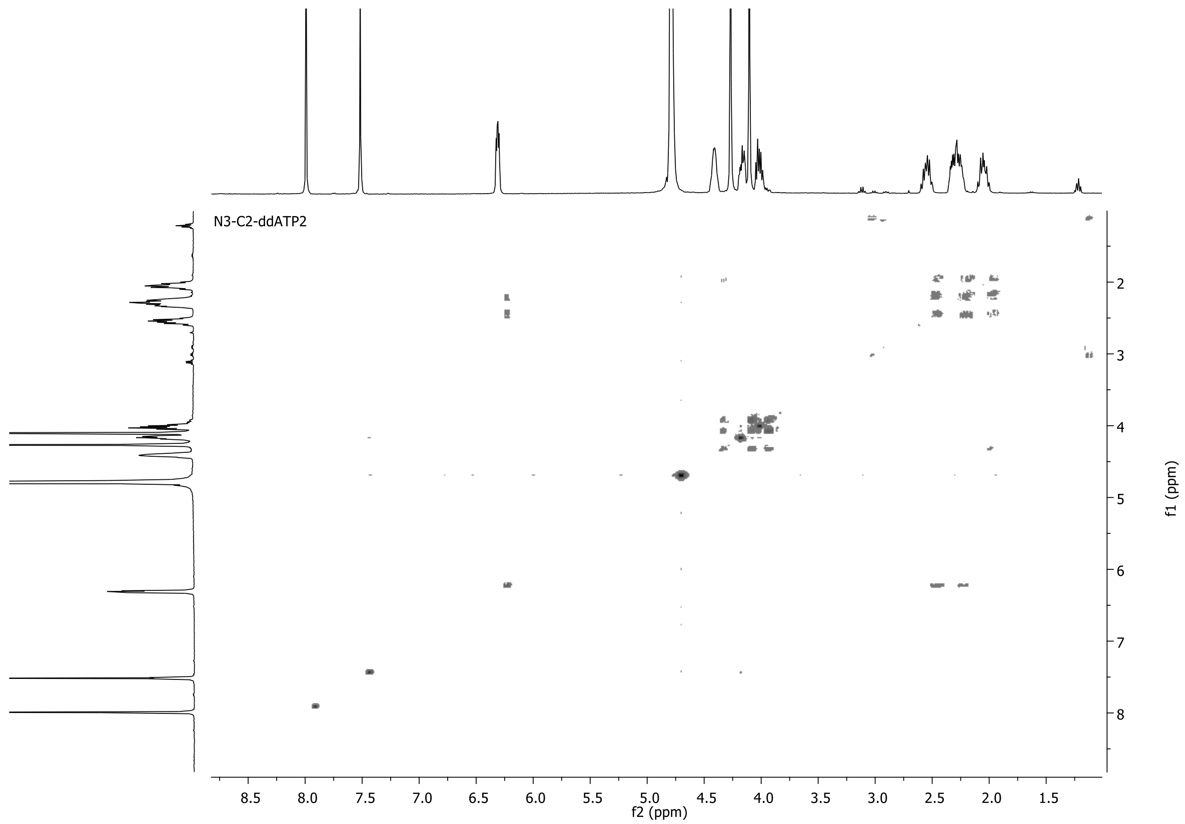

Supplementary Figure S74. H,H-COSY spectra of **dd^{N3}ATP**

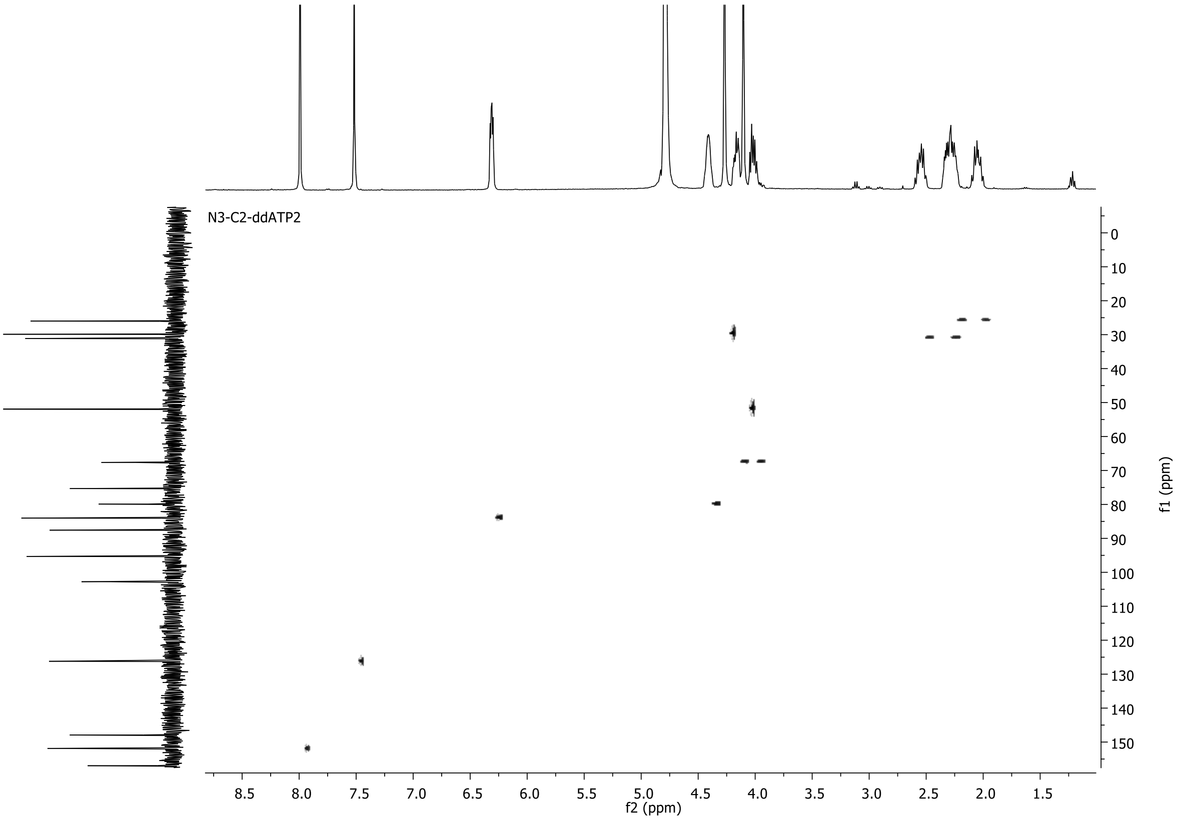

Supplementary Figure S75. H,C-HSQC spectra of **dd^{N3}ATP**

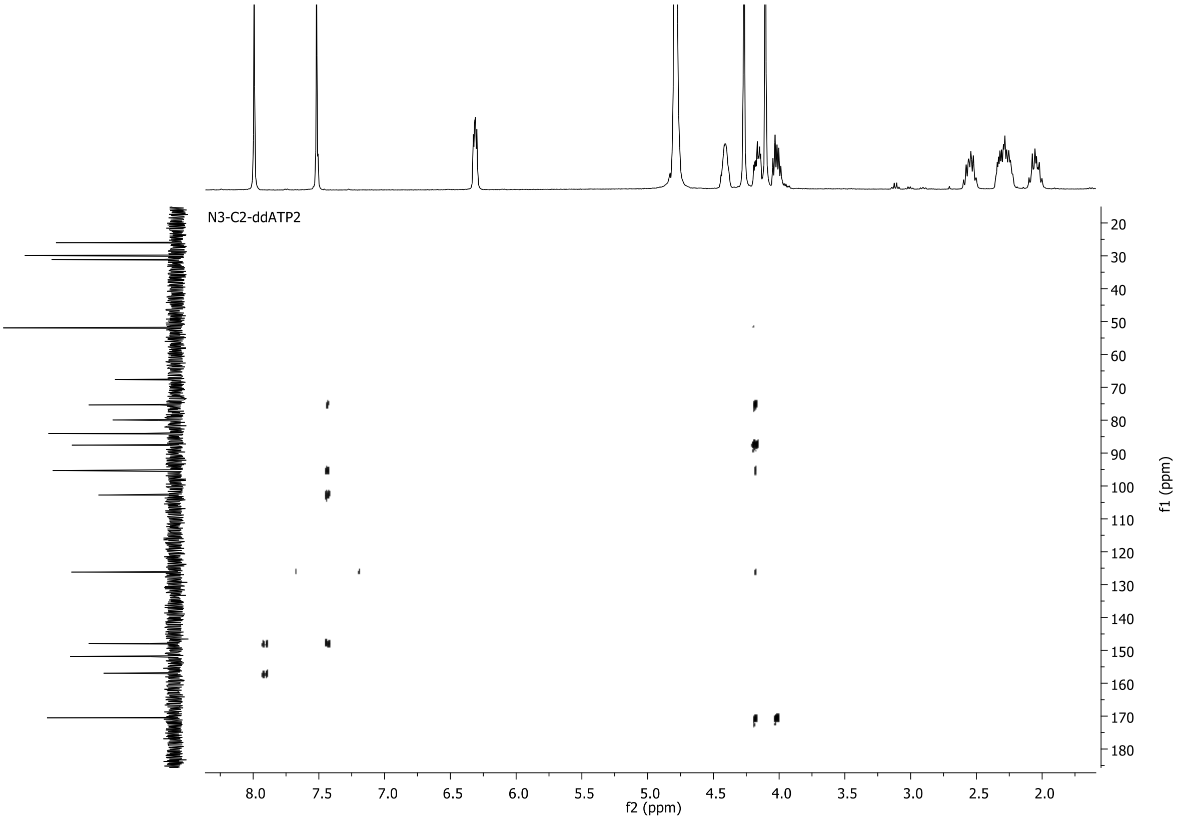

Supplementary Figure S76. H,C-HMBC spectra of **dd^{N3}ATP**

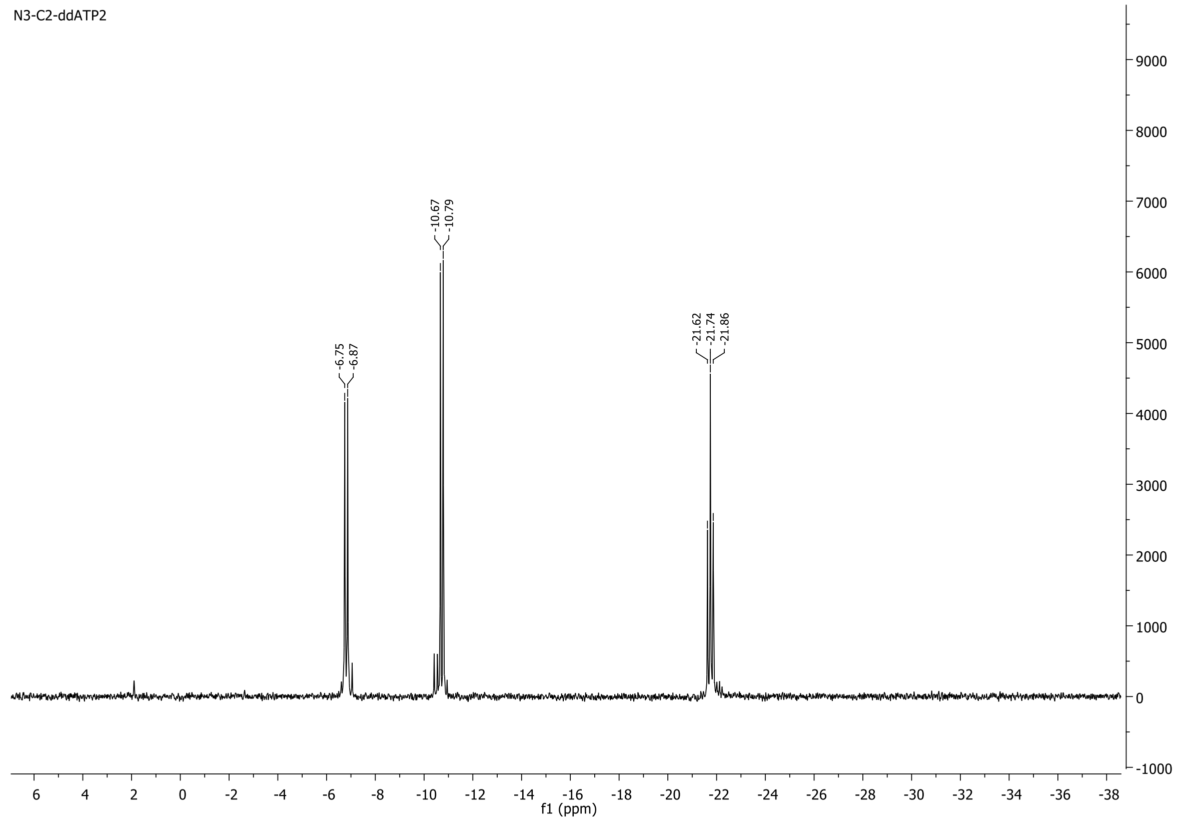

Supplementary Figure S77. ^{31}P spectra of **dd^{N3}ATP**

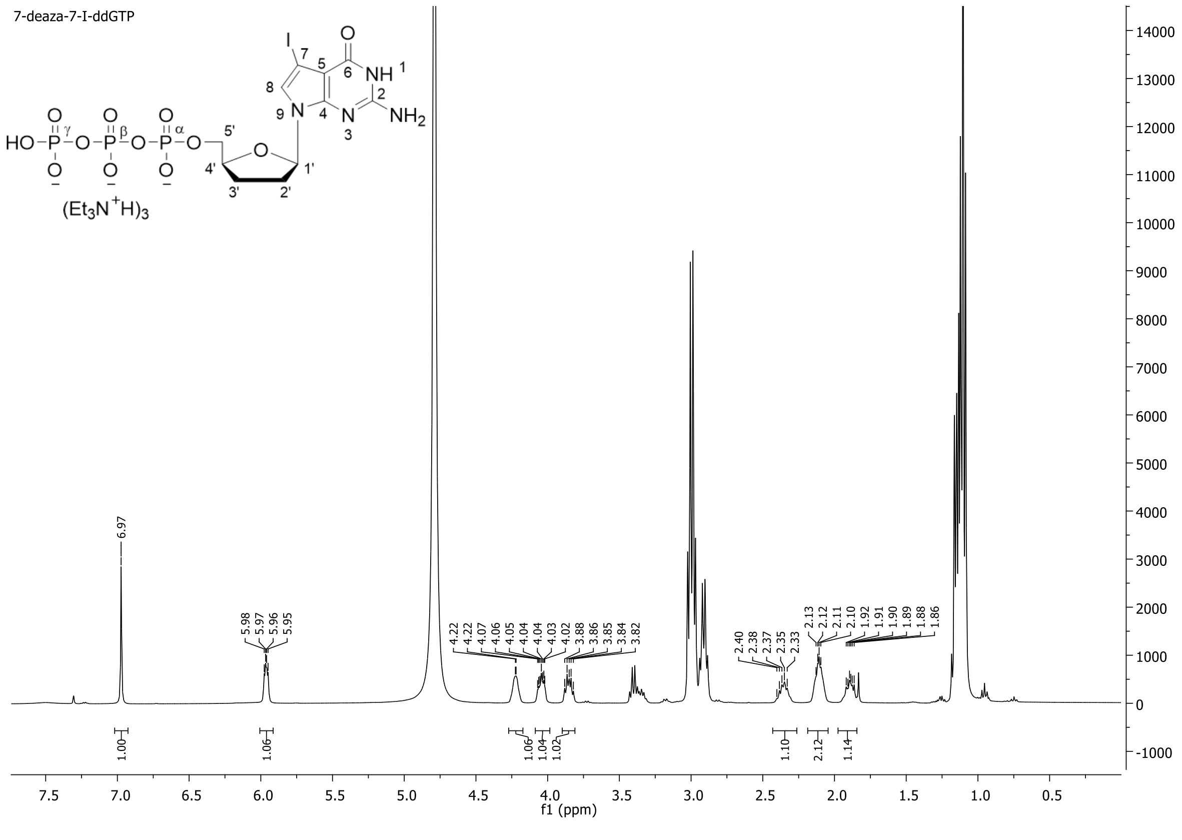

Supplementary Figure S78. ^{1}H spectra of **dd^{I}GTP**

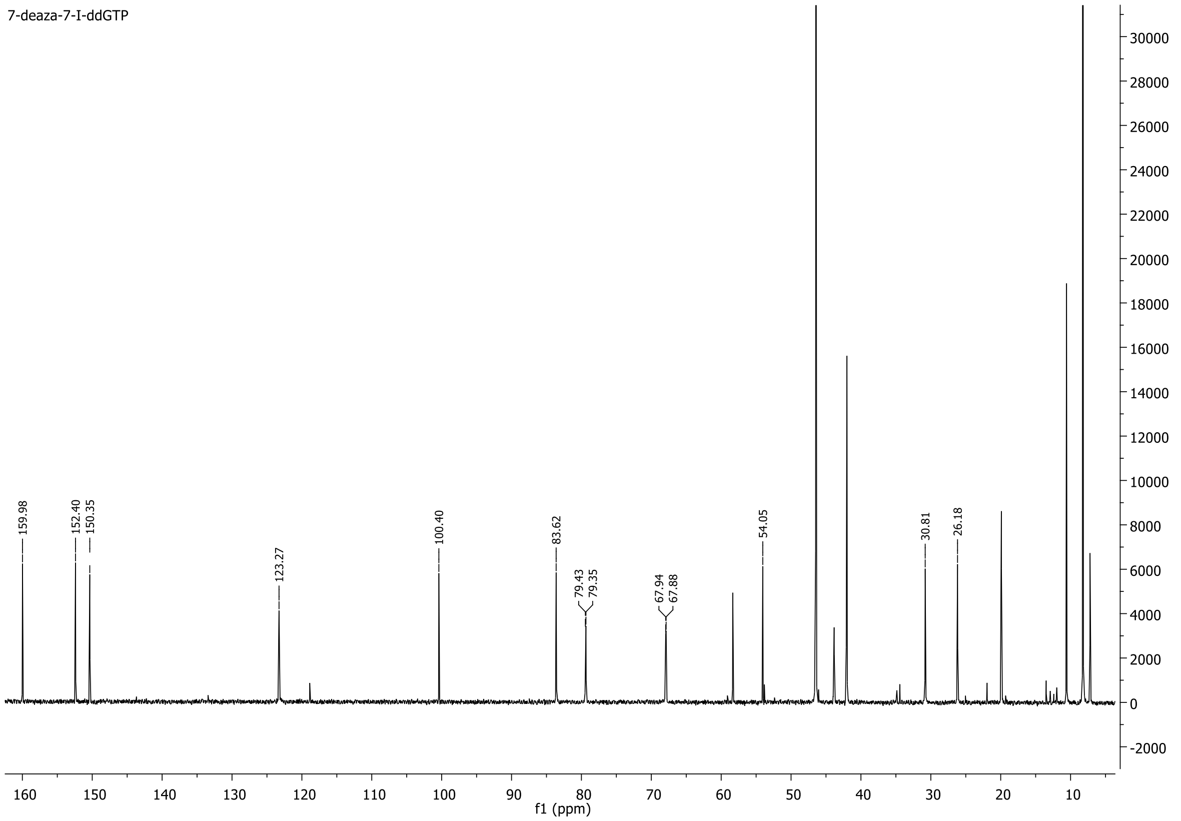

Supplementary Figure S79. ^{13}C spectra of **dd^{I}GTP**

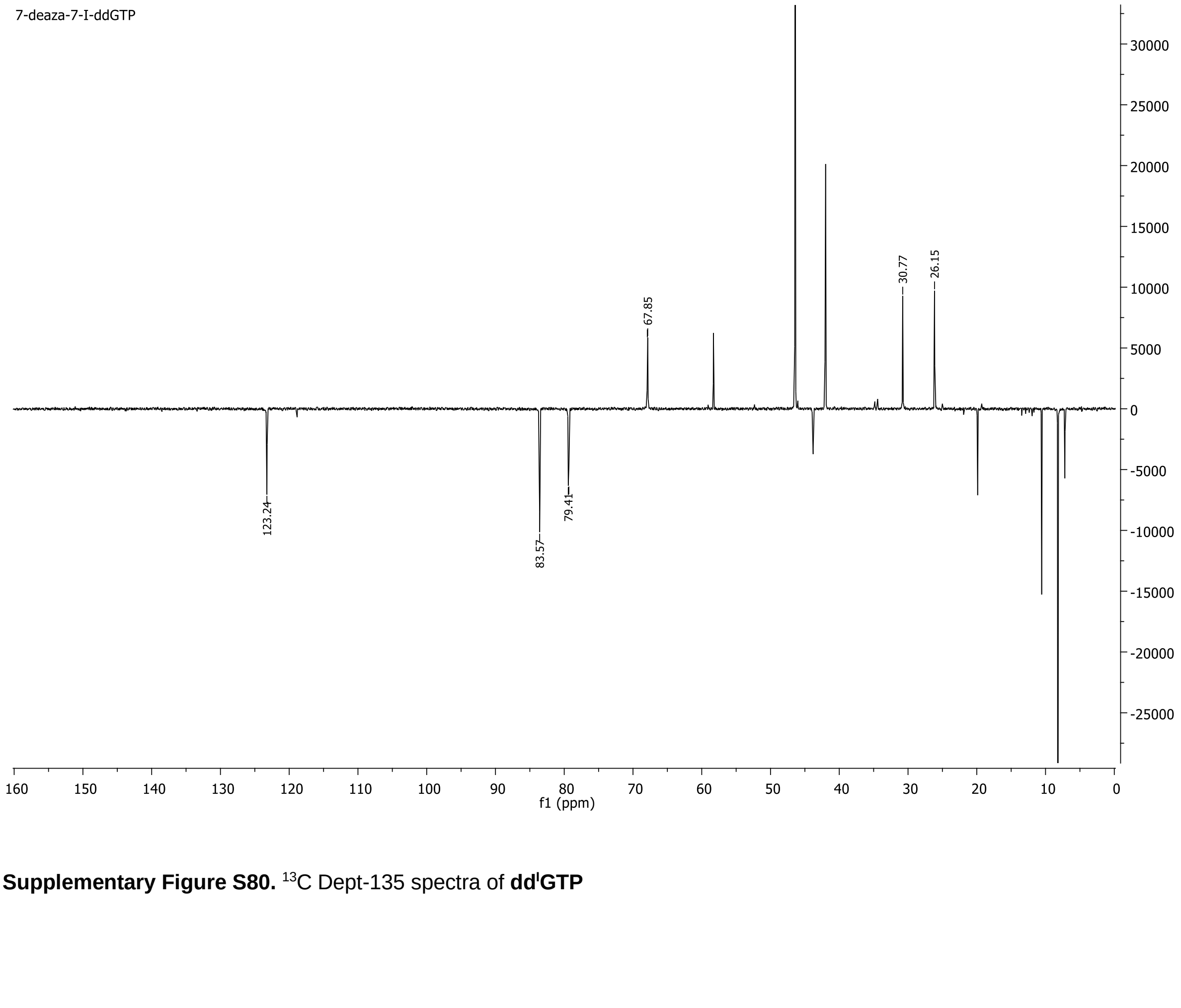

Supplementary Figure S80. ^{13}C Dept-135 spectra of **dd^{I}GTP**

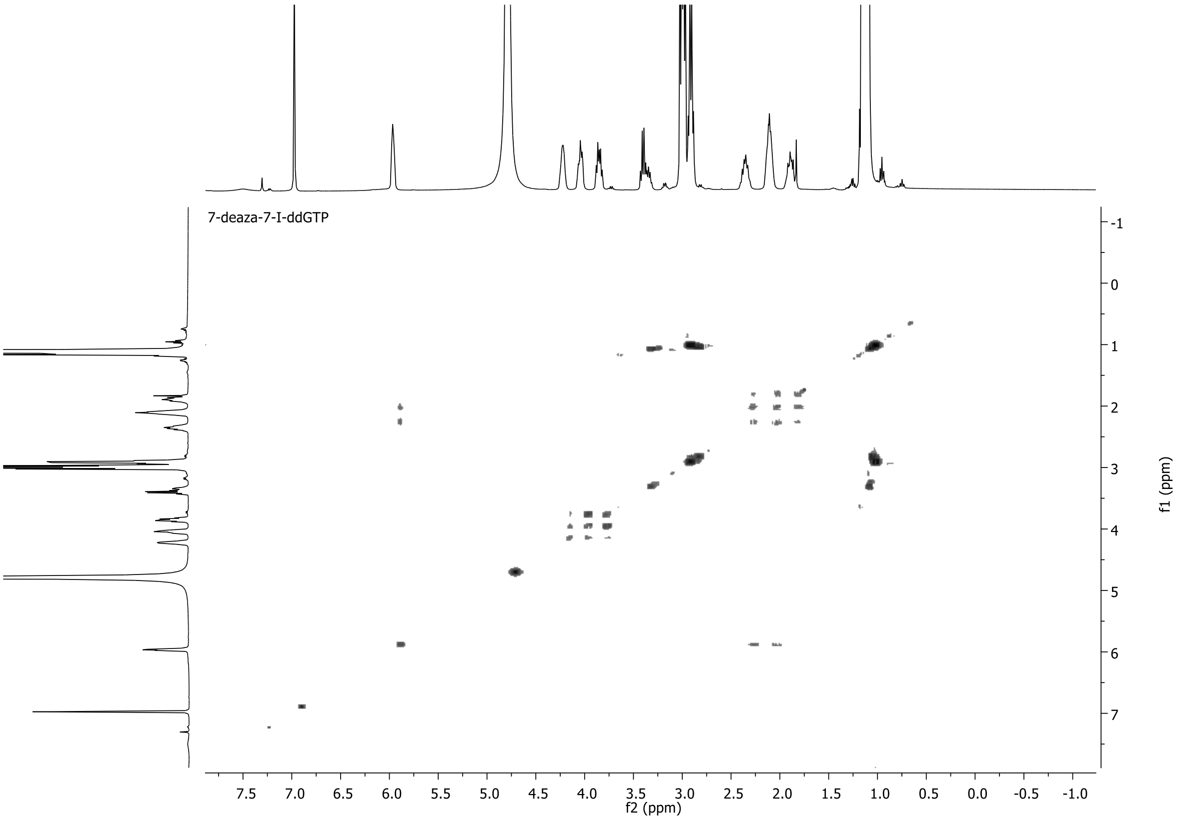

Supplementary Figure S81. H,H-COSY spectra of **dd[I]GTP**

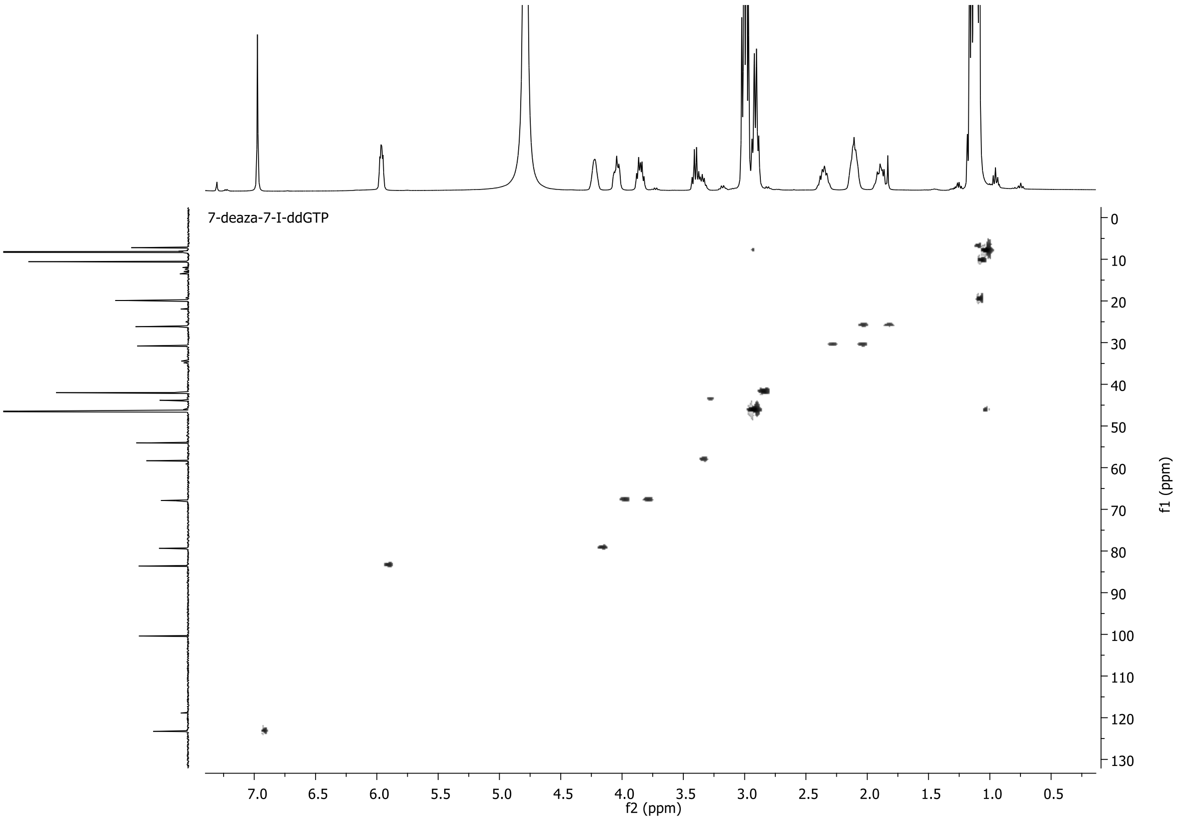

Supplementary Figure S82. H,C-HSQC spectra of **dd[I]GTP**

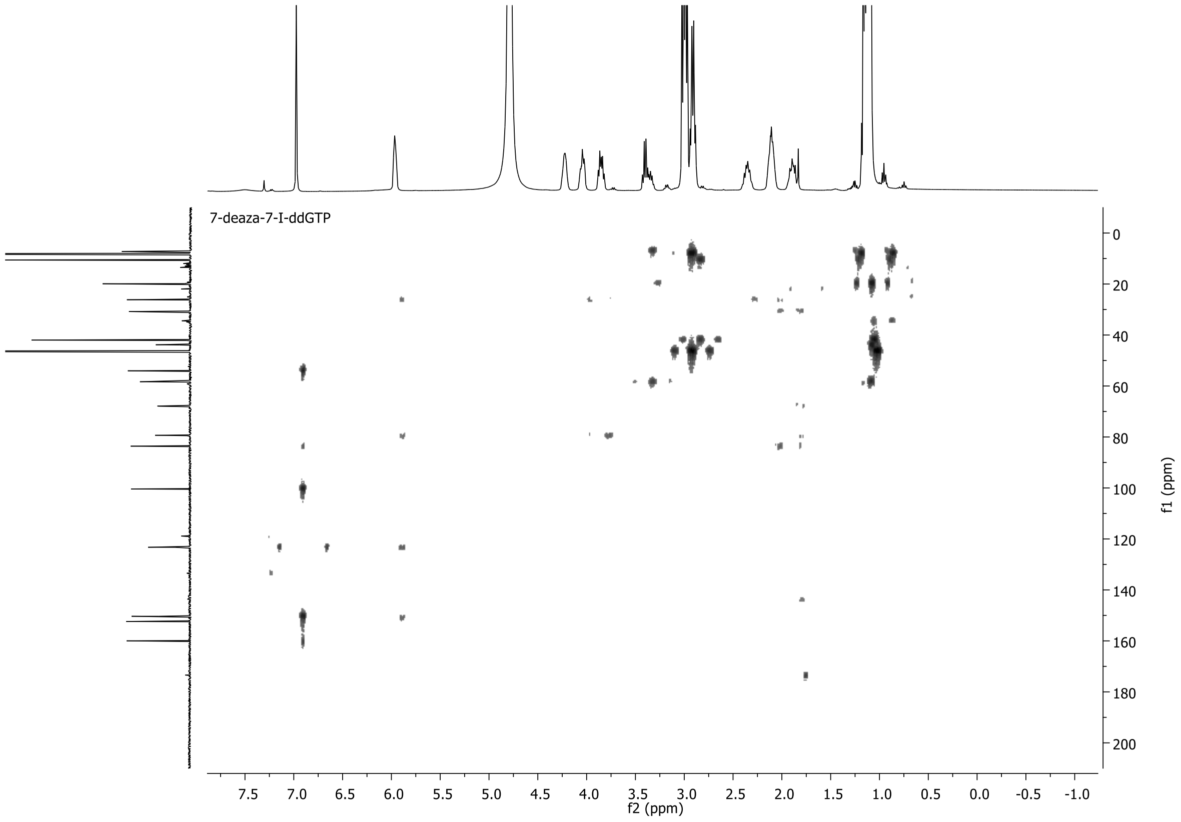

Supplementary Figure S83. H,C-HMBC spectra of **dd^{I}GTP**

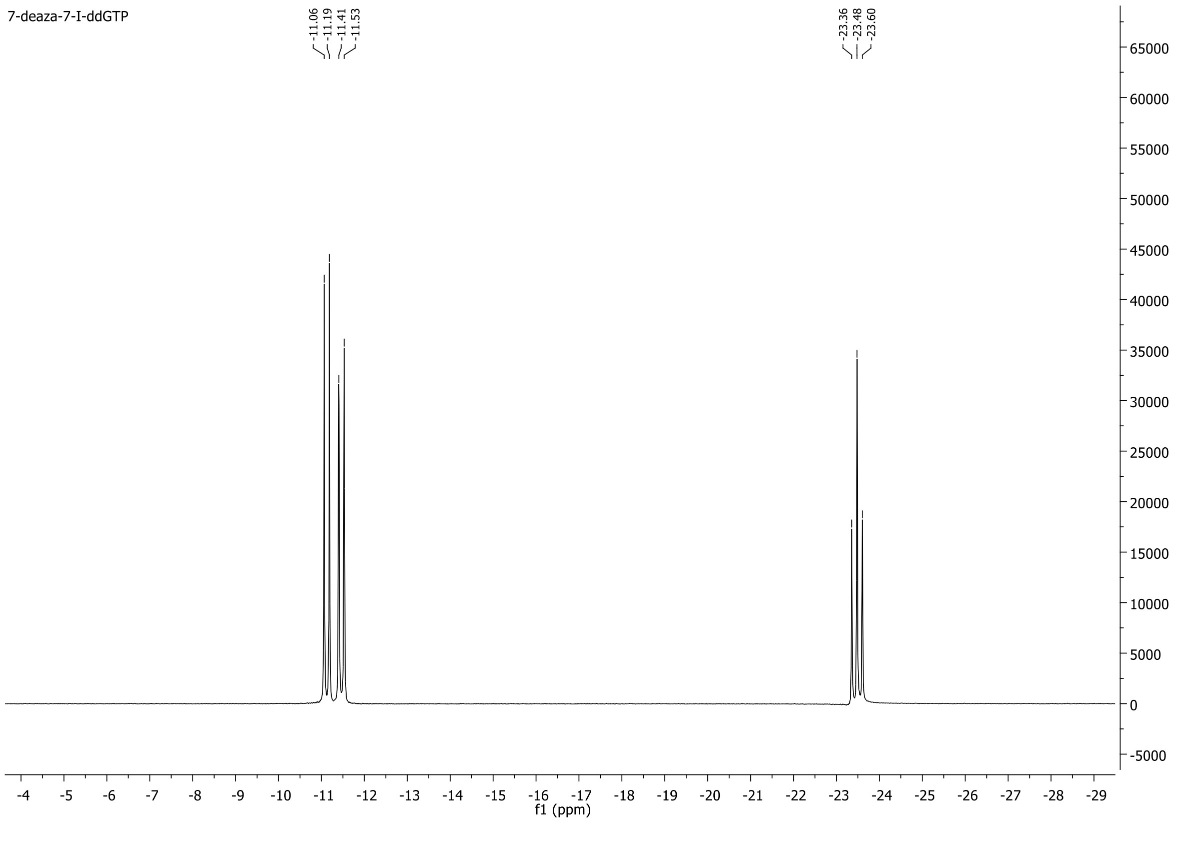

Supplementary Figure S84. ^{31}P spectra of **dd^{I}GTP**

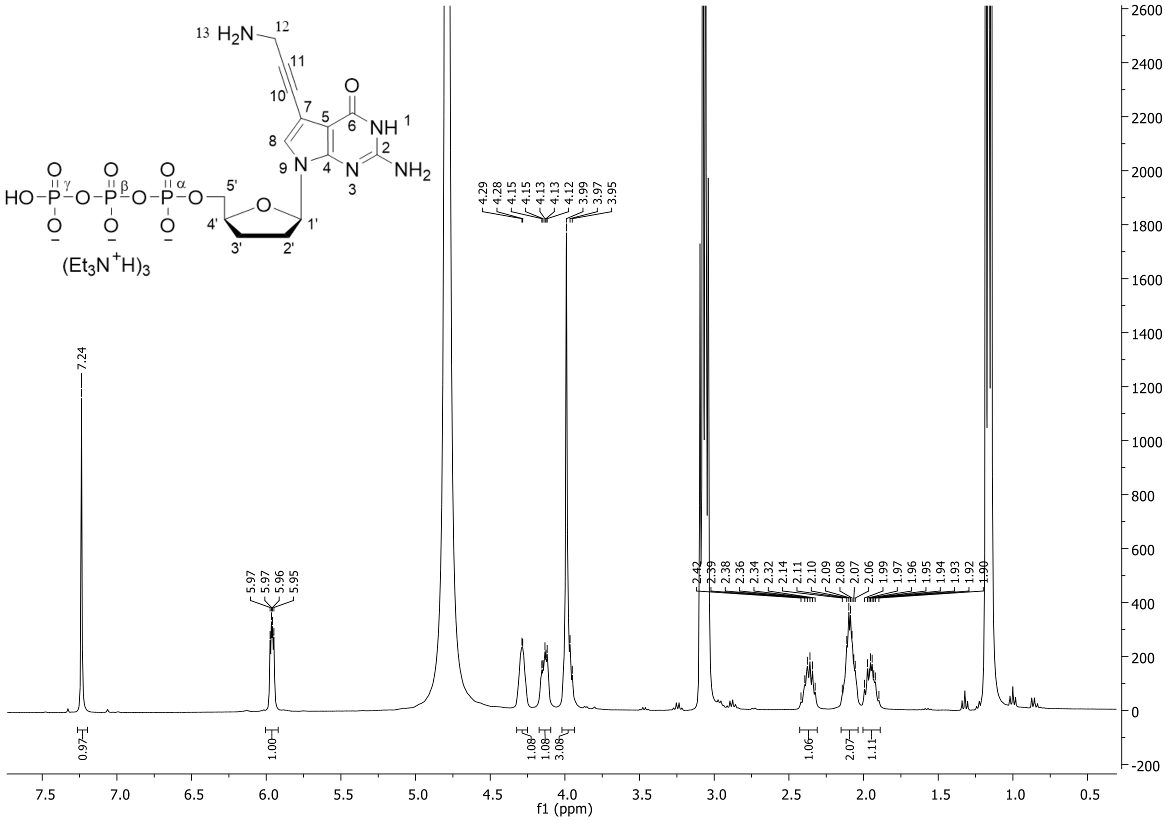

Supplementary Figure S85. ^{1}H spectra of **dd^{PA}GTP**

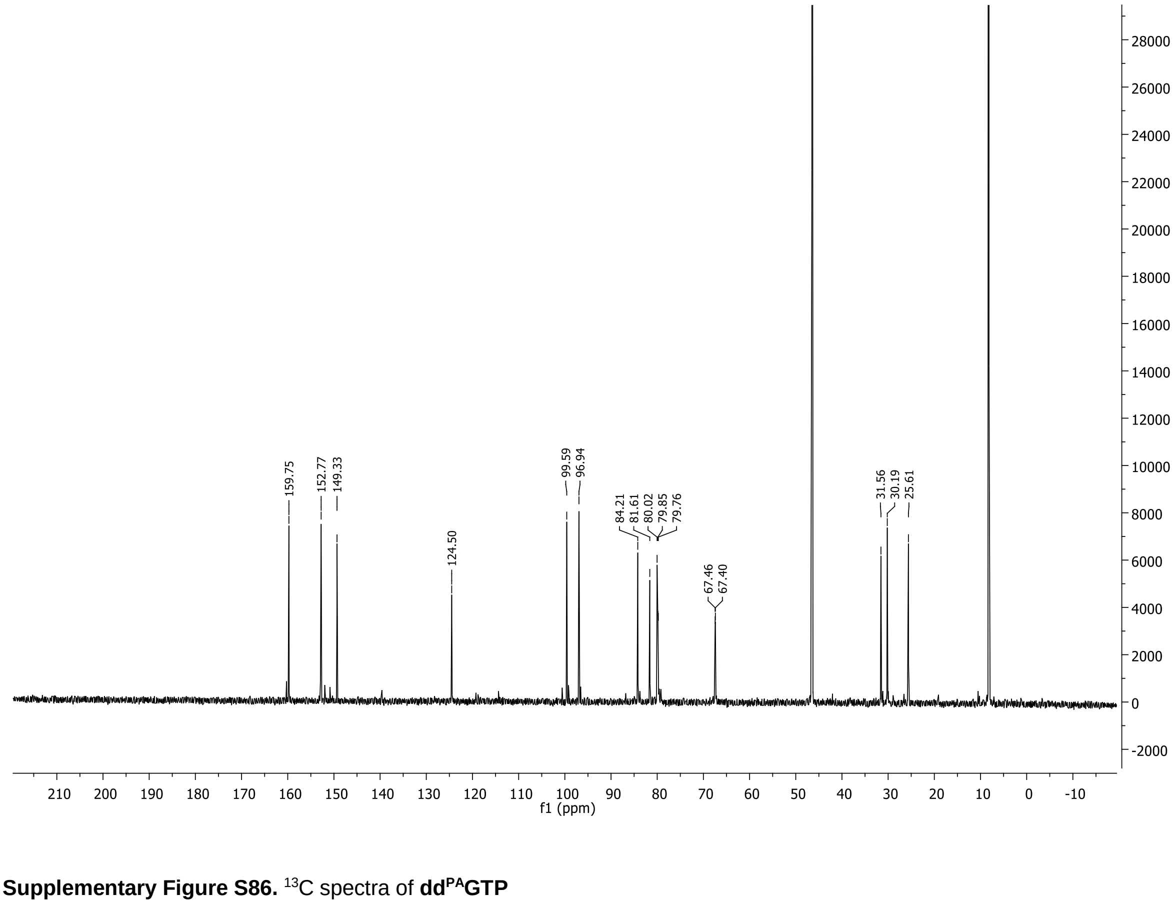

Supplementary Figure S86. ^{13}C spectra of **dd^{PA}GTP**

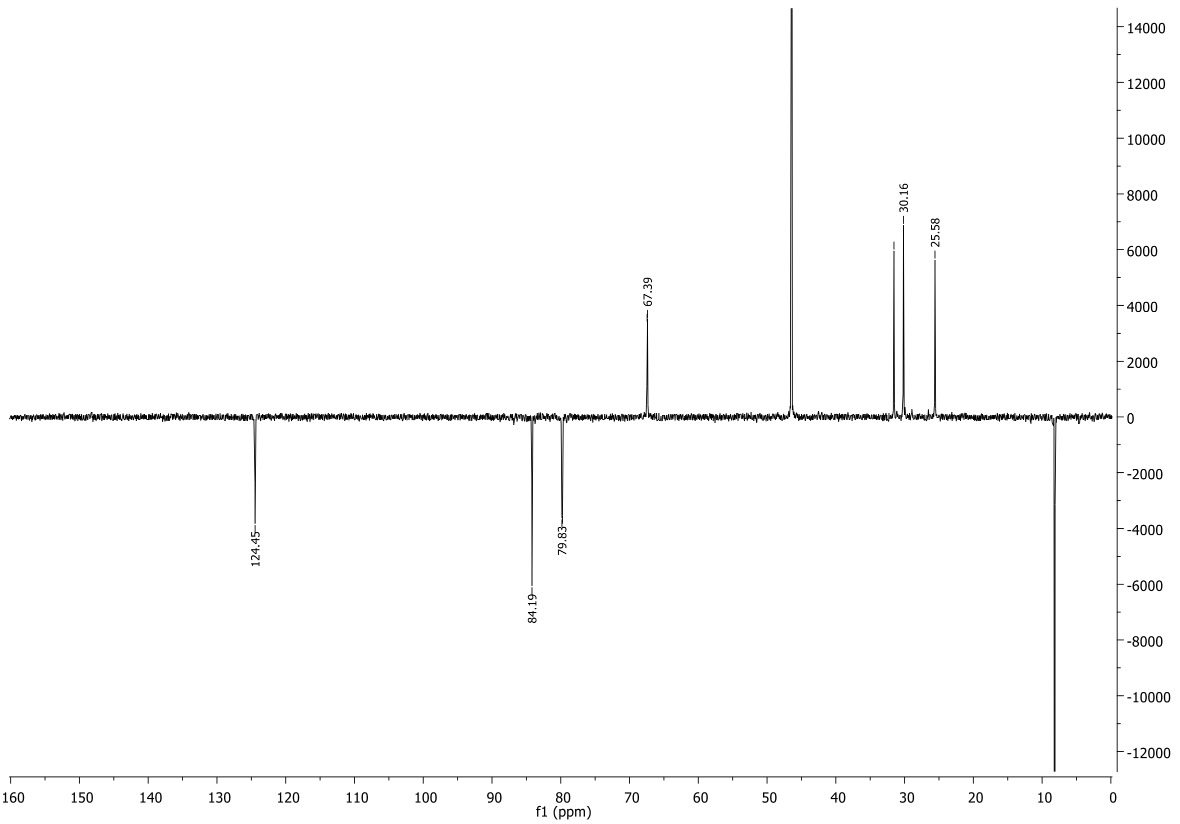

Supplementary Figure S87. ^{13}C Dept-135 spectra of **dd^{PA}GTP**

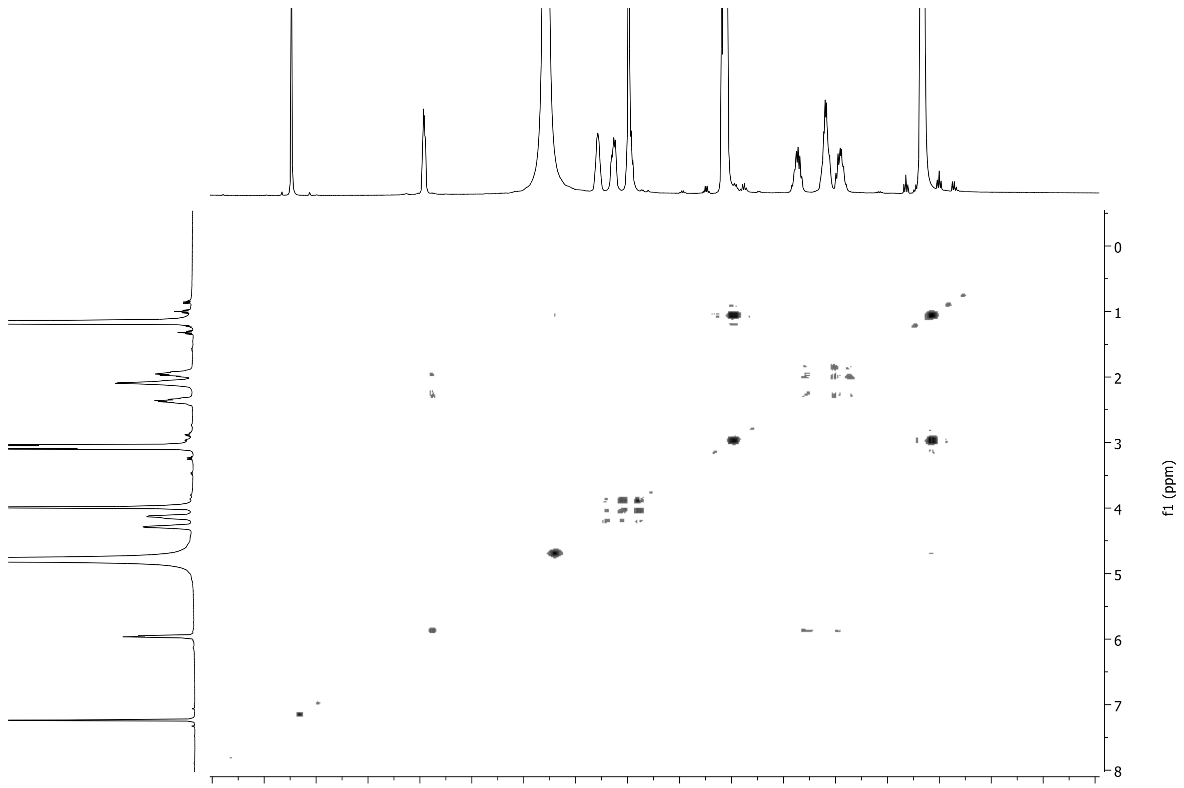

Supplementary Figure S88. H,H-COSY spectra of **dd^{PA}GTP**

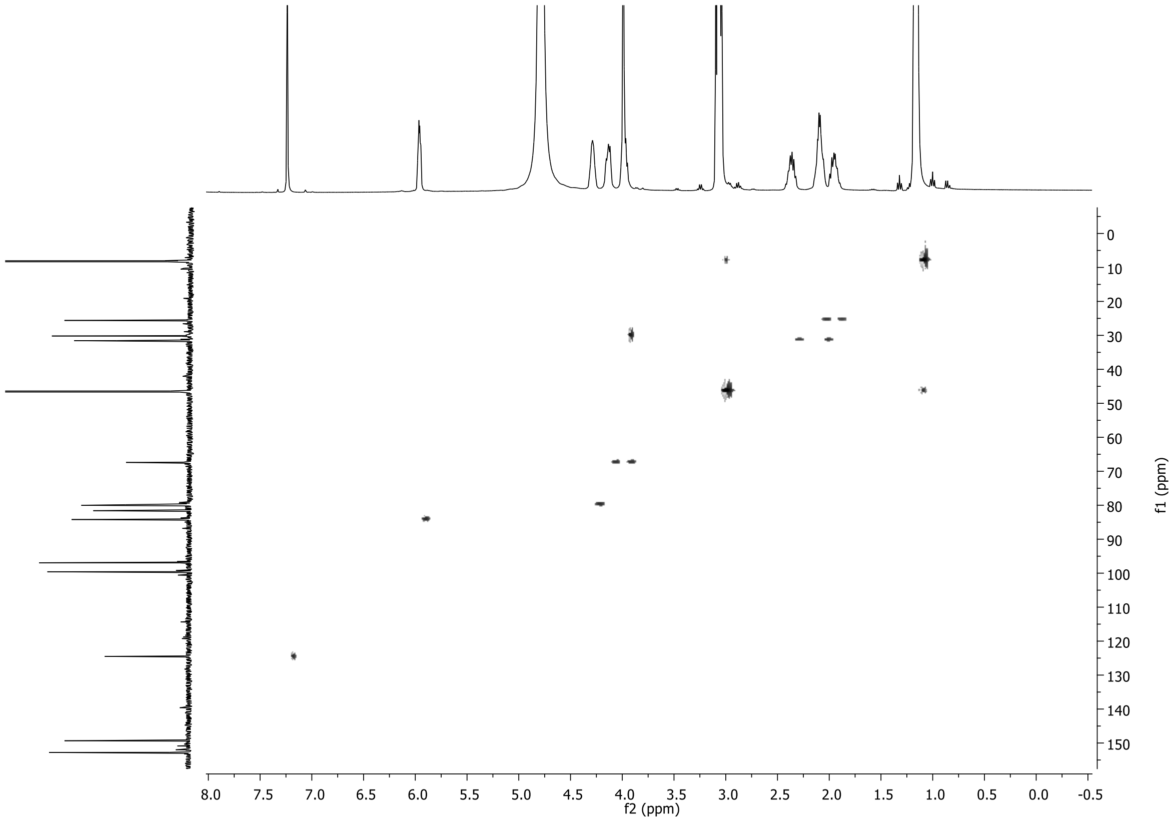

Supplementary Figure S89. H,C-HSQC spectra of **dd^{PA}GTP**

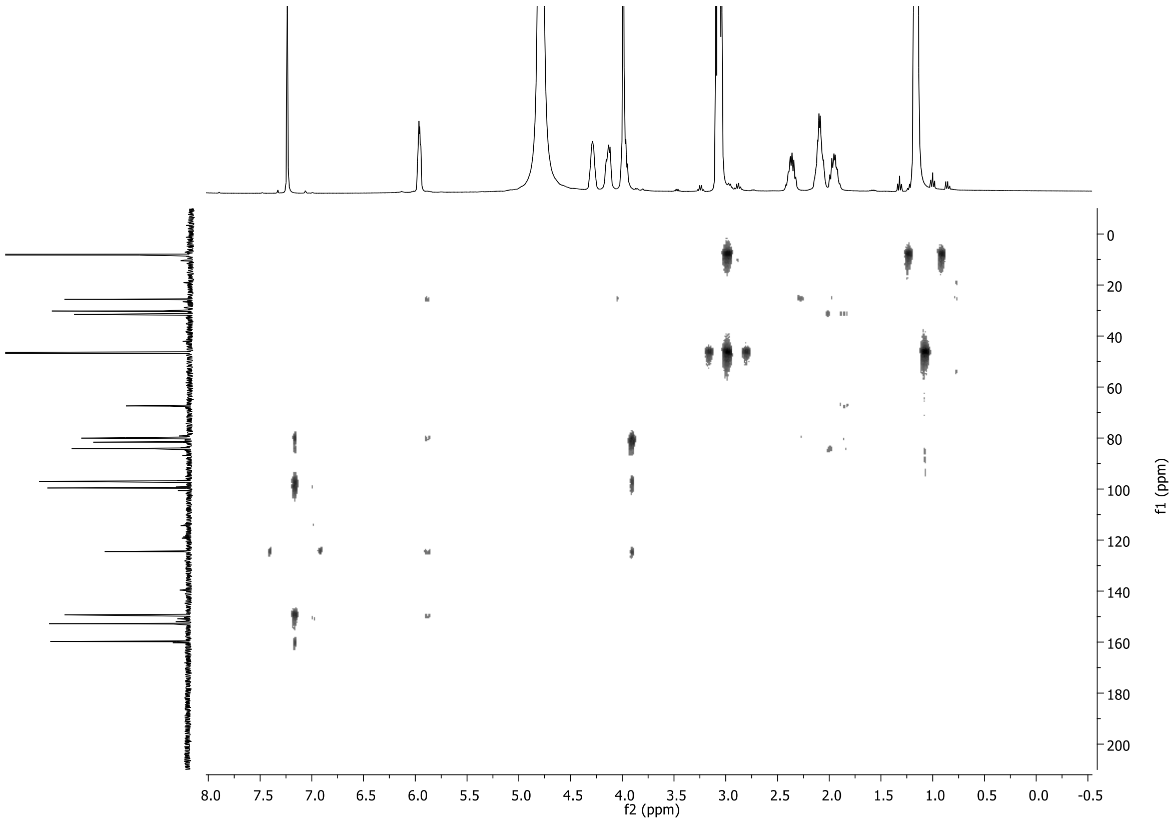

Supplementary Figure S90. H,C-HMBC spectra of **dd^{PA}GTP**

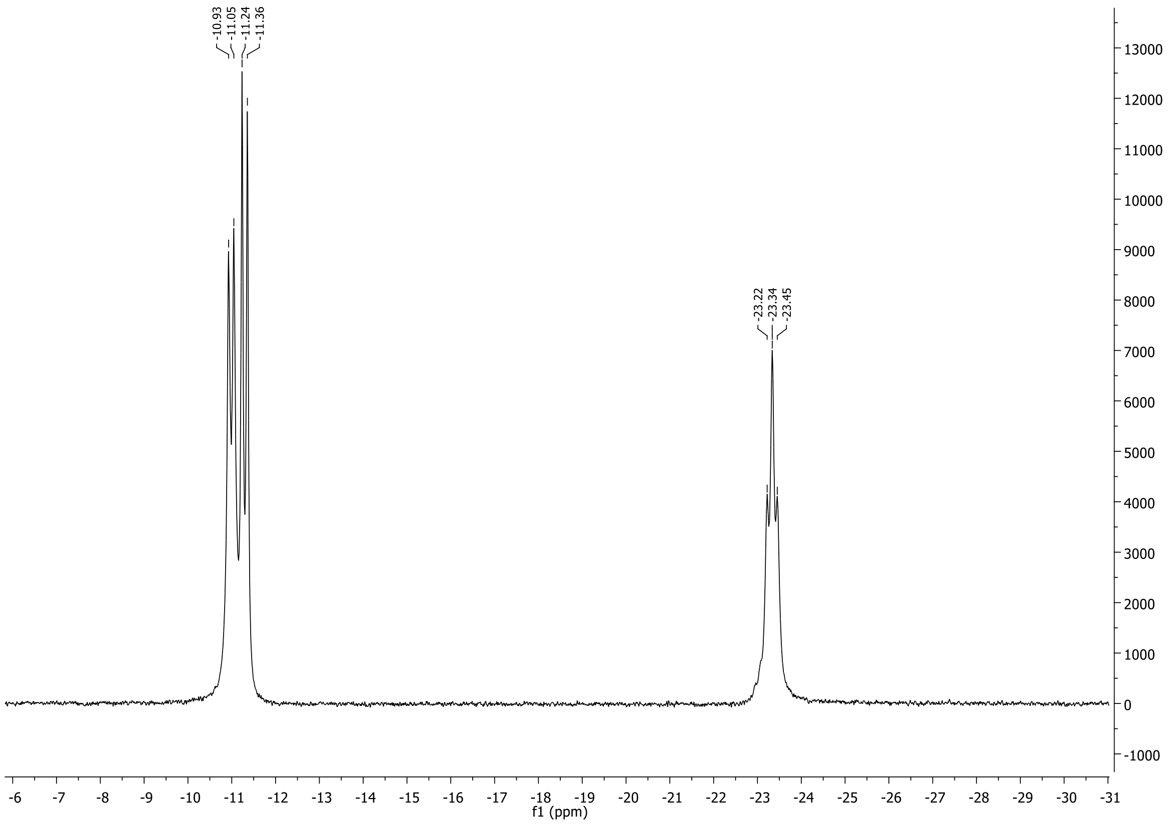

Supplementary Figure S91. ^{31}P spectra of **dd^{PA}GTP**

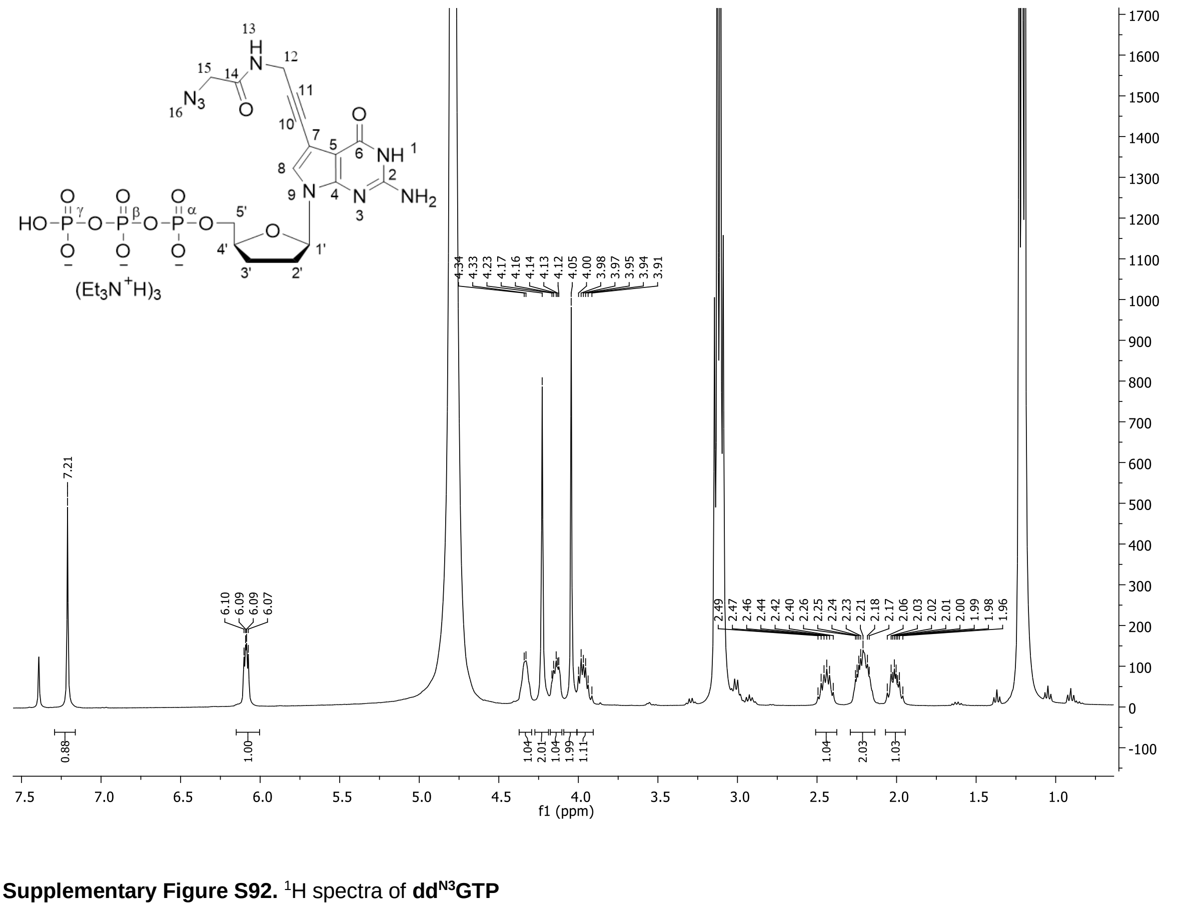

Supplementary Figure S92. ^{1}H spectra of **dd^{N3}GTP**

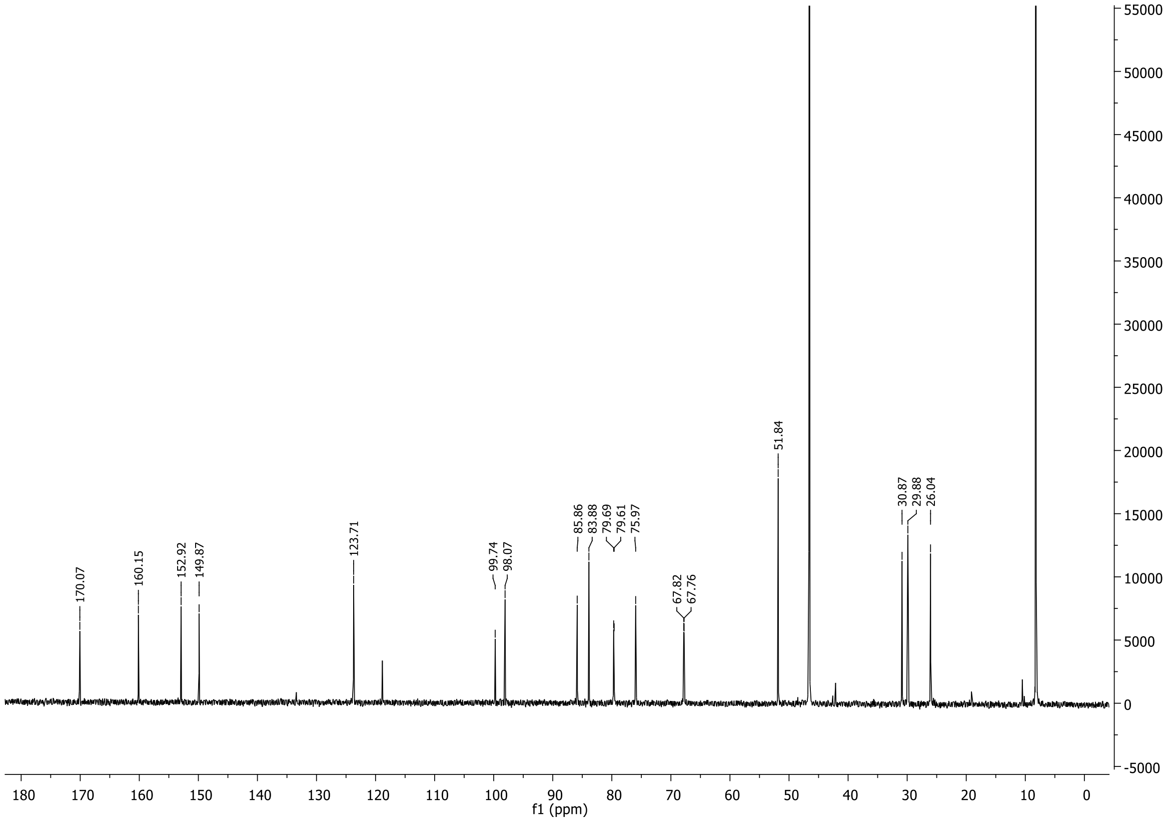

Supplementary Figure S93. ^{13}C spectra of **dd^{N3}GTP**

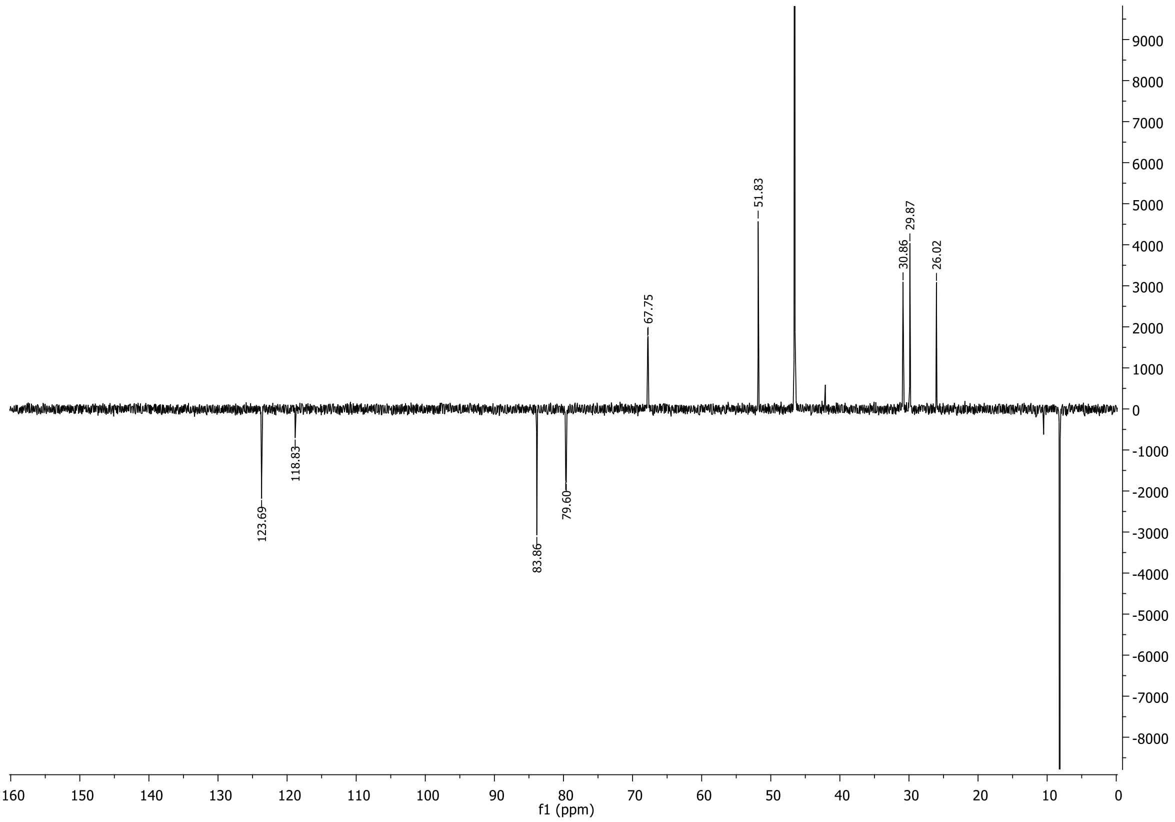

Supplementary Figure S94. ^{13}C Dept-135 spectra of **dd^{N3}GTP**

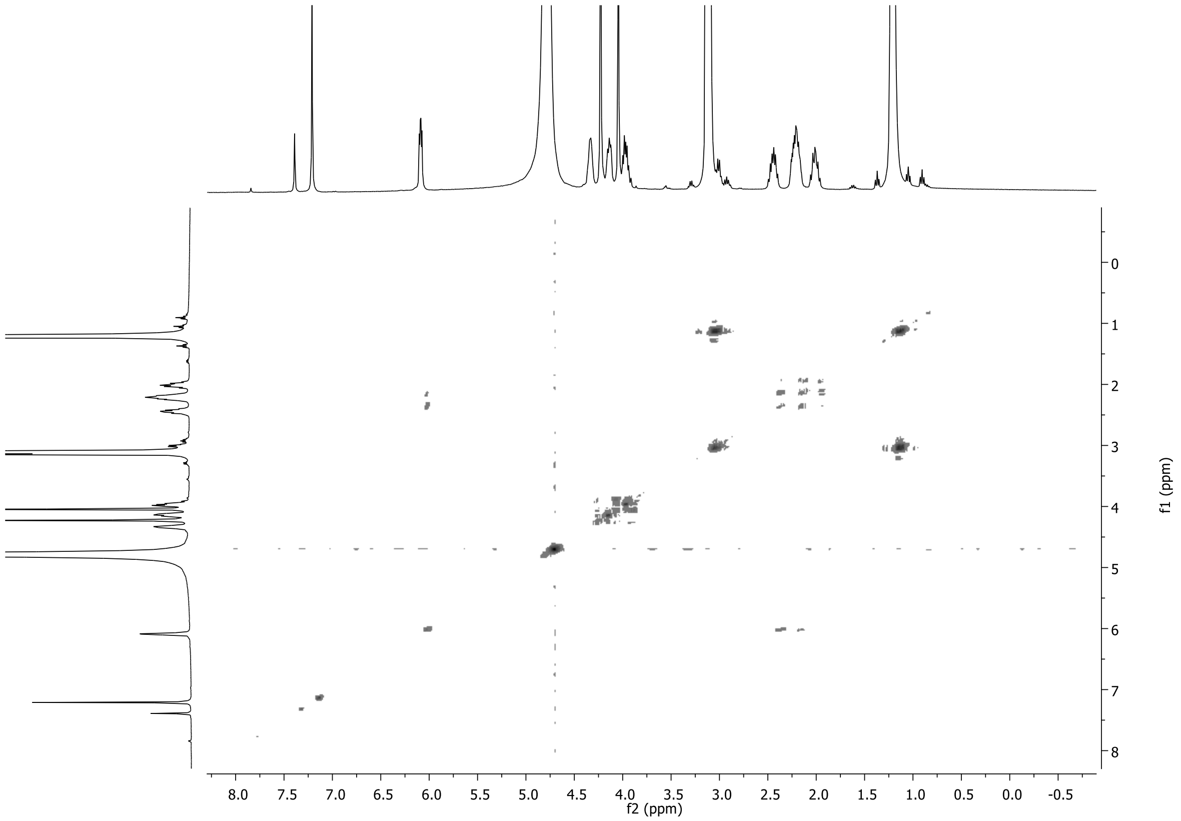

Supplementary Figure S95. H,H-COSY spectra of **dd^{N3}GTP**

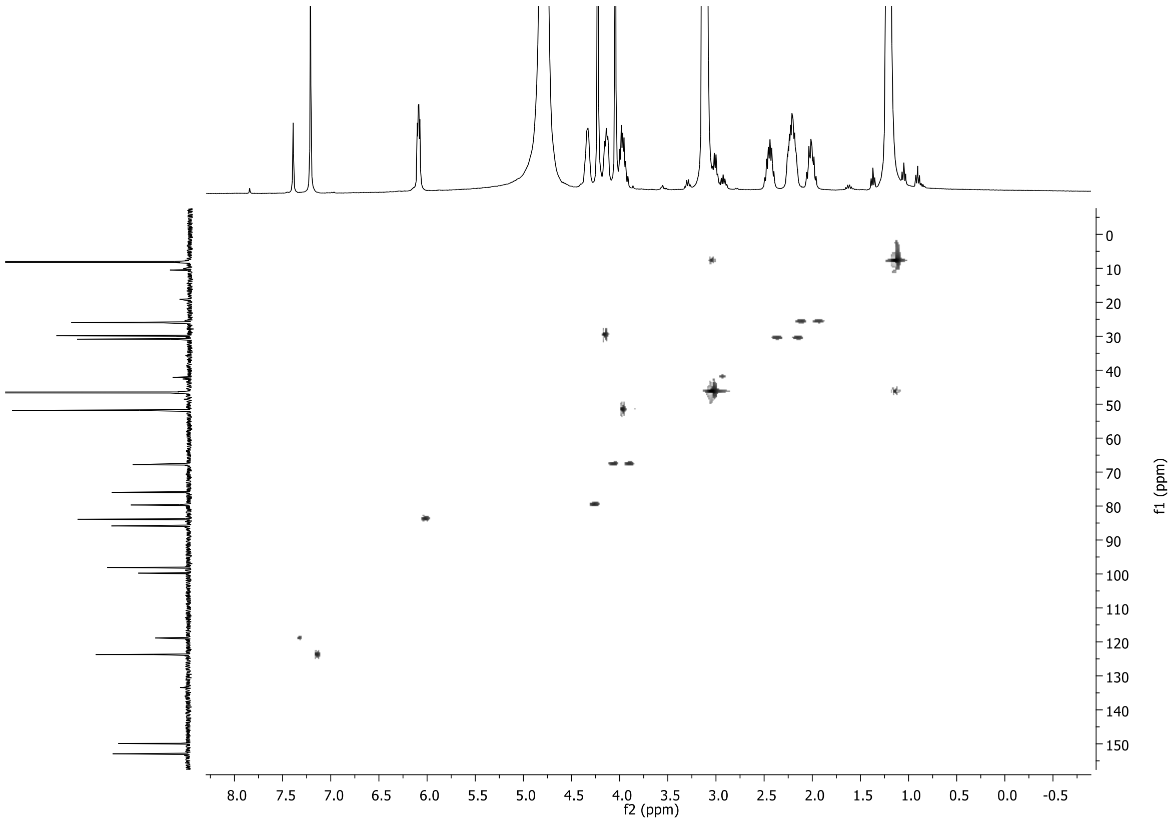

Supplementary Figure S96. H,C-HSQC spectra of **dd^{N3}GTP**

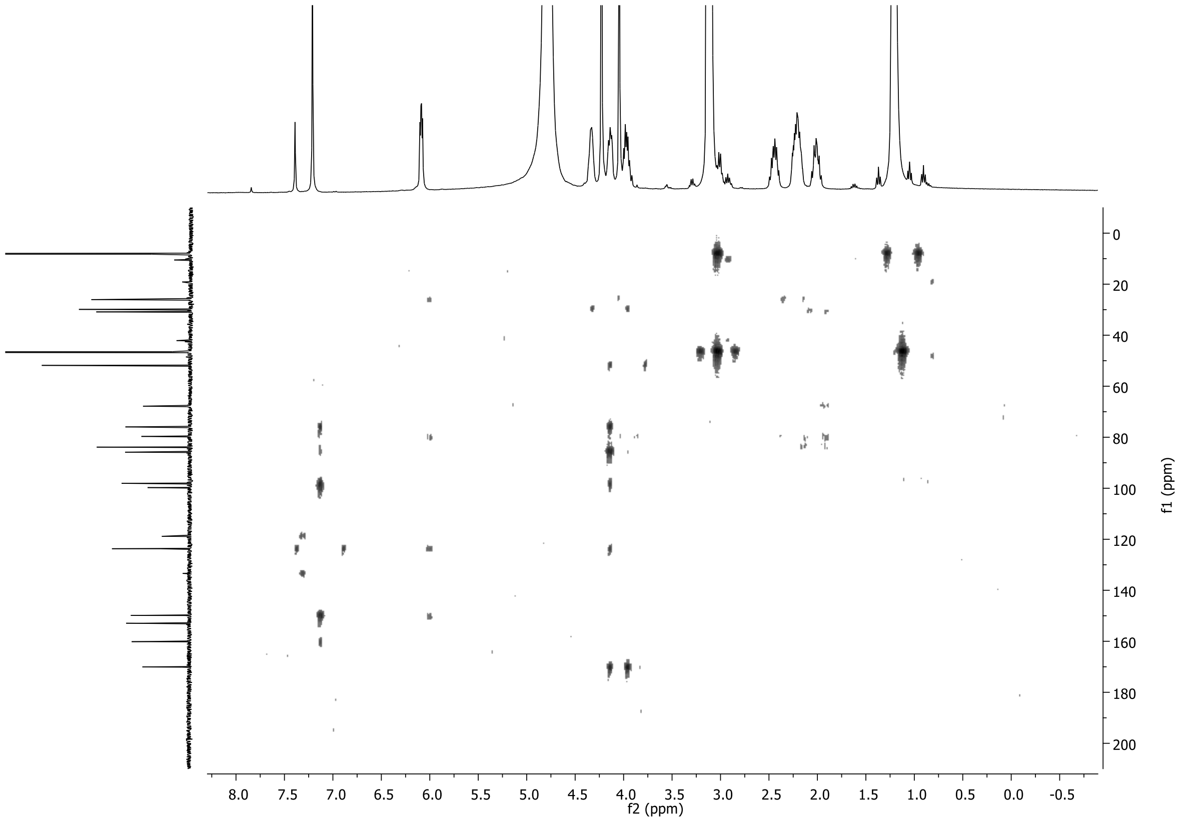

Supplementary Figure S97. H,C-HMBC spectra of **dd^{N3}GTP**

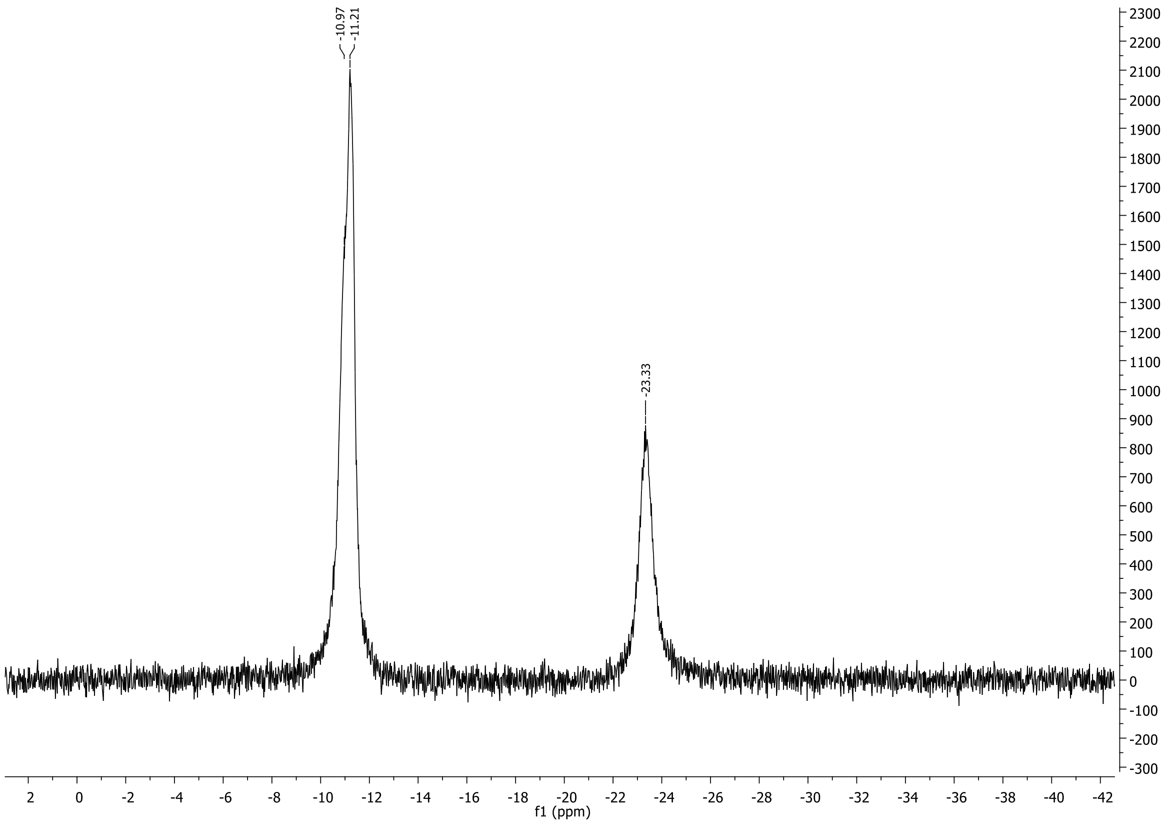

Supplementary Figure S98. ^{31}P spectra of **dd^{N3}GTP**
